# Reconstructed covalent organic frameworks

**DOI:** 10.1038/s41586-022-04443-4

**Published:** 2022-04-06

**Authors:** Weiwei Zhang, Linjiang Chen, Sheng Dai, Chengxi Zhao, Cheng Ma, Lei Wei, Minghui Zhu, Samantha Y. Chong, Haofan Yang, Lunjie Liu, Yang Bai, Miaojie Yu, Yongjie Xu, Xiao-Wei Zhu, Qiang Zhu, Shuhao An, Reiner Sebastian Sprick, Marc A. Little, Xiaofeng Wu, Shan Jiang, Yongzhen Wu, Yue-Biao Zhang, He Tian, Wei-Hong Zhu, Andrew I. Cooper

**Affiliations:** 1 Key Laboratory for Advanced Materials and Institute of Fine Chemicals, Joint International Research Laboratory of Precision Chemistry and Molecular Engineering, Feringa Nobel Prize Scientist Joint Research Center, Frontiers Science Center for Materiobiology and Dynamic Chemistry, School of Chemistry and Molecular Engineering, East China University of Science and Technology, Shanghai, China; 2 Leverhulme Centre for Functional Materials Design, Materials Innovation Factory and Department of Chemistry, University of Liverpool, Liverpool, UK; 3 State Key Laboratory of Chemical Engineering, East China University of Science and Technology, Shanghai, China; 4 School of Physical Science and Technology, ShanghaiTech University, Shanghai, China

**Keywords:** Organic molecules in materials science, Polymer synthesis, Photocatalysis, Porous materials

## Abstract

Covalent organic frameworks (COFs) are distinguished from other organic polymers by their crystallinity^1–3^, but it remains challenging to obtain robust, highly crystalline COFs because the framework-forming reactions are poorly reversible^4,5^. More reversible chemistry can improve crystallinity^6–9^, but this typically yields COFs with poor physicochemical stability and limited application scope^5^. Here we report a general and scalable protocol to prepare robust, highly crystalline imine COFs, based on an unexpected framework reconstruction. In contrast to standard approaches in which monomers are initially randomly aligned, our method involves the pre-organization of monomers using a reversible and removable covalent tether, followed by confined polymerization. This reconstruction route produces reconstructed COFs with greatly enhanced crystallinity and much higher porosity by means of a simple vacuum-free synthetic procedure. The increased crystallinity in the reconstructed COFs improves charge carrier transport, leading to sacrificial photocatalytic hydrogen evolution rates of up to 27.98 mmol h^−1^ g^−1^. This nanoconfinement-assisted reconstruction strategy is a step towards programming function in organic materials through atomistic structural control.

## Abstract (web summary)

A protocol in which monomers are pre-organized using a reversible and removable urea linkage enables the production of covalent organic frameworks with higher crystallinity and porosity than those produced using standard approaches with randomly aligned monomers.

## Main

Covalent organic frameworks (COFs) are of growing interest for gas storage, separation, electronics and catalysis applications because of their predictable structures and ordered nanopores^10–19^. Two-dimensional COFs with π-stacking between the layers allow for charge carrier transport in aligned molecular columns, and these materials show promise for photoenergy conversion and optoelectronics^20–31^. However, material quality and sometimes demanding synthetic procedures can limit practical applications. In particular, the moderate level of crystallinity in two-dimensional COFs can compromise their performance in optoelectronic applications, and synthetic requirements such as vacuum sealing or strictly anaerobic conditions are practical hurdles to scale-up.

COFs are typically prepared by simultaneous polymerization and crystallization of monomers following the principle of dynamic covalent chemistry^32^. Reversible bond formation and structural self-healing have a central role in achieving long-range crystalline order. Strategies have been reported to produce COFs using more reversible chemistry, even to the point of obtaining single crystals^6–9^, but not all of these strategies lead to porosity. High degrees of crystallinity are much more difficult to obtain when the framework bonding is more robust and less reversible^4,5,33^. As such, there is a trade-off between stability—which is desirable for practical applications—and high levels of long-range crystalline order. An attractive strategy is to pre-organize monomers before polymerization. This separates the crystallization process from the (irreversible) bond forming step^34–39^. In this case, monomers are pre-arranged in the solid state to form an ordered self-assembled structure before the polymerization reaction. However, the pre-organization is often based on weak intermolecular interactions, and strain induced by the change in geometry that occurs during polymerization can cause fragmentation of crystallites or structural disorder. Thus, such reactions tend to be limited to mild photo-polymerizations in which the change in geometry is not too large^34–36^, although more profound structural transformations are possible when flat and rigid building blocks are used^37,38^. The success of these solid-state transformation strategies relies on appropriate molecular ordering, which is hard to design a priori: for example, pre-organized non-covalent molecular crystals can form different polymorphs in different crystallization solvents, which can thwart reticular framework strategies.

**Fig. 1:**
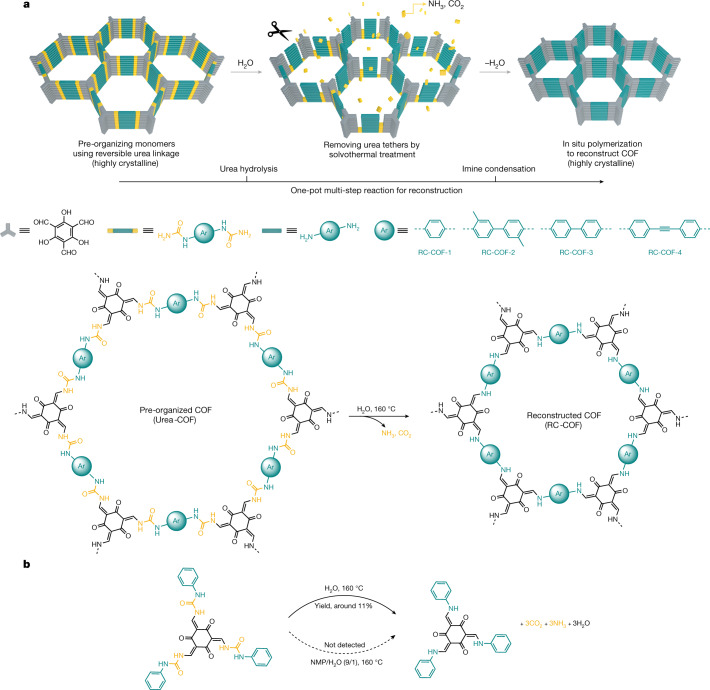
Chemical reconstruction. **a**, The synthetic procedure for reconstructed COFs includes two steps: pre-organization of the monomers using reversible urea linkages to form a highly crystalline framework, followed by a solvothermal treatment step that removes the urea tethers, releasing monomers that then undergo in situ polymerization to form the reconstructed β-ketoenamine COF. The urea linkage acts as a disposable tether in this one-pot multi-step reaction, organizing the monomers before being removed by conversion into ammonia and carbon dioxide gas. **b**, Transformation of the model compound. A small urea-linked model compound can also be converted into the corresponding β-ketoenamine product, but with low isolated yield (around 11% yield in the solid state in the presence of H_2_O; decomposition occurs when the model compound is dissolved in solution, NMP/H_2_O (9/1 v/v), and the β-ketoenamine product is not detected).

Confinement effects are ubiquitous in the chemistry of life; they prevent the denaturation of proteins and allow the synthesis of complex biomolecules under mild conditions. Likewise in synthetic nanochemistry, confining molecules can profoundly affect reaction pathways by stabilizing reactive species, accelerating reactions or enhancing selectivity^40,41^. Here we present a reconstruction strategy for COF synthesis that uses a reversible and removable covalent tether to pre-organize monomers before an irreversible polymerization. This route yields highly crystalline and functional COF materials through a facile process (Fig. 1a). By stepwise control over temperature and solvent, we achieved a chemical reconstruction in pre-organized urea-linked COFs. Instead of becoming amorphous, solvothermal treatment initiates a multi-step urea hydrolysis reaction followed by imine condensation. Notably, this generates a highly crystalline reconstructed COF (RC-COF) through a framework transformation, even though the mass loss during reconstruction can be as high as 36%. The position of the monomers that are produced by hydrolysis is directed by nanoconfinement in the framework before in situ polymerization. This results in greatly improved crystallinity and functional properties for the RC-COFs compared to directly polymerized imine frameworks, in which the monomers are aligned randomly before polymerization.

## Structural transformation

**Fig. 2:**
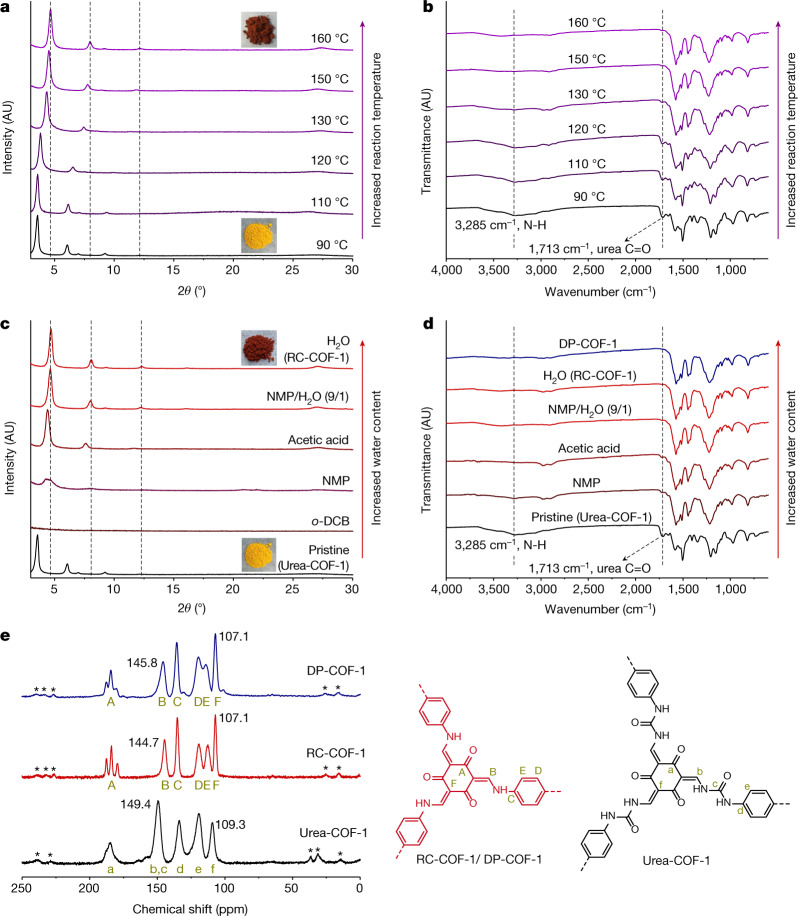
Thermal and water-triggered reconstruction. **a**, **b**, Evolution of the PXRD patterns (**a**) and FTIR spectra (**b**) for Urea-COF-1 as-synthesized at 90 °C and treated at the in situ increased reaction temperatures of 110, 120, 130, 150 and 160 °C for a further 72 h, respectively, in an 8/2/1 mixture of NMP, *o*-DCB and 6 mol l^−1^ acetic acid. The gradual shift in PXRD peak positions suggests a continuous structural transformation. Insets are photographs of isolated powders. AU, arbitrary units. **c**, **d**, Evolution of the PXRD patterns (**c**) and FTIR spectra (**d**) for isolated Urea-COF-1 treated by solvents with increased water content (*o*-DCB, NMP, glacial acetic acid, NMP/H_2_O (9/1 v/v) and H_2_O, respectively) at 160 °C for 72 h. Insets are photographs of Urea-COF-1 (yellow) and RC-COF-1 (dark red) powders. A comparison of the FTIR spectrum with that of DP-COF-1, which was synthesized by direct imine polycondensation, is also shown in **d**. **e**, ^13^C CP-MAS solid-state NMR spectra of Urea-COF-1, RC-COF-1 and DP-COF-1. Spinning sidebands are denoted with asterisks. Carbon atoms responsible for the NMR resonances are labelled A–F (for DP-COF-1 and RC-COF-1) and a–f (for Urea-COF-1). Source data

Urea chemistry is inexpensive and used in the large-scale manufacture of resins and adhesives. Urea-linked COFs have been synthesized previously^42^. Urea is quite stable, with a decomposition half-life of 3.6 years in aqueous solution (38 °C); in industry, hydrolysis of urea feedstocks into ammonia and carbon dioxide is used for ammonia supply. This hydrolysis reaction is favoured by increased temperatures. We therefore speculated that raising the temperature could promote a structural change in urea-linked COFs. Urea-COF-1 (also known as COF-117; ref. ^42^) was first synthesized via a Schiff-base condensation reaction of 1,1'-(1,4-phenylene)diurea with 1,3,5-triformylphloroglucinol in a mixture of *N*-methyl-2-pyrrolidinone (NMP), 1,2-dichlorobenzene (*o*-DCB) and aqueous acetic acid (6 mol l^−1^) at 90 °C for 72 h. Instead of isolating the powdered urea COF, we directly raised the temperature to 110, 120, 130, 150, 160 and 170 °C, respectively, for a further 72 h. A colour change from yellow to dark red was observed after raising the temperature (Fig. 2a), suggesting more extended electronic conjugation. The crystallinity of the solvated samples was assessed by powder X-ray diffraction (PXRD; Fig. 2a). When the reaction temperature was increased from 90 °C to 160 °C, the first intense diffraction peak was found to shift gradually from 2*θ* = 3.5° to 4.6°. An increase in the reaction temperature to 170 °C did not shift this peak any further (Supplementary Fig. 3).

**Extended Data Fig. 1:**
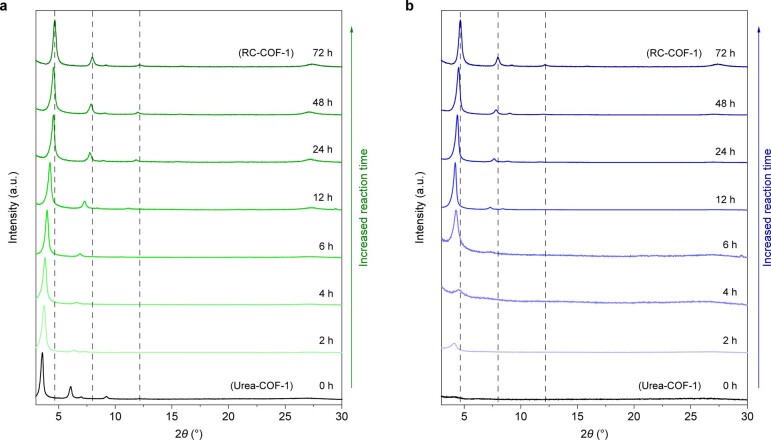
Continuous transformation in water. **a**, **b**, The evolution of the PXRD patterns with time for as-synthesized Urea-COF-1 by treatment with water at the reaction temperatures of 160 °C (samples were solvated with water (**a**) and measured after activation/desolvation (**b**)). The gradual shift in the peak positions over time suggests a continuous transformation during the COF reconstruction. Source data

Various solvents and solvent mixtures were investigated for this solvothermal treatment, such as *o*-DCB, NMP, glacial acetic acid and water. We first isolated the Urea-COF-1 powders formed at 90 °C after 72 h, and then used these solvents or solvent mixtures to treat the urea COF in a sealed Pyrex tube, separately, at a fixed temperature of 160 °C for a further 72 h (Fig. 2c). Urea-COF-1 decomposed in neat *o*-DCB and NMP, and little solid material was isolated after thermal treatment. In glacial acetic acid, most of the solid was retained and the first diffraction peak shifted to 4.4°, suggesting an incomplete phase transformation. When Urea-COF-1 was treated with pure water at 160 °C (Fig. 2c, Extended Data Fig. 1a), an intense diffraction peak appeared at 2*θ* = 4.6° and the colour of the powder changed from yellow to dark red (RC-COF-1). A small quantity of water in NMP (NMP/H_2_O; 9/1 v/v) also promoted this reconstruction. These experiments demonstrate that the transformation was induced by water, along with the increased temperature. A high concentration of ammonium ion was detected from the aqueous solution (Supplementary Fig. 4). Elemental analysis showed a marked reduction in the nitrogen content for RC-COF-1 relative to Urea-COF-1 (11.89 versus 16.75 wt%), and the experimental weight loss during transformation (36 wt%) was close to the proportion of urea in Urea-COF-1 (theoretical mass loss = 29 wt%), ignoring any end groups. We therefore hypothesized that the urea-linked COF had transformed into a β-ketoenamine COF by solvothermal treatment in water, with ammonia and carbon dioxide being released as by-products (Fig. 1). RC-COF-1 retained high crystallinity after thermal desolvation under dynamic vacuum, in contrast to Urea-COF-1, which has flexible linkages and loses crystallinity after solvent removal^42^ (Extended Data Fig. 1b).

To further confirm the structure of RC-COF-1, we synthesized the same β-ketoenamine COF by direct polymerization (DP-COF-1; also known as TpPa-1^4^) of 1,3,5-triformylphloroglucinol with *p*-phenylenediamine according to reported procedures. ^13^C cross-polarization magic angle spinning (CP-MAS) solid-state nuclear magnetic resonance (NMR) spectroscopy showed the same resonances for RC-COF-1 and DP-COF-1, although the peaks were narrower and better resolved in RC-COF-1, suggesting increased structural order^43^ (Fig. 2e). Fourier transform infrared (FTIR) spectroscopy for activated Urea-COF-1 showed strong bands at around 1,713 and 3,285 cm^−1^, corresponding to urea C=O and N–H groups (Fig. 2d). These bands disappeared after increasing the reaction temperature to 110–160 °C (Fig. 2b); this change was even more noticeable when we increased the water content in the solvent (Fig. 2d), suggesting hydrolysis of the urea groups. Elemental analysis (Supplementary Table 1) and X-ray photoelectron spectroscopy (Supplementary Fig. 6) also supported the solvothermal transformation of Urea-COF-1 to the β-ketoenamine COF, RC-COF-1.

## Improved crystallinity and surface area

**Fig. 3:**
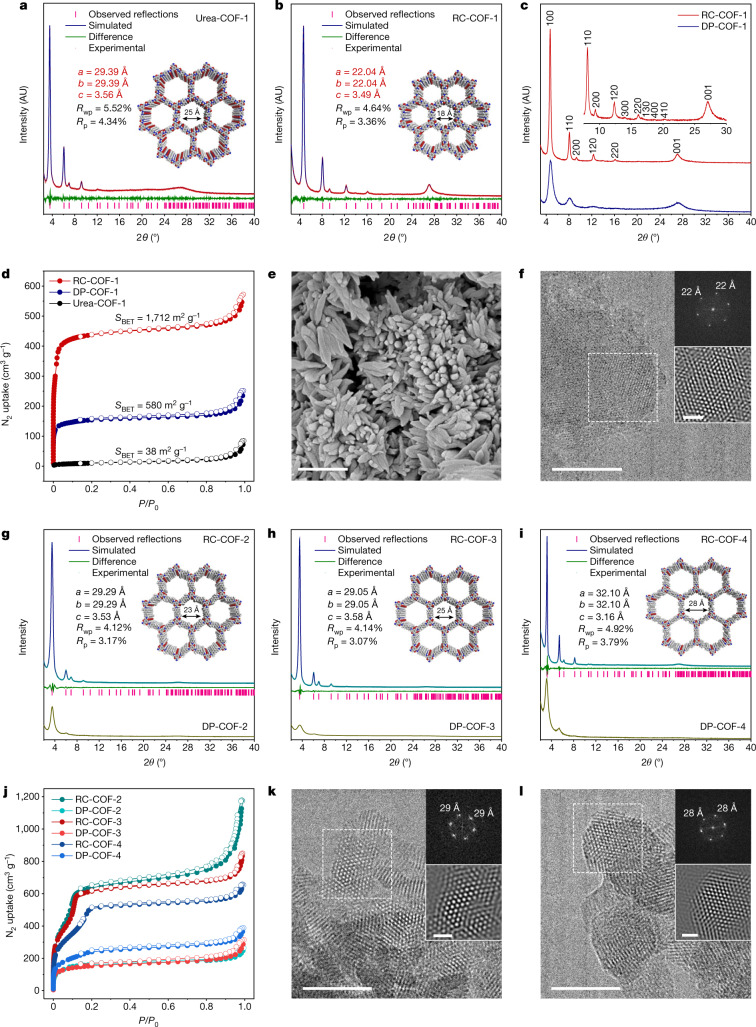
Reconstructed COFs with enhanced crystallinity and porosity. **a**, **b**, Simulated and experimental PXRD patterns for Urea-COF-1 (solvated) (**a**) and RC-COF-1 (activated) (**b**). The structural models were built using Materials Studio and refined using experimental PXRD data. **c**, Comparison of PXRD patterns for RC-COF-1 synthesized by the reconstruction protocol and DP-COF-1 synthesized by direct polymerization. **d**, Nitrogen adsorption isotherm (filled symbols) and desorption isotherm (open symbols) for RC-COF-1, DP-COF-1 and Urea-COF-1 recorded at 77.3 K; RC-COF-1 shows a type I isotherm. **e**, SEM image of RC-COF-1. Scale bar, 1 μm. **f**, HRTEM image of RC-COF-1. Insets show the FFT pattern taken from the regions highlighted by the dashed-line squares and the corresponding filtered inverse FFT image. Scale bars, 50 nm (main image); 10 nm (inset). **g**–**i**, Simulated and experimental PXRD patterns for RC-COF-2 (**g**), RC-COF-3 (**h**) and RC-COF-4 (**i**), and comparison with PXRD patterns of DP-COF-2, DP-COF-3 and DP-COF-4 synthesized by direct polymerization. **j**, Nitrogen adsorption isotherm (filled symbols) and desorption isotherm (open symbols) for RC-COF-2, RC-COF-3, RC-COF-4 and directly polymerized analogues. **k**, **l**, HRTEM images of RC-COF-2 (**k**) and RC-COF-3 (**l**). Insets show FFT patterns and the corresponding filtered inverse FFT images. Scale bars, 50 nm (main images); 10 nm (insets). Source data

The level of crystallinity in RC-COF-1 was markedly enhanced compared to its directly polymerized analogue, DP-COF-1 (Fig. 3c); RC-COF-1 showed prominent diffraction peaks at 4.6, 8.1, 9.3, 12.3, 14.0, 16.1, 16.6, 18.7, 20.3 and 27.1°, which were indexed as 100, 110, 200, 120, 300, 220, 130, 400, 410 and 001 reflections, respectively. By contrast, only four broad peaks could be discerned from the diffraction pattern of DP-COF-1, as measured using the same diffraction set-up and measurement conditions. Eclipsed AA-stacking models yielded PXRD patterns that were consistent with the experimental profiles of Urea-COF-1 and RC-COF-1 (Supplementary Figs. 9, 17). Pawley refinement in the *P*6/*m* space group with unit cell parameters of *a* = 29.39, *b* = 29.39, *c* = 3.56 Å (Fig. 3a) and *a* = 22.04, *b* = 22.04, *c* = 3.49 Å (Fig. 3b) reproduced the experimental curve with good agreement factors (weight-profile *R*-factor *R*_wp_ = 5.52% and unweighted *R*-factor *R*_p_ = 4.34% for Urea-COF-1, and *R*_wp_ = 4.64% and *R*_p_ = 3.36% for RC-COF-1), which suggested a pronounced contraction of the unit cell after reconstruction.

**Extended Data Fig. 2:**
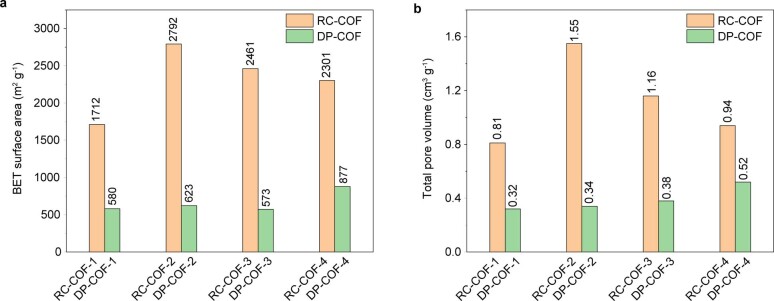
Increased porosity. **a**, **b**, Comparison of BET surface areas (**a**) and total pore volumes (**b**) for RC-COFs (yellow bars) and DP-COFs (green bars). Reconstructed COFs showed greatly improved BET surface area and pore volumes than directly polymerized analogues.

**Extended Data Fig. 3:**
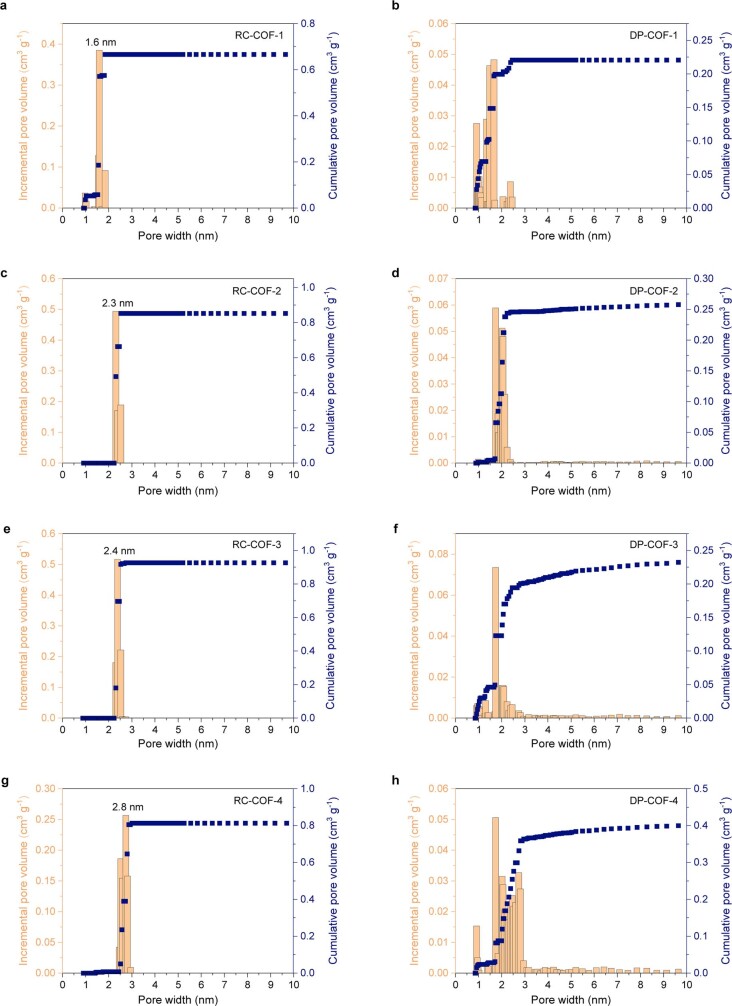
Pore size distributions. **a**–**h**, Pore size distribution profiles for RC-COFs (**a**, **c**, **e**, **g**) and DP-COFs (**b**, **d**, **f**, **h**). The reconstructed COFs showed a much narrower pore size distribution, which reflected their enhanced crystallinity. Source data

**Extended Data Fig. 4:**
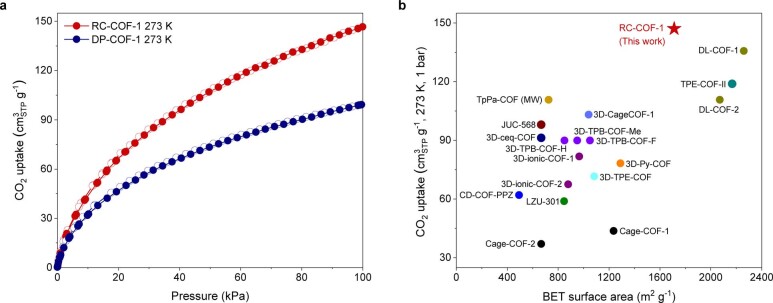
CO_2_ uptake. **a**, **b**, Adsorption (filled circles) and desorption (open circles) isotherms for CO_2_ uptake in RC-COF-1 and DP-COF-1 recorded at 273 K (**a**). Summary of CO_2_ uptake capacities for COF materials reported at 273 K and 1 bar (**b**); see details in Supplementary Table 5. Source data

**Extended Data Fig. 5:**
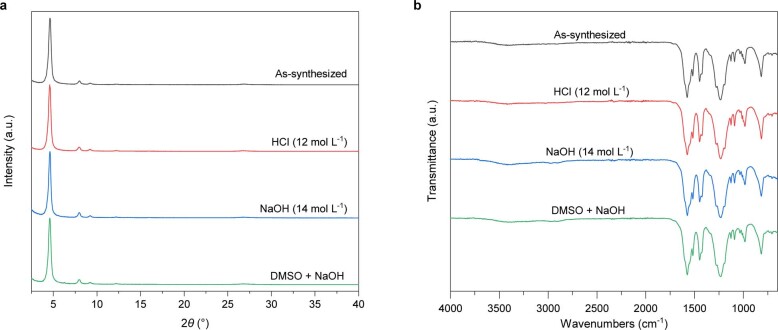
Chemical stability. **a**, **b**, PXRD patterns (**a**) and FTIR spectra (**b**) for RC-COF-1 after various chemical treatments for 24 h. RC-COF-1 retained its crystalline structure after treatment under all these conditions. Note that the PXRD peak intensity decreased slightly when treated with concentrated sodium hydroxide solution. Source data

The porosity of these COFs was evaluated by nitrogen adsorption measurements at 77.3 K (Fig. 3d). Urea-COF-1 showed a low Brunauer–Emmett–Teller (BET) surface area of 38 m^2^ g^−1^ because of pore deformation upon activation^42^. This increased to 1,712 m^2^ g^−1^ for RC-COF-1, which showed a type I gas adsorption isotherm with rapid gas uptake at low relative pressures (*P*/*P*_0_ < 0.01), indicating a highly microporous solid (Fig. 3d, Extended Data Fig. 2). The narrow pore size distribution of around 1.6 nm obtained from the adsorption isotherm using nonlocal density functional theory fitting was in precise agreement with the proposed structural model (Extended Data Fig. 3). By contrast, DP-COF-1 adsorbed much less gas (Fig. 3d) and showed a much lower BET surface area of 580 m^2^ g^−1^, close to previous reports for this material^4^ (535 m^2^ g^−1^). The broader, less regular pore size distribution for DP-COF-1 (Extended Data Fig. 3) can be ascribed to its semi-crystalline nature. The high crystallinity and regular porosity of RC-COF-1 also translated into high CO_2_ uptake, as shown by gas adsorption isotherms collected at 273 K (Extended Data Fig. 4a). RC-COF-1 showed a CO_2_ uptake of 147 cm^3^ g^−1^ (28.9 wt%) at 1 bar. This is to our knowledge the highest CO_2_ capacity reported in COFs under these measurement conditions^44^ (Extended Data Fig. 4b). The calculated heat of adsorption was around 35 kJ mol^−1^ at the adsorption onset (Supplementary Fig. 33), which is comparable to related small-pore COFs with high CO_2_ uptakes^44^. The directly polymerized analogue, DP-COF-1, showed a similar heat of adsorption but a much lower CO_2_ uptake (Supplementary Fig. 32). RC-COF-1 also showed excellent chemical stability after treatment with concentrated HCl (12 mol l^−1^) and NaOH (14 mol l^−1^) solution for 24 h (Extended Data Fig. 5).

Scanning electron microscopy (SEM) showed that RC-COF-1 comprised uniform rod-like crystallites with an average size of around 600 nm (Fig. 3e), whereas DP-COF-1 was composed of less regular aggregates (Supplementary Fig. 35c). The high crystallinity for RC-COF-1 allowed us to confirm its periodic porous structure using high-resolution transmission electron microscopy (HRTEM). Reticular structures with hexagonal pores oriented perpendicular to the crystallographic *c* axis were observed (Fig. 3f). The calculated distance between the centres of two adjacent pores was 2.2 nm, in good agreement with the refined eclipsed model. Fast Fourier transform (FFT) conducted on a selected area showed a hexagonal symmetry; by contrast, no lattice fringes were discerned for DP-COF-1 prepared by direct polymerization (Supplementary Fig. 37a).

**Extended Data Fig. 6:**
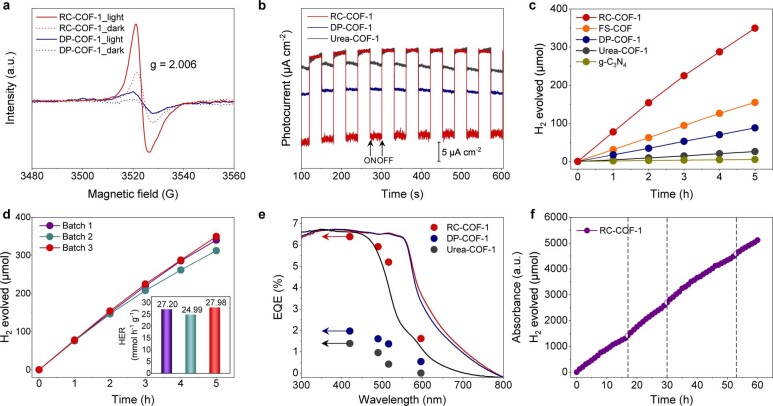
Optical and electronic properties and photocatalytic hydrogen evolution activity. **a**, EPR studies showed a single Lorentzian line centred at a *g* value of 2.006 for RC-COF-1, which intensified dramatically upon light excitation, suggesting an effective light-induced charge carrier generation, whereas DP-COF-1 displayed much lower signal intensity under same test conditions. **b**, Transient photocurrents with on-off light intermittent irradiation (*λ* > 420 nm) for RC-COF-1, DP-COF-1 and Urea-COF-1, conducted with a bias potential of – 0.35 V *vs* Ag/AgCl. RC-COF-1 produced an enhanced photocurrent compared to its semi-crystalline counterpart, DP-COF-1, indicating more efficient separation of photogenerated charge carriers. **c**, Time courses of sacrificial photocatalytic hydrogen production for RC-COF-1, FS-COF, DP-COF-1, Urea-COF-1 and g-C_3_N_4_ (2.5 mg catalyst in water with 3 wt.% Pt loading, *λ* > 420 nm for RC-COF-1, FS-COF, DP-COF-1 and Urea-COF-1, and *λ* > 295 nm for g-C_3_N_4_). **d**, Time course of photocatalytic hydrogen evolution for RC-COF-1 from three different synthetic batches under visible light irradiation; inset is corresponding hydrogen evolution rate (HER). There is good batch-to-batch reproducibility in terms of photocatalytic performance for materials prepared by this reconstruction route. **e**, The external quantum efficiencies (EQEs) of RC-COF-1 were estimated to be 6.39% at 420 nm, 5.92% at 490 nm, 5.20% at 515 nm, and 1.62% at 595 nm, respectively. By comparison, DP-COF-1 exhibited a much-lower EQEs of 1.97%, 1.61%, 1.37%, and 0.54% at the same wavelengths. **f**, Long-term photocatalytic hydrogen evolution stability test for RC-COF-1 over 60 h under visible light (*λ*  >  420 nm). The dashed vertical lines denote degassing and addition of a further 1.25 mmol of ascorbic acid. No obvious decrease in activity was observed during this 60-h period. Source data

**Extended Data Fig. 7:**
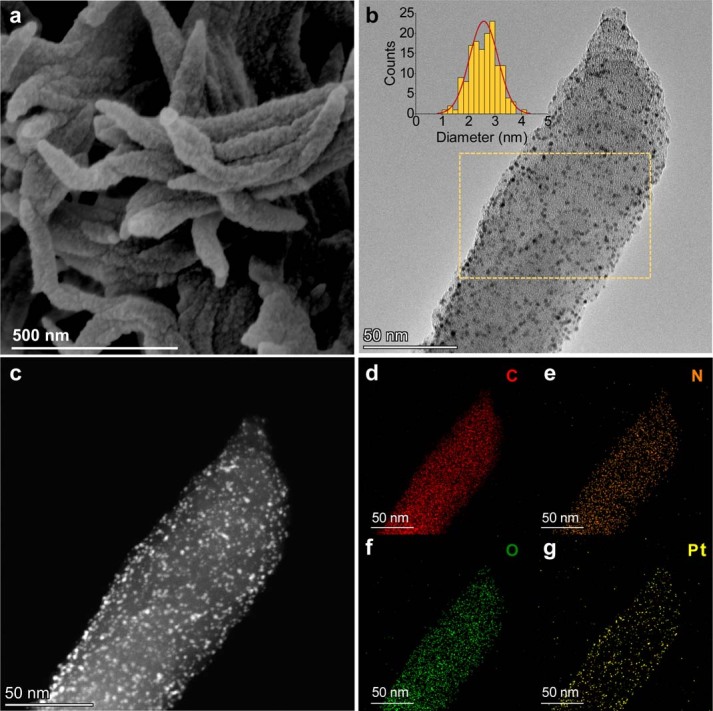
RC-COF-1 crystallite with photo-deposited Pt co-catalyst nanoparticles. **a**–**g**, SEM (**a**), TEM (**b**), high angle annular dark field scanning transmission electron microscopy (HAADF-STEM) (**c**) images and elemental mapping (**d**–**g**) for RC-COF-1 crystallite decorated with photo-deposited Pt co-catalyst. The inset in **b** shows uniform distributions of Pt nanoparticles (2.5 ± 0.5 nm) in the selected area (yellow square). The uniform morphology of the reconstructed COF and the good Pt cocatalyst dispersion might also contribute to its enhanced activity. Source data

We next considered the generality of this reconstruction protocol for other COFs. For Urea-COF-2 (also known as COF-118; ref. ^42^), we used a commercially available isocyanate as the starting material; for Urea-COF-3 and Urea-COF-4, we used more widely accessible arylamine monomers, which could be easily converted into diureas before pre-organization (Fig. 1, Supplementary Fig. 7). Again, these three reconstructed COFs (RC-COF-2, RC-COF-3 and RC-COF-4) all showed superior crystallinity relative to COFs that were prepared by direct polymerization as per previously reported procedures (DP-COF-2 (also known as TpBD-Me_2_; ref. ^45^), DP-COF-3 (or TpBD; ref. ^46^) and DP-COF-4 (or TP-EDDA; ref. ^24^)), and all showed sharp and well-resolved diffraction peaks (Fig. 3g–i). Indeed, the difference in crystallinity levels between the pre-organized, reconstructed COF and its direct polycondensation analogue was even more pronounced for the mesoporous COF, RC-COF-4, which has the largest pores in this series of materials. Nitrogen adsorption measurements revealed greatly increased surface areas and pore volumes for the reconstructed COFs (Fig. 3j, Extended Data Fig. 2); BET surface areas increased from 623 m^2^ g^−1^ (DP-COF-2) to 2,792 m^2^ g^−1^ (RC-COF-2); from 573 m^2^ g^−1^ (DP-COF-3) to 2,461 m^2^ g^−1^ (RC-COF-3); and from 877 m^2^ g^−1^ (DP-COF-4) to 2,301 m^2^ g^−1^ (RC-COF-4). As such, the surface areas of the reconstructed COFs were between 2.6 and 4.5 times larger than the directly polymerized analogues. Likewise, the measured pore volumes were two to four times higher for the reconstructed COFs than for directly polymerized analogues. Pore size distribution profiles indicated the mesoporous nature of RC-COF-2, RC-COF-3 and RC-COF-4, with pore sizes of 2.3, 2.4 and 2.8 nm, respectively, in precise agreement with their structural models; whereas directly polymerized COFs show broader pore size distributions (Extended Data Fig. 3). HRTEM images (Fig. 3k, l, Supplementary Fig. 36) showed ordered porous structures extending through the crystal domains with clearly visible honeycomb pores, and the periodicities were consistent with the unit cell derived from the Pawley refined PXRD data. Few such ordered domains could be observed in the directly polymerized analogues (Supplementary Fig. 37). The reconstructed COFs also showed better thermal stability than the directly polymerized analogues, presumably because of their enhanced crystallinity (Supplementary Fig. 34). The increased crystallinity in RC-COF-1 improves photogenerated charge carrier transport, leading to sacrificial photocatalytic hydrogen evolution rates of up to 27.98 mmol h^−1^ g^−1^. This is one of the highest activities reported for a COF photocatalyst and four times higher than the chemically equivalent but less ordered DP-COF-1 (Extended Data Figs. 6, 7, Supplementary Information).

## Density functional theory calculations

**Fig. 4:**
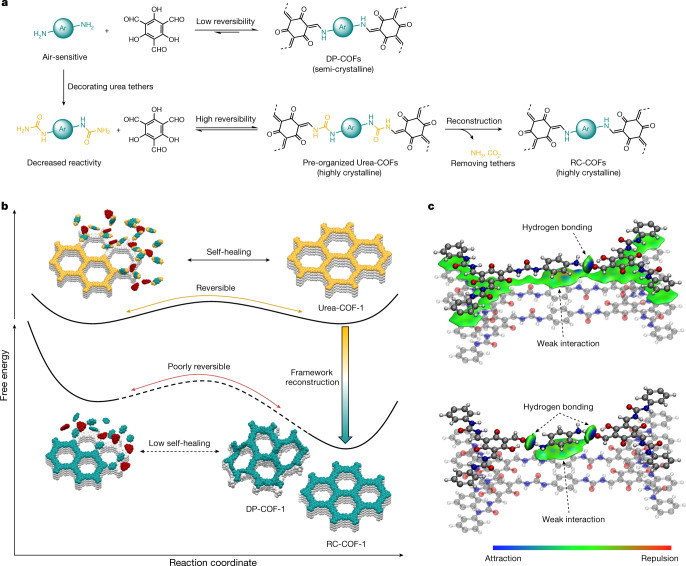
Reconstruction protocol with DFT calculations. **a**, **b**, Scheme showing the reaction paths for direct imine polycondensation and reconstruction synthesis (**a**). Direct polymerization yields only semi-crystalline COFs owing to the poor reversibility of the β-ketoenamine linkage, whereas decoration with urea groups decreases the reactivity and increases the reversibility, which leads to highly crystalline Urea-COFs (**b)**; these then undergo framework reconstruction into β-ketoenamine RC-COFs with retained crystallinity. **c**, DFT-optimized geometries of phenylene amine molecules released by hydrolysis, confined on the surface of the reconstructing COF. The results of hydrolysis of a single urea bond (top) and both urea bonds (bottom) are shown. Coloured isosurfaces are the intermolecular interactions quantified by an independent gradient model (isosurface = 0.003 atomic units). In the bottom example, the interactions between the COF layers are omitted to highlight the interaction between the *p*-phenylenediamine monomer and the framework.

Density functional theory (DFT) calculations were used to investigate this reconstruction protocol in more detail. Direct polymerization of 1,3,5-triformylphloroglucinol with *p*-phenylenediamine yields rather low crystallinity because of the poorly reversible bond formation and the tautomerization into a stable β-ketoenamine form^4^ (Fig. 4a, Supplementary Fig. 42a), which does not allow for full error correction. As a result, the directly polymerized product, DP-COF-1, does not attain the crystalline thermodynamic minimum structure (Fig. 4b). By contrast, decorating arylamine monomers with urea groups decreases the reactivity and enhances the reversibility for the bond formation (Supplementary Fig. 42b). This yields a highly crystalline but ‘soft’ urea precursor framework. Typically, hydrolysis of crystalline frameworks might lead to amorphization, but here, under appropriate solvothermal conditions (Fig. 2c), confinement in the framework coupled with fast imine condensation leads to the retention of crystallinity, together with a high conversion yield. The nature of the reconstruction process is revealed by the evolution of PXRD patterns recorded at different time intervals (Extended Data Fig. 1a). The diffraction peaks were found to shift continuously^47^, with no detectable disorder during the reconstruction. We suggest that the reconstruction in each COF crystallite has relatively slow kinetics with respect to the PXRD collection timescale. The gradual shift in peak positions indicates a smooth, continuous shrinkage of the lattice upon hydrolysis and re-polymerization. By contrast, if a rapid and concerted phase transformation was occurring, then we would expect to observe two distinct sets of interconverting PXRD peaks^48^, and no intermediate phases would be observed.

The simulation in Fig. 4c suggests well-defined non-covalent interactions in the framework when the urea linkage is hydrolysed. When a single urea bond is cleaved (Fig. 4c), strong hydrogen bonding is found in these simulations at the hydrolysis position between the resulting amine group and fragments of the COF in the same layer, which maintains the position of the molecule in the framework. When both urea bonds are cleaved (Fig. 4c), the *p*-phenylenediamine molecule that is produced by hydrolysis is still captured by the framework through hydrogen bonds. The π-π stacking between *p*-phenylenediamine and the adjacent COF layer reinforces these interactions (binding energy of around –16.08 kcal mol^−1^). We suggest that this nanoconfinement in the framework also stabilizes and protects the amine species by preventing the entry of other reactive species, contributing to the high yield of this multi-step reconstruction reaction. By contrast, such confinement is absent for the small-molecule model compounds (Fig. 1b) and the monomers can become disordered or react with other solution species once the urea linkages are hydrolysed.

## A convenient synthetic route

**Extended Data Fig. 8:**
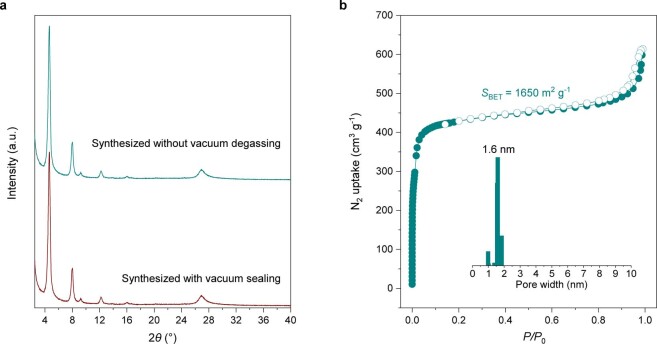
RC-COF-1 synthesized without vacuum degassing step. **a**, **b**, Experimental PXRD patterns (**a**) and nitrogen adsorption isotherm (**b**, filled symbols) and desorption isotherm (**b**, open symbols) for RC-COF-1 synthesized without vacuum degassing steps; inset shows calculated pore size distribution. Both the crystallinity and the porous properties are close to those obtained with careful degassing, suggesting that this step is unnecessary in the reconstruction synthesis route, at least for these specific monomers. Source data

Vacuum sealing procedures are often necessary for COF syntheses to prevent oxidation of the arylamine monomers under solvothermal conditions. Freeze-pump-thaw procedures can be performed in a research laboratory, but they could represent a major hurdle for industrial scale-up. By decorating with urea groups, we decrease the reactivity of the monomers and provide better oxidation resistance, and hence this reconstruction approach can be conducted without any vacuum degassing steps (Extended Data Fig. 8, Supplementary Information), while affording equivalent crystallinity and porosity (BET surface area *S*_BET_ = 1,650 m^2^ g^−1^ for RC-COF-1) relative to reactions that were performed with vacuum sealing (*S*_BET_ = 1,712 m^2^ g^–1^). Such simple vacuum-free, aqueous processes might prove decisive for the viable commercial scale-up of highly crystalline COFs, for which vacuum degassing and inertization are costly.

## Outlook

COFs can be highly crystalline or physicochemically robust, but rarely both^5^. On the basis of an unexpected framework reconstruction in urea COFs, we have established a general and scalable vacuum-free protocol to synthesize highly crystalline imine frameworks by using a reversible and removable urea linkage as a disposable tether to pre-organize monomers before irreversible polymerization. This separates the crystallization process from the formation of robust framework bonds. The removable covalent tether is stronger and more directional compared to the non-covalent interactions in strategies in which monomers are pre-organized in molecular crystals. The superior level of structural order in reconstructed COFs presents new opportunities for applications such as gas adsorption and photocatalysis. We used urea tethers here, but it is likely that other covalent pre-organization chemistries could be devised to access other frameworks.

## Methods

### Chemicals

All reagents were obtained from Sigma-Aldrich, Fisher Chemical, Adamas, Jilin Chinese Academy of Sciences–Yanshen Technology or Shanghai Tensus Bio-Chem Technology and used as received. Carbon nitride was bought from Carbodeon. Solvents were obtained from commercial sources and used without further purification. A sulfone-decorated imine COF, FS-COF, was synthesized according to previous literature^22^.

### Liquid NMR spectroscopy

^1^H and ^13^C NMR spectra were recorded in solution at 400 MHz and 100 MHz, respectively, using a Bruker Avance 400 NMR spectrometer.

### High-resolution mass spectrometry

The high-resolution mass spectrometry data were obtained using a Waters LCT Premier XE spectrometer.

### Powder X-ray diffraction

PXRD patterns were recorded on a Bruker D8 Advance diffractometer with Cu Kα radiation with a voltage of 40 kV. Data were collected in the 2*θ* range of 2–40° with steps of 0.02°.

### Fourier transform infrared spectroscopy

The FTIR spectra were recorded on neat samples in the range of 4,000–650 cm^−1^ on a PerkinElmer FTIR spectrometer equipped with a single reflection diamond ATR module.

### X-ray photoelectron spectroscopy

X-ray photoelectron spectroscopy (XPS) data were measured in powder form using an ESCALAB 250Xi instrument (Thermo Fisher Scientific) with a monochromatized Al K*α* line source.

### Elemental microanalyses

Elemental microanalyses were measured in the Research Center of Analysis and Test of East China University of Science and Technology using the EURO EA3000 Elemental Analyzer.

### Solid-state NMR spectroscopy

The solid-state ^13^C NMR spectra were recorded on a Bruker Avance 400 NMR spectrometer with CP-MAS at a ^13^C frequency of 100 MHz under 12 kHz spinning rate under MAS condition.

### Thermogravimetric analyses

Thermogravimetric analyses were performed on an EXSTAR6000 by heating samples at 20 °C min^−1^ under a nitrogen atmosphere to 800 °C.

### Gas adsorption analysis

Apparent surface areas were measured by nitrogen adsorption at 77.3 K using a Micromeritics ASAP 2020 volumetric adsorption analyser. Powder samples were degassed offline at 393 K for 12 h under a dynamic vacuum (10^−5^ bar). Before the adsorption test, the inert gas was removed using a high vacuum provided by the turbo molecular drag pump. The specific surface areas were evaluated using the BET model. Pore size distributions of COFs were obtained from fitting the nonlocal density functional theory to the adsorption data.

Low-pressure gas adsorption measurements of CO_2_ (273, 283, 293 and 308 K) were performed on MicrotacBELsorp Max and MaxII gas adsorption analysers. Ultrahigh-purity (higher than 99.999%) CO_2_ in compressed gas cylinders was used throughout all experiments. Samples were degassed at 393 K for 12 h before measurement. CO_2_ adsorption isotherms of each COF were then fitted with virial model equations as follows:

$$\mathrm{ln}(p)=\,\mathrm{ln}(N)+\frac{1}{T}{\sum }_{{\rm{i}}=0}^{m}{a}_{{\rm{i}}}\times {N}^{{\rm{i}}}+{\sum }_{{\rm{j}}=0}^{n}{b}_{{\rm{j}}}\times {N}^{{\rm{j}}},$$

in which *N* is the amount adsorbed (or uptake) in mmol g^−1^; *p* is the pressure in kPa; *T* is the temperature in K; and *m* and *n* are multinomial coefficients that determine the isosteric heat.

The isosteric heat of each COF was calculated from the virial fitting adsorption isotherms by using the Clausius–Clapeyron equation, in which *Q*_st_ is the isosteric heat in J mol^–1^, *T* is the temperature in K, *P* is the pressure in kPa, and *R* is the gas constant (8.314 J K^–1^ mol^–1^):

$$-{Q}_{{\rm{st}}}=R{T}^{2}{(\frac{\partial \mathrm{ln}P}{\partial T})}_{n}$$

### Scanning electron microscopy

COF morphologies were imaged using a field-emission scanning electron microscope (Helios G4 UC, Thermo Fisher Scientific).

### Transmission electron microscopy

Transmission electron microscopy (TEM) characterizations were performed on a Themis Z microscope (Thermo Fisher Scientific) equipped with two aberration correctors under 200 kV. To minimize the electron beam damage, a cryo-transfer TEM holder (Model 2550, Fischione Instruments) was used, and the temperature was set below −175 °C during TEM imaging.

### Ultraviolet-visible absorption spectroscopy

Ultraviolet (UV)-visible absorption spectra of the COFs were recorded on a PerkinElmer Lambda 950 UV-vis-NIR spectrometer by measuring the reflectance of powders in the solid state.

### Photoluminescence spectroscopy

Photoluminescence spectra were recorded on a Varian Cary Eclipse fluorescence spectrophotometer by measuring the powders in the solid state.

### Electron paramagnetic resonance spectroscopy

Electron paramagnetic resonance (EPR) spectra were acquired at room temperature under ambient conditions using a Bruker EMX-8/2.7 spectrometer. COF powders were taken in an EPR tube and excited with a 300-W Xe lamp using a 420-nm filter.

### Time-correlated single photon counting measurements

Time-correlated single photon counting measurements were performed on an Edinburgh Instruments LS980-D2S2-STM spectrometer equipped with picosecond-pulsed LED excitation sources and an R928 detector, with a stop count rate below 3%. An EPL-375 diode (*λ* = 370.5 nm, instrument response 100 ps, full width at half maximum, FWHM) with a 450-nm high-pass filter for emission detection was used. Suspensions were prepared by ultrasonicating the COF in water. The instrument response was measured with colloidal silica (LUDOX HS-40, Sigma-Aldrich) at the excitation wavelength without filter. Decay times were fitted in FAST software using suggested lifetime estimates.

### Photoelectrochemical measurements

Indium tin oxide (ITO) glasses were cleaned by sonication in ethanol and acetone for 30 min respectively, then dried under nitrogen flow. Two milligrams of COF was dispersed in 0.2 ml ethanol with µ110 ten  Nafion solution (5 wt% in a mixture of lower aliphatic alcohols and water) and ultrasonicated for 20 min to give a homogenous suspension. ITO glass slides were covered with a copper mask giving an area of 0.28 cm^2^. Ten microlitres of the suspension was drop-casted on the ITO glass and dried overnight at room temperature. Electrochemical impedance spectroscopy and photocurrent response were performed using a Bio-Logic SP-200 electrochemical system. A three-electrode set-up was used with a working electrode (COF on ITO glass), counter electrode (platinum plate) and reference electrode (Ag/AgCl), and the bias voltage was −0.35 V. A 300-W Newport Xe light source (model 6258, ozone-free) with a 420-nm filter was used to illuminate the samples. A solution of 0.5 M Na_2_SO_4_ (pH = 6.8) was used for measurement.

### Photocatalytic hydrogen evolution experiments

A quartz flask was charged with the photocatalyst powder (2.5 mg), 0.1 mol l^−1^ ascorbic acid water solution (25 ml) and a certain amount of platinum (Pt) as a cocatalyst, using hexachloroplatinic acid as a Pt precursor. The resulting suspension was ultrasonicated until the photocatalyst was well-dispersed before degassing by N_2_ bubbling for 30 min. The reaction mixture was illuminated with a 300 W Newport Xe light source (model 6258, ozone-free) using appropriate filters for the time specified under atmospheric pressure. The Xe light source was cooled by water circulating through a metal jacket. The samples were first illuminated for 5 h to complete Pt photo-deposition; then the flask was degassed by N_2_ bubbling for 30 min followed by the photocatalysis reaction. Gas samples were taken with a gas-tight syringe and run on a Bruker 450-GC gas chromatograph. Hydrogen was detected with a thermal conductivity detector referencing against standard gas with a known concentration of hydrogen. Hydrogen dissolved in the reaction mixture was not measured and the pressure increase generated by the evolved hydrogen was not considered in the calculations. The rates were determined from a linear regression fit. After 5 h of photocatalysis, no carbon monoxide associated with framework or ascorbic acid decomposition could be detected on a gas chromatography system equipped with a pulsed discharge detector.

For stability measurements, a flask was charged with 2.5 mg of COF photocatalyst, 0.1 mol l^−1^ ascorbic acid water solution (25 ml) and a certain amount of Pt (3 wt%) as a cocatalyst, using hexachloroplatinic acid as a Pt precursor. The resulting suspension was ultrasonicated to obtain a well-dispersed suspension, then transferred into a quartz reactor connected to a closed gas system (Labsolar-6A, Beijing Perfectlight). The reaction mixture was evacuated several times to ensure complete removal of oxygen, and the pressure was set to 13.33 kPa . The reactor was irradiated in a 90° angle with a 300-W Xe light-source. The wavelength of the incident light was controlled using a 420-nm long-pass cut-off filter. The temperature of the reaction solution was maintained at 10 °C by circulation of cool water. The evolved gases were detected on an online gas chromatograph (Shimadzu GC 2014C) with a thermal conductive detector. After the photocatalysis experiment, the photocatalyst was recovered by washing with water then solvent exchange with methanol and tetrahydrofuran, respectively, before drying at 60 °C under a vacuum.

### Measurement of external quantum efficiencies

The external quantum efficiencies (EQEs) for the photocatalytic H_2_ evolution were measured using monochromatic LED lamps (*λ* = 420, 490, 515 and 595 nm, respectively). For the experiments, the photocatalyst (2.5 mg) with Pt loading was suspended in an aqueous solution containing ascorbic acid (0.1 mol l^−1^). The light intensity was measured with a ThorLabs S120VC photodiode power sensor controlled by a ThorLabs PM100D Power and Energy Meter Console. The EQEs were estimated using the equation:

$${\rm{E}}{\rm{Q}}{\rm{E}}({\rm{ \% }})=\frac{2\times {{\rm{N}}{\rm{u}}{\rm{m}}{\rm{b}}{\rm{e}}{\rm{r}}{\rm{o}}{\rm{f}}{\rm{e}}{\rm{v}}{\rm{o}}{\rm{l}}{\rm{v}}{\rm{e}}{\rm{d}}{\rm{H}}}_{2}\,{\rm{m}}{\rm{o}}{\rm{l}}{\rm{e}}{\rm{c}}{\rm{u}}{\rm{l}}{\rm{e}}{\rm{s}}}{{\rm{N}}{\rm{u}}{\rm{m}}{\rm{b}}{\rm{e}}{\rm{r}}\,{\rm{o}}{\rm{f}}\,{\rm{i}}{\rm{n}}{\rm{c}}{\rm{i}}{\rm{d}}{\rm{e}}{\rm{n}}{\rm{t}}\,{\rm{p}}{\rm{h}}{\rm{o}}{\rm{t}}{\rm{o}}{\rm{n}}{\rm{s}}}\times 100{\rm{ \% }}$$

### Computational methods

Periodic DFT calculations were performed within the plane-wave pseudopotential formalism, using the Vienna ab initio simulation package (VASP) code^49^. The projector augmented-wave method was applied to describe the electron–ion interactions^50,51^. A kinetic-energy cut-off of 500 eV was used to define the plane-wave basis set, and the electronic Brillouin zone was integrated using Γ-centred Monkhorst−Pack grids with the smallest allowed spacing between *k*-points (KSPACING) being 0.25 Å^−1^. Geometry optimizations were performed using the Perdew−Burke−Ernzerhof exchange−correlation functional with the DFT-D3(BJ) dispersion correction^52–54^. Tolerances of 10^−6^ eV and 10^−2^ eV Å^−1^ were applied during the optimization of the Kohn−Sham wavefunctions and the geometry optimizations, respectively.

For crystal structures of COFs, both lattice parameters and atomic positions are allowed to change during geometry optimization. The electronic structures of the optimized RC-COF-1 and Urea-COF-1 structures were then computed using a screened hybrid exchange−correlation functional (HSE06), giving key electronic properties, such as band gap and electrostatic potential, for comparison of the COFs. Within periodic boundary conditions, the electronic eigenvalues are given with respect to an internal reference. To achieve valence band alignment, using a common vacuum level, so that band energies can be compared for the different COF structures, we followed an approach devised for determining the vacuum level of porous structures^55^.

For the binding model constructed for the hydrolysis products of the COF, the hydrolysis-released *p*-phenylenediamine monomer was assumed to be trapped in a three-layer COF model, with the first layer being decomposed. The periodic COF layer was parallel to the *XY* plane and separated from its periodic images along the *Z* direction by a vacuum of around 14 Å^−1^. The lattice parameters were fixed, and the atomic positions were fully optimized during this process.

The binding energy was computed using the following formula:

$$\varDelta {E}_{{\rm{bind}}}={E}_{{\rm{system}}}-{E}_{{\rm{monomer}}}-{E}_{{\rm{framework}}},$$

in which *E*_system_ is the energy of COF with the first layer hydrolysed, *E*_monomer_ and *E*_framework_ are the energies of *p*-phenylenediamine and framework, respectively, and the corresponding conformers were kept the same as in that system.

To visualize the intermolecular interactions between the *p*-phenylenediamine monomer and the COF fragment, we used the independent gradient model (IGM)^56^. The IGM method quantifies the net electron density gradient attenuation that is due to intermolecular interactions, identifying non-covalent interactions and generating data composed solely of intermolecular interactions for drawing the corresponding 3D isosurface representations. Here structures were extracted out from the periodic calculation result with no hydrogen atoms added to the fragment, because we used pro-molecular level electron density here. The Multiwfn program^57^ was used for IGM analyses and the VMD program^58^ was used for visualization.

The geometries of the complexes were fully optimized by means of the hybrid M06-2X functional^59^. For all atoms, the def2-SVP basis set^60,61^ was applied. No symmetry or geometry constraint was imposed during optimizations. The optimized geometries were verified as local minima on the potential energy surface by frequency computations at the same theoretical level. These calculations were performed with the Gaussian 16 suite of programs^62^. Water was used as the solvent in the SMD solvation model^63^. A temperature of 433 K was used for thermochemistry analysis in all calculations.

## Online content

Any methods, additional references, Nature Research reporting summaries, source data, extended data, supplementary information, acknowledgements, peer review information; details of author contributions and competing interests; and statements of data and code availability are available at 10.1038/s41586-022-04443-4.

## Supplementary information

Supplementary Information This file contains supplementary text, supplementary tables 1–5, supplementary figures 1–76 and supplementary references.

Supplementary Data This file contains coordinates files.

## Data Availability

The experimental and theoretical data that support the findings of this study are available from the corresponding authors upon reasonable request. Source data are provided with this paper.

## Source data

Source Data Fig. 2
Source Data Fig. 3
Source Data Extended Data Fig. 1
Source Data Extended Data Fig. 3
Source Data Extended Data Fig. 4
Source Data Extended Data Fig. 5
Source Data Extended Data Fig. 6
Source Data Extended Data Fig. 7
Source Data Extended Data Fig. 8
