# Supplementary material for: Reconstructed covalent organic frameworks
Source: Nature. 2022 Apr 6;604(7904):72–9. doi: 10.1038/s41586-022-04443-4 (PMC8986529; doi:10.1038/s41586-022-04443-4)
Supplement: Supplementary file 1 — This file contains supplementary text, supplementary tables 1–5, supplementary figures 1–76 and supplementary references. [file 41586_2022_4443_MOESM1_ESM.pdf]

nature portfolio

https://doi.org/10.1038/s41586-022-04443-4

Supplementary information

Reconstructed covalent organic frameworks

In the format provided by the authors and unedited

Supplementary information

Reconstructed Covalent Organic Frameworks

Weiwei Zhang[1], Linjiang Chen[1,2], Sheng Dai[1], Chengxi Zhao[1,2], Cheng Ma[3], Lei Wei[4], Minghui Zhu[3], Samantha Y. Chong[2], Haofan Yang[2], Lunjie Liu[2], Yang Bai[2], Miaojie Yu[1], Yongjie Xu[2], Xiao-Wei Zhu[2], Qiang Zhu[2], Shuhao An[1], Reiner Sebastian Sprick[2], Marc A. Little[2], Xiaofeng Wu[1,2], Shan Jiang[4], Yongzhen Wu[1], Yue-Biao Zhang[4], He Tian[1], Wei-Hong Zhu[1✉] & Andrew I. Cooper[1,2✉]

[1] Key Laboratory for Advanced Materials and Institute of Fine Chemicals, Joint International Research Laboratory of Precision Chemistry and Molecular Engineering, Feringa Nobel Prize Scientist Joint Research Center, Frontiers Science Center for Materiobiology and Dynamic Chemistry, School of Chemistry and Molecular Engineering, East China University of Science and Technology, Shanghai 200237, China.

[2] Leverhulme Centre for Functional Materials Design, Materials Innovation Factory and Department of Chemistry, University of Liverpool, Crown Street, Liverpool L69 7ZD, UK.

[3] State Key Laboratory of Chemical Engineering, East China University of Science and Technology, Shanghai 200237, China.

[4] School of Physical Science and Technology, ShanghaiTech University, Shanghai 201210, China.

✉e-mail: whzhu@ecust.edu.cn; aicooper@liverpool.ac.uk

Table of contents

Section 1 Synthetic procedures 3
Section 2 Evolution of PXRD patterns, FTIR spectra and chromatograms analysis 16
Section 3 X-ray photoelectron spectroscopy 18
Section 4 Breadth of the synthetic strategy 19
Section 5 Gas adsorption experiments 32
Section 6 Thermogravimetric analyses 36
Section 7 Scanning electron microscopy and transmission electron microscopy images 37
Section 8 Control experiments: hydrothermal treatment 40
Section 9 Characterizations of RC-COF-1 synthesized without vacuum degassing step 42
Section 10 Computational results 44
Section 11 Optical and electronic properties 45
Section 12 Photocatalytic hydrogen evolution experiments 50
Section 13 Post-photocatalysis characterization 54
Section 14 Comparison of hydrogen evolution rates (HERs) for different COFs 57
Section 15 Comparison of CO_2 uptake for different COFs 58
Section 16 Liquid NMR spectra 59
Section 17 HRMS spectra 66
References 70

Section 1 Synthetic procedures

Synthesis of 1,1'-(1,4-phenylene)diurea

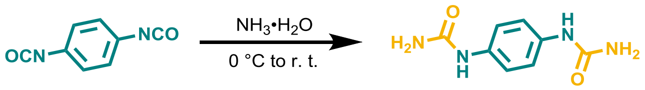

1,1'-(1,4-phenylene)diurea was synthesized according to previously reported procedures with some modifications[1]. To a solution of ammonium hydroxide (28% NH_3 in H_2O, 20 mL) cooled at 0 °C was added 1,4-phenylene diisocyanate (2.00 g, 12.49 mmol) portion-wise. The mixture was stirred at 0 °C for 30 min before being warmed to room temperature and further stirred overnight. The solid was collected by filtration, washed with water (100 mL) and tetrahydrofuran (THF, 100 mL) and then dried under vacuum to afford a white powder (2.27 g, yield 94%). ^{1}H NMR (400 MHz, DMSO-d_6): δ 8.31 (s, 2 H), 7.22 (s, 4 H), 5.72 (s, 4 H); ^{13}C NMR (100 MHz, DMSO-d_6): δ 156.06, 134.31, 118.38. *m/z* (ESI-HRMS) 217.0721 [M + Na]$^+$ (calcd. 217.0701).

Synthesis of 1,1'-(3,3'-dimethyl-[1,1'-biphenyl]-4,4'-diyl)diurea

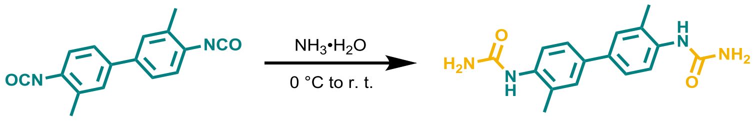

1,1'-(3,3'-Dimethyl-[1,1'-biphenyl]-4,4'-diyl)diurea was synthesized according to previously reported procedures with some modifications[1]. To a solution of ammonium hydroxide (28% NH_3 in H_2O, 20 mL) cooled at 0 °C was added 4,4'-diisocyanato-3,3'-dimethylbiphenyl (2.00 g, 7.57 mmol) portion-wise. The mixture was stirred at 0 °C for 30 min before being warmed to room temperature and further stirred overnight. The solid was collected by filtration, washed with water (100 mL) and THF (100 mL) and then dried under vacuum to afford a white powder (2.13 g, yield 94%). ^{1}H NMR (400 MHz, DMSO-d_6): δ 7.87 (d, J = 8.4 Hz, 2 H), 7.73 (s, 2 H), 7.39 (d, J = 1.7 Hz, 2 H), 7.35 (dd, J_1 = 8.4 Hz, J_2 = 1.9 Hz, 2 H), 6.06 (s, 4 H), 2.25 (s, 6 H); ^{13}C NMR (100 MHz, DMSO-d_6): δ 156.07, 137.13, 133.48, 127.63, 127.04, 123.67, 120.86, 18.08. *m/z* (ESI-HRMS) 297.1339 [M – H]$^-$ (calcd. 297.1352).

Synthesis of 1,1'-([1,1'-biphenyl]-4,4'-diyl)diurea

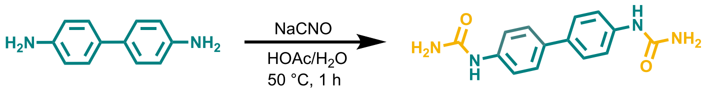

To a solution of benzidine (737.0 mg, 4.00 mmol) dissolved in acetic acid (10 mL) and water (5 mL), was added sodium cyanate (975.0 mg, 15.00 mmol in 10 mL warm water) at 50 °C with continuous stirring. The mixture was stirred at this temperature for 1 h. The resulting precipitation was collected by filtration, washed with water (50 mL) and THF (50 mL) and then dried under vacuum to afford a white powder (885.1 mg, yield 82%). ^{1}H NMR (400 MHz, DMSO-d_6): δ 8.58 (s, 2 H), 7.48 (d, J = 8.9 Hz, 4 H), 7.44 (d, J = 8.9 Hz, 4 H), 5.87 (s, 4 H); ^{13}C NMR (100 MHz, DMSO-d_6): δ 155.91, 139.36, 132.70, 126.11, 118.01. *m/z* (ESI-HRMS) 269.1024 [M – H]$^-$ (calcd. 269.1039).

Synthesis of 1,1'-(ethyne-1,2-diylbis(4,1-phenylene))diurea

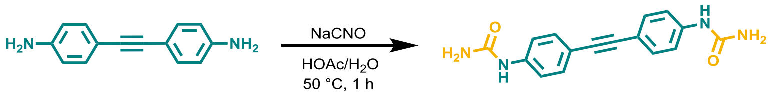

To a solution of 4,4'-(ethyne-1,2-diyl)dianiline (208.0 mg, 1.00 mmol) dissolved in acetic acid (20 mL) and water (2 mL), was added sodium cyanate (195.0 mg, 3.00 mmol in 2 mL warm water) at 50 °C with continuous stirring. The mixture was stirred at this temperature for 1 h. The resulting precipitation was collected by filtration, washed with water (50 mL), and then dried under vacuum to afford a light-yellow powder (189.2 mg, yield 64%). ^{1}H NMR (400 MHz, DMSO-d_6): δ 8.74 (s, 2 H), 7.43-7.37 (m, 8 H), 5.95 (s, 4 H); ^{13}C NMR (100 MHz, DMSO-d_6): δ 155.69, 140.76, 131.75, 117.46, 114.73, 88.31. *m/z* (ESI-HRMS) 293.1049 $[M – H]^{–}$ (calcd. 293.1039).

Synthesis of Urea-COF-1 (also known as COF-117)

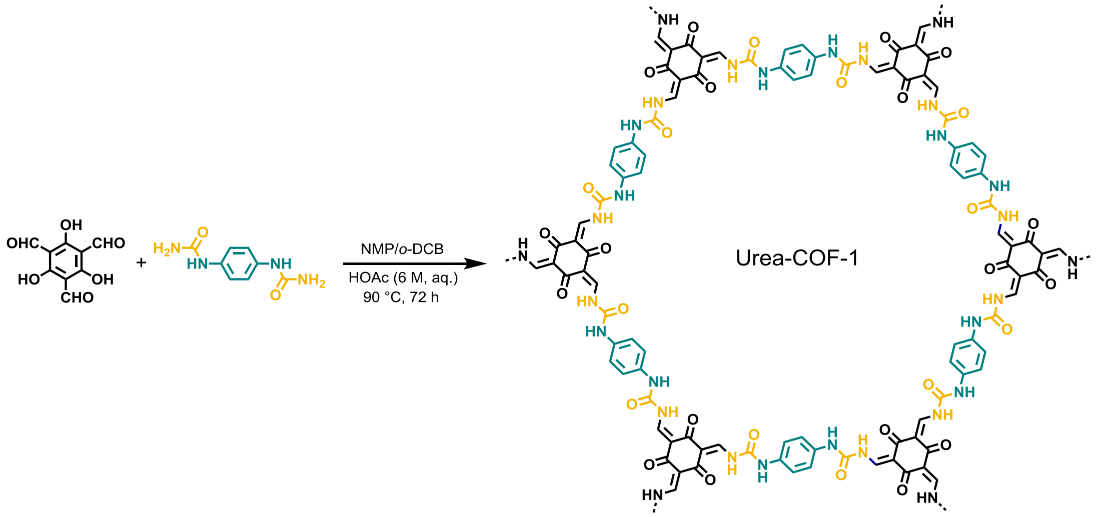

Urea-COF-1 was synthesized according to previously reported procedures with some modifications[1]. A Pyrex tube was charged with 1,3,5-triformylphloroglucinol (21.0 mg, 0.10 mmol), 1,1'-(1,4-phenylene)diurea (29.1 mg, 0.15 mmol), *N*-methyl-2-pyrrolidinone (NMP, 0.8 mL), 1,2-dichlorohenzene (*o*-DCB, 0.2 mL) and aqueous acetic acid (6 M, 0.1 mL). The mixture was briefly sonicated for 10 seconds, and the tube was then flash-frozen at 77.3 K (liquid nitrogen bath) and degassed by three freeze-pump-thaw cycles before being evacuated to an internal pressure of 100 mtorr. The tube was sealed and heated at 90 °C for 72 h. The obtained precipitate was isolated by filtration, briefly washed with *N*,*N*-dimethylformamide (DMF) and acetone to afford solvated COF. To activate the COF, solvent exchange was performed with DMF (20 mL × 12), methanol (20 mL × 6), THF (20 mL × 6) and hexane (20 mL × 6), respectively. The material was then dried under vacuum at 60 °C for 12 h to yield Urea-COF-1 as a yellow solid (37.2 mg, yield 83%). Elemental analysis of activated sample: Calcd. for $C_{42}H_{30}N_{12}O_{12}$: C: 56.38; H: 3.38; N: 18.79%; Found: C: 51.90; H: 4.76; N: 16.75%.

Chemical reconstruction (synthesis of RC-COF-1)

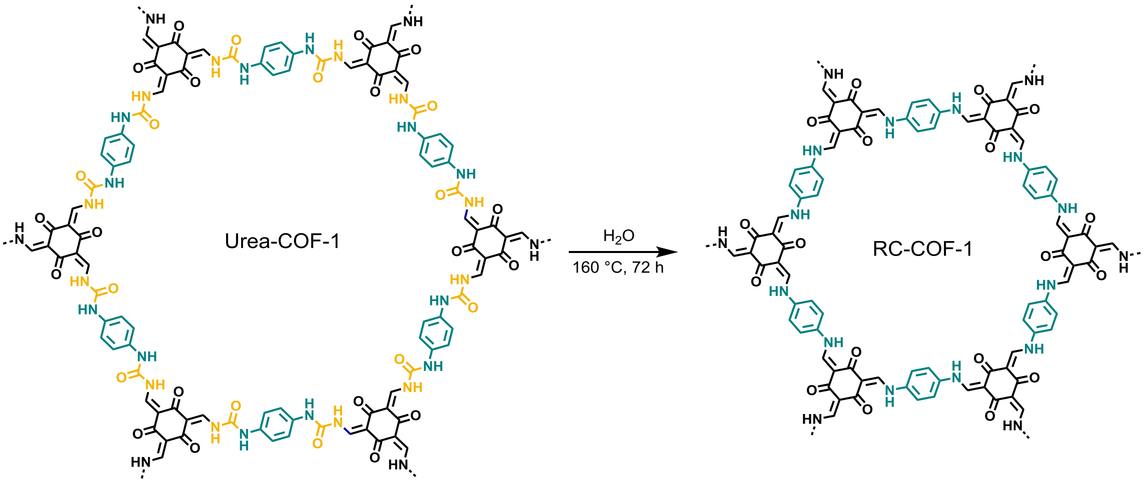

A Pyrex tube was charged with 1,3,5-triformylphloroglucinol (21.0 mg, 0.10 mmol), 1,1'-(1,4-phenylene)diurea (29.1 mg, 0.15 mmol), NMP (0.8 mL), *o*-DCB (0.2 mL) and aqueous acetic acid (6 M, 0.1 mL). The mixture was briefly sonicated for 10 seconds, and the tube was then flash-frozen at 77.3 K (liquid nitrogen bath) and degassed by three freeze-pump-thaw cycles before being evacuated to an internal pressure of 100 mtorr. The tube was sealed and heated at 90 °C for 72 h, and afterwards the temperatures was *in situ* elevated to 110, 120, 130, 150, 160 and 170 °C, respectively, and kept for a further 72 h. The obtained precipitates were isolated by filtration, briefly washed with DMF and acetone to afford solvated COFs. To activate the COFs, solvent exchange was performed with DMF (20 mL × 12), methanol (20 mL × 6), THF (20 mL × 6) and hexane (20 mL × 6), respectively. The material was then dried under vacuum at 60 °C for 12 h to yield the orange, red and dark red solids, respectively.

Based on the above results, we further explored the solvent effect on the transformation. In detail, Urea-COF-1 was isolated from reaction system (90 °C, 72 h) then briefly washed with DMF and acetone. Without vacuum dry, the powder (~ 37.2 mg) was then transfer into a Pyrex tube which was charged with solvent of *o*-DCB (1.0 mL), or NMP (1.0 mL), or glacial acetic acid (1.0 mL), or NMP/H_2O (9/1 *v*/*v*, 1.0 mL) or H_2O (1.0 mL). The mixture was sonicated for 2 min, and the tube was then flash-frozen at 77.3 K (liquid nitrogen bath) and degassed by three freeze-pump-thaw cycles before being evacuated to an internal pressure of 100 mtorr. The tube was sealed and heated at 160 °C for 72 h. **Caution:** gas could be released from reaction system. The precipitates were isolated by filtration, briefly washed with DMF and acetone to afford the solvated samples. To activate the samples, solvent exchange was performed with DMF (20 mL × 12), methanol (20 mL × 6), THF (20 mL × 6) and hexane (20 mL × 6), respectively. The materials were then dried under vacuum at 60 °C for 12 h to yield red and dark red solids, respectively. RC-COF-1 was synthesized by treating Urea-COF-1 with water at 160 °C for 72 h, which generated the product as a dark red solid (23.8 mg, yield 90%). The ~ 10% weight loss for isolation was mostly due to sample handling. Elemental analysis of activated sample: Calcd. for $C_{36}H_{24}N_6O_6$: C: 67.92; H: 3.80; N: 13.20%; Found: C: 62.26; H: 4.94; N: 11.89%.

Synthesis of DP-COF-1 (also known as TpPa-1)

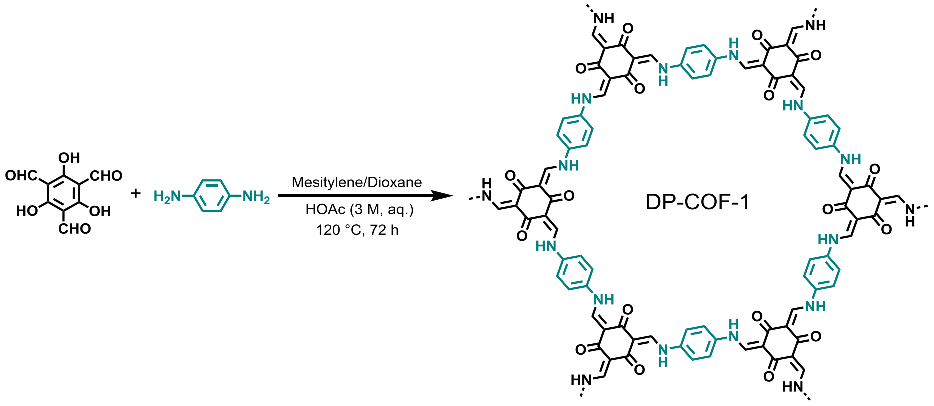

DP-COF-1 was synthesized using previously reported procedures[2]. A Pyrex tube was charged with 1,3,5-triformylphloroglucinol (63.0 mg, 0.30 mmol), benzene-1,4-diamine (48.0 mg, 0.45 mmol), mesitylene (1.5 mL), dioxane (1.5 mL) and 3 M aqueous acetic acid (0.5 mL). The mixture was sonicated for 5 min, and the tube was then flash-frozen at 77.3 K (liquid nitrogen bath) and degassed by three freeze-pump-thaw cycles before being evacuated to an internal pressure of 100 mtorr. The tube was sealed and heated at 120 °C for 72 h. The obtained precipitates were isolated by filtration. To activate the COFs, solvent exchange was performed with DMF (20 mL × 12), methanol (20 mL × 6), THF (20 mL × 6) and hexane (20 mL × 6), respectively. The material was then dried under vacuum at 60 °C for 12 h to yield a dark red solid (82.0 mg, yield 86%). Elemental analysis of activated sample: Calcd. for $C_{36}H_{24}N_6O_6$: C: 67.92; H: 3.80; N: 13.20%; Found: C: 62.04; H: 4.87; N: 11.92%.

Synthesis of Urea-COF-2 (also known as COF-118)

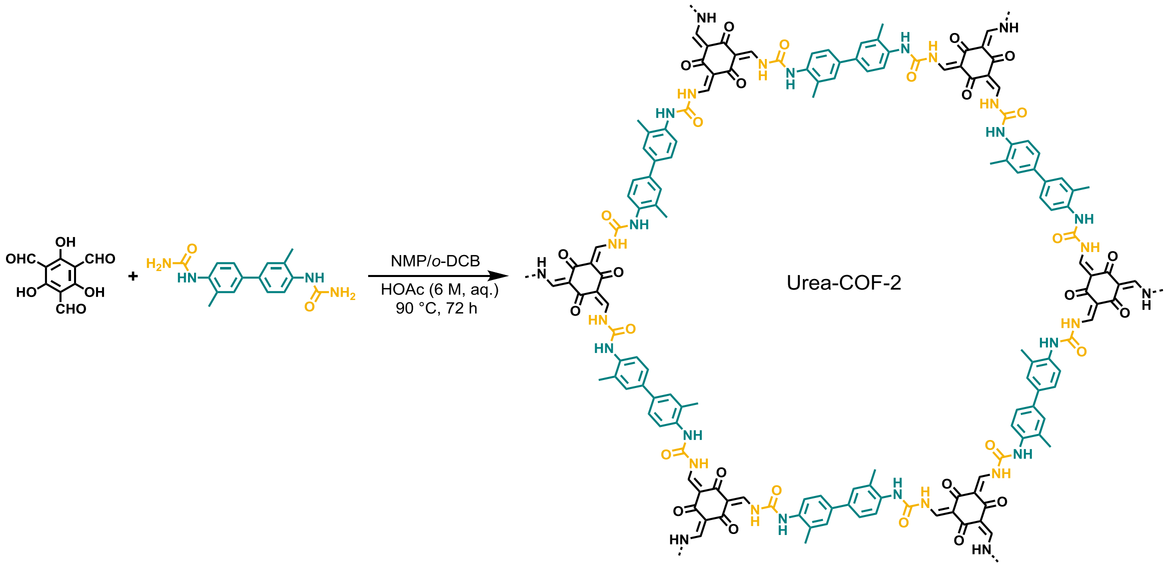

Urea-COF-2 was synthesized according to previously reported procedures with some modifications[1]. In a manner similar to the preparation of Urea-COF-1, treatment of 1,3,5-triformylphloroglucinol (21.0 mg, 0.10 mmol) and 1,1'-(3,3'-dimethyl-[1,1'-biphenyl]-4,4'-diyl)diurea (44.8 mg, 0.15 mmol) in a solution of NMP (1.2 mL), *o*-DCB (0.3 mL) and aqueous acetic acid (6 M, 0.1 mL) at 90 °C for 72 h yielded a brown precipitate (39.2 mg, yield 65%). Elemental analysis of activated sample: Calcd. for $C_{66}H_{54}N_{12}O_{12}$: C: 65.66; H: 4.51; N: 13.92%; Found: C: 63.28; H: 5.18; N: 12.43%.

Synthesis of RC-COF-2

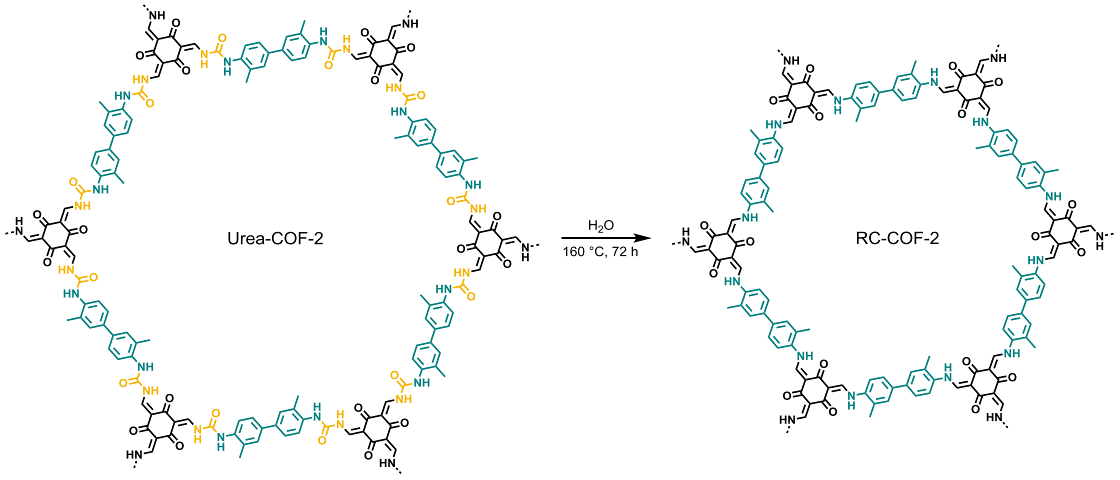

In a manner similar to the preparation of RC-COF-1, treatment of Urea-COF-2 with water at 160 °C for 72 h generated the product as a dark red solid (yield 89%). Elemental analysis of activated sample: Calcd. for $C_{51}H_{48}N_6O_6$: C: 72.84; H: 5.75; N: 9.99%; Found: C: 71.17; H: 5.43; N: 8.36%.

Synthesis of DP-COF-2 (also known as TpBD-Me$_2$)

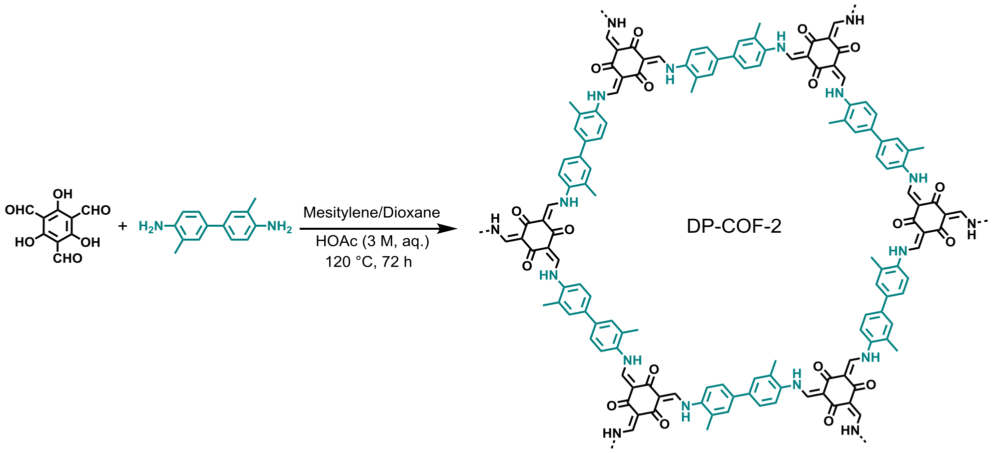

In a manner similar to the preparation of DP-COF-1 using previously reported procedures[3], treatment of 1,3,5-triformylphloroglucinol (63.0 mg, 0.30 mmol) and *o*-Tolidine (96.0 mg, 0.45 mmol) in a solution of mesitylene (1.5 mL), dioxane (1.5 mL) and aqueous acetic acid (3 M, 0.5 mL) at 120 °C for 72 h yielded a red precipitate (117.0 mg, yield 82%). Elemental analysis of activated sample: Calcd. for $C_{51}H_{48}N_6O_6$: C: 72.84; H: 5.75; N: 9.99%; Found: C: 72.36; H: 5.44; N: 8.41%.

Synthesis of Urea-COF-3

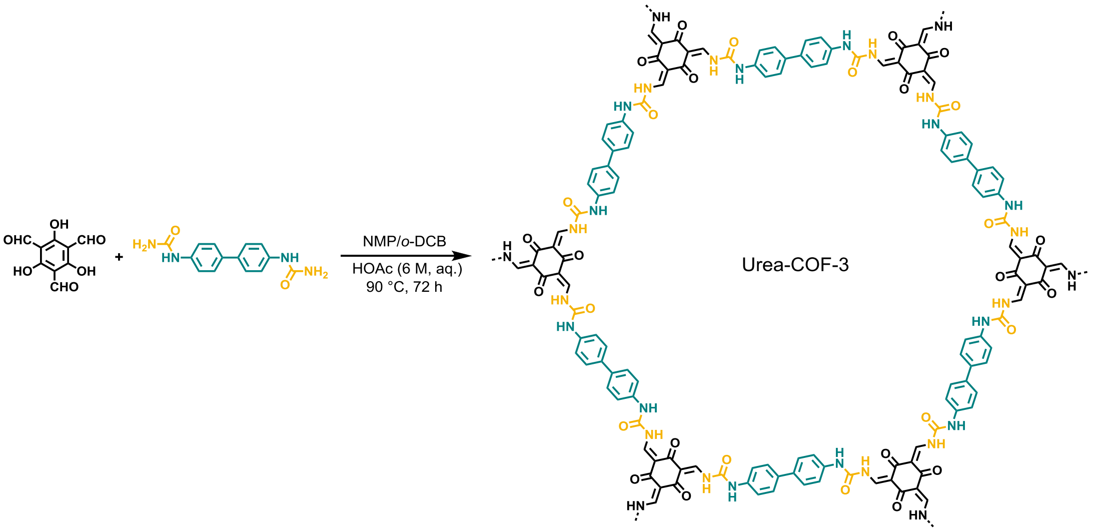

In a manner similar to the preparation of Urea-COF-1, treatment of 1,3,5-triformylphloroglucinol (21.0 mg, 0.10 mmol) and 1,1'-([1,1'-biphenyl]-4,4'-diyl)diurea (40.5 mg, 0.15 mmol) in a solution of NMP (0.8 mL), *o*-DCB (0.2 mL) and aqueous acetic acid (6 M, 0.1 mL) at 90 °C for 72 h yielded a brown precipitate (47.6 mg, yield 85%). Elemental analysis of activated sample: Calcd. for $C_{60}H_{42}N_{12}O_{12}$: C: 64.17; H: 3.77; N: 14.97%; Found: C: 62.93; H: 4.80; N: 14.14%.

Synthesis of RC-COF-3

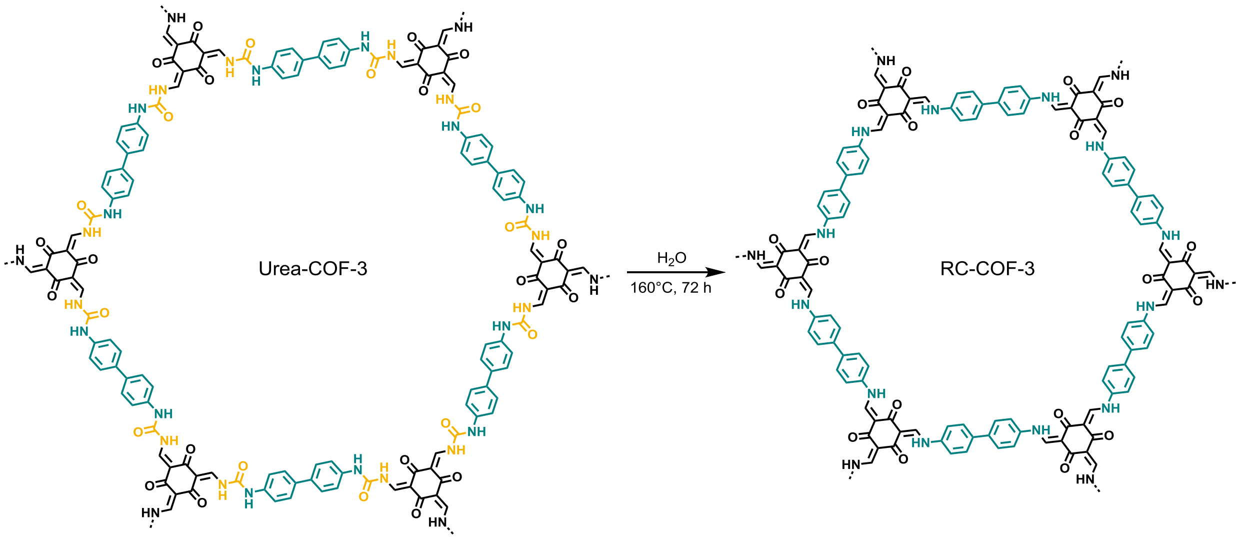

In a manner similar to the preparation of RC-COF-1, treatment of Urea-COF-3 with water at 160 °C for 72 h generated the product as a red solid (yield 90%). Elemental analysis of activated sample: Calcd. for $C_{54}H_{36}N_6O_6$: C: 74.99; H: 4.20; N: 9.72%; Found: C: 71.96; H: 4.78; N: 9.24%.

Synthesis of DP-COF-3 (also known as TpBD)

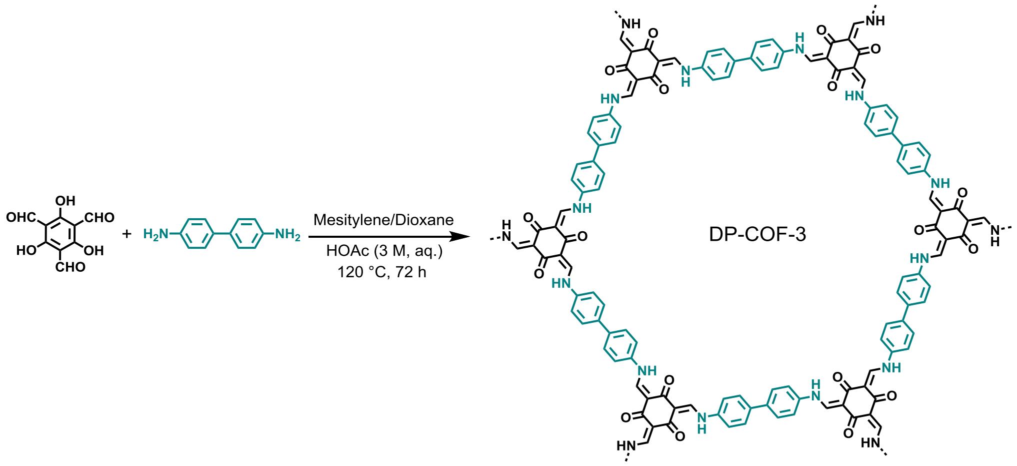

In a manner similar to the preparation of DP-COF-1 using previously reported procedures[4], treatment of 1,3,5-triformylphloroglucinol (63.0 mg, 0.30 mmol) and benzidine (83.0 mg, 0.45 mmol) in a solution of mesitylene (1.5 mL), dioxane (1.5 mL) and aqueous acetic acid (3 M, 0.5 mL) at 120 °C for 72 h yielded a red precipitate (109.2 mg, yield 84%). Elemental analysis of activated sample: Calcd. for $C_{54}H_{36}N_6O_6$: C: 74.99; H: 4.20; N: 9.72%; Found: C: 70.14; H: 5.03; N: 8.60%.

Synthesis of Urea-COF-4

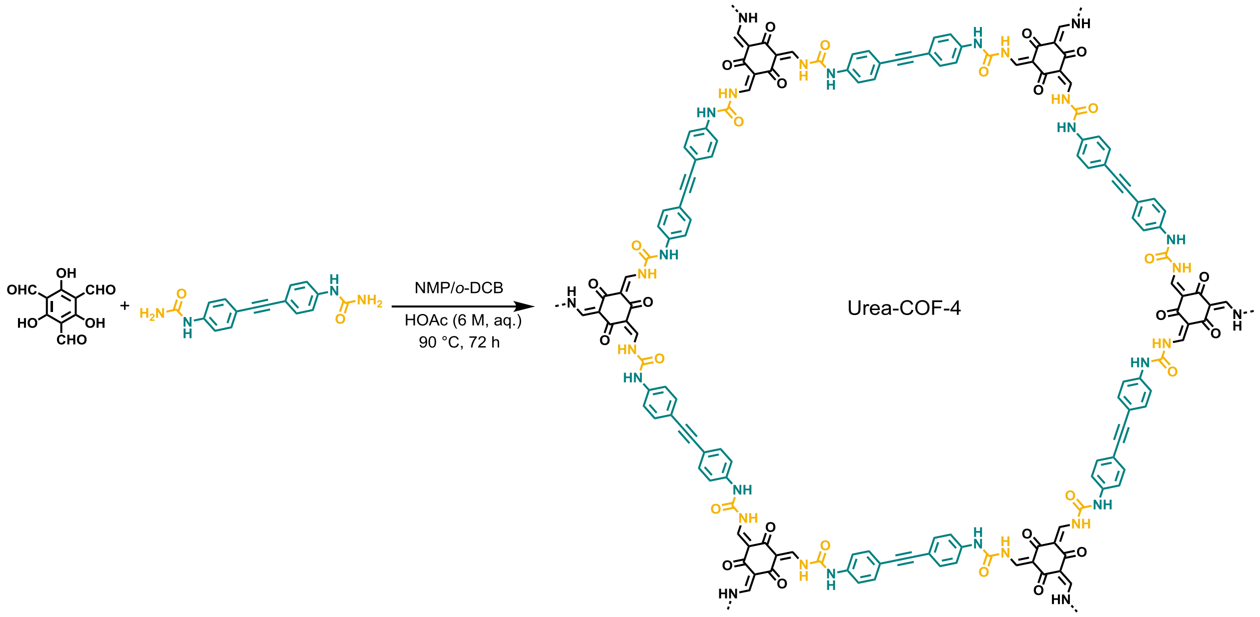

In a manner similar to the preparation of Urea-COF-1, treatment of 1,3,5-triformylphloroglucinol (21.0 mg, 0.10 mmol) and 1,1'-(ethyne-1,2-diylbis(4,1-phenylene))diurea (44.1 mg, 0.15 mmol) in a solution of NMP (1.2 mL), *o*-DCB (0.3 mL) and aqueous acetic acid (6 M, 0.1 mL) at 90 °C for 72 h yielded a brown precipitate (48.5 mg, yield 81%). Elemental analysis of activated sample: Calcd. for $C_{66}H_{42}N_{12}O_{12}$: C: 66.33; H: 3.54; N: 14.06%; Found: C: 63.61; H: 4.49; N: 13.00%.

Synthesis of RC-COF-4

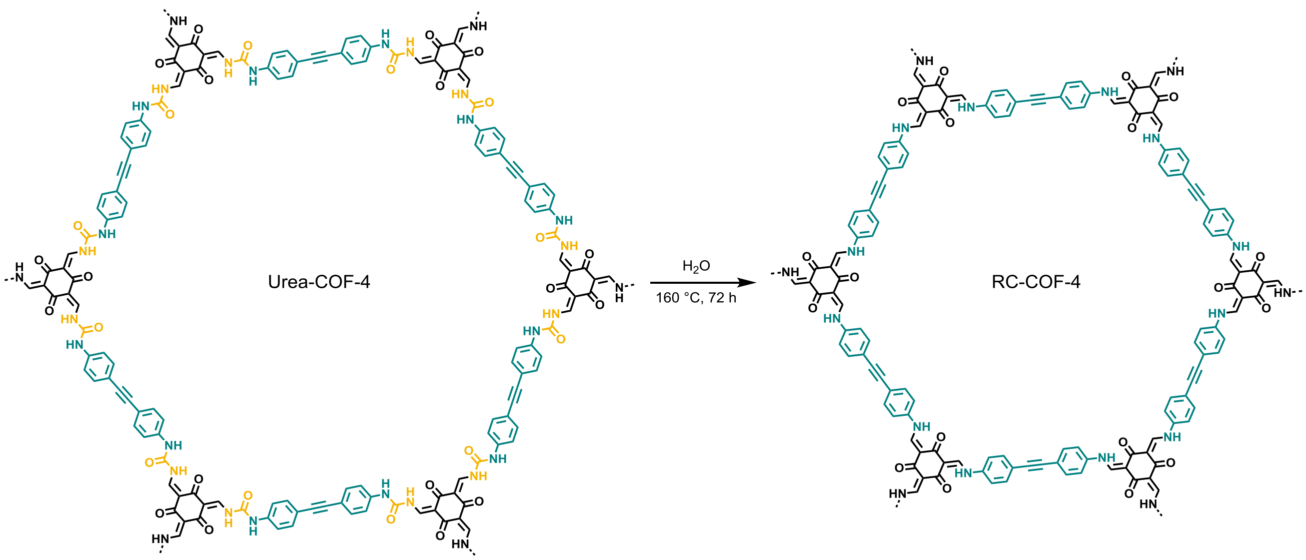

In a manner similar to the preparation of RC-COF-1, treatment of Urea-COF-4 with water at 160 °C for 72 h generated the product as a red solid (yield 91%). Elemental analysis of activated sample: Calcd. for $C_{60}H_{36}N_6O_6$: C: 76.91; H: 3.87; N: 8.97%; Found: C: 68.96; H: 4.60; N: 7.94%.

Synthesis of DP-COF-4 (also known as TP-EDDA)

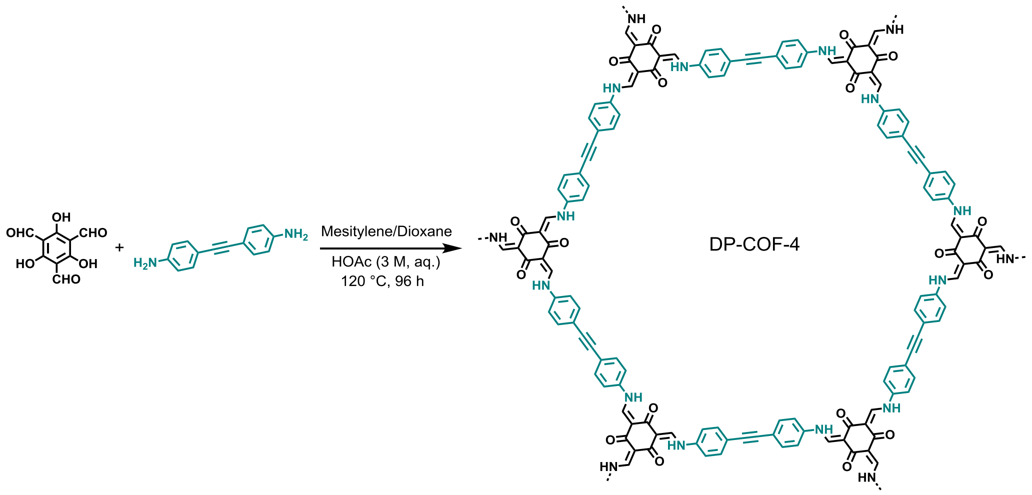

In a manner similar to the preparation of DP-COF-1 using previously reported procedures[5], treatment of 1,3,5-triformylphloroglucinol (63.0 mg, 0.30 mmol) and 4,4'-(ethyne-1,2-diyl)dianiline (94.0 mg, 0.45 mmol) in a solution of mesitylene (1.5 mL), dioxane (1.5 mL) and aqueous acetic acid (3 M, 0.5 mL) at 120 °C for 96 h yielded a red precipitate (127.1 mg, yield 90%). Elemental analysis of activated sample: Calcd. for $C_{60}H_{36}N_6O_6$: C: 76.91; H: 3.87; N: 8.97%; Found: C: 71.60; H: 4.66; N: 7.91%.

Supplementary Table 1 Elemental analyses for COFs.

COF	C wt.%	H wt.%	N wt.%
Urea-COF-1	51.90 (56.38)	4.76 (3.38)	16.75 (18.79)
RC-COF-1	62.26 (67.92)	4.94 (3.80)	11.89 (13.20)
DP-COF-1	62.04 (67.92)	4.87 (3.80)	11.92 (13.20)
Urea-COF-2	63.28 (65.66)	5.18 (4.51)	12.43 (13.92)
RC-COF-2	71.17 (72.84)	5.43 (5.75)	8.36 (9.99)
DP-COF-2	72.36 (72.84)	5.44 (5.75)	8.41 (9.99)
Urea-COF-3	62.93 (64.17)	4.80 (3.77)	14.14 (14.97)
RC-COF-3	71.96 (74.99)	4.78 (4.20)	9.24 (9.72)
DP-COF-3	70.14 (74.99)	5.03 (4.20)	8.60 (9.72)
Urea-COF-4	63.61 (66.33)	4.49 (3.54)	13.00 (14.06)
RC-COF-4	68.96 (76.91)	4.60 (3.87)	7.94 (8.97)
DP-COF-4	71.60 (76.91)	4.66 (3.87)	7.91 (8.97)

The table gives the experimental values and the calculated, theoretical elemental compositions (in parentheses). The theoretical values assume (a) perfect, infinite crystals (no surface end groups) and (b) no adsorption of guests, such as water vapour, in the pores, neither of which are the case (see *e.g.*, Supplementary Fig. 34, 35).

Synthesis of Urea-COF-1 and RC-COF-1 without vacuum degassing step

A Pyrex tube was charged with 1,3,5-triformylphloroglucinol (21.0 mg, 0.10 mmol), 1,1'-(1,4-phenylene)diurea (29.1 mg, 0.15 mmol), NMP (0.8 mL), *o*-DCB (0.2 mL) and aqueous acetic acid (6 M, 0.1 mL). The mixture was briefly sonicated for 10 seconds, and the tube was sealed in the air and heated at 90 °C for 72 h. The obtained precipitate was isolated by filtration, briefly washed with DMF and acetone. To activate the COFs, solvent exchange was performed with DMF (20 mL × 12), methanol (20 mL × 6), THF (20 mL × 6) and hexane (20 mL × 6), respectively. The material was then dried under vacuum at 60 °C for 12 h to yield Urea-COF-1 as a yellow solid (38.5 mg, yield 86%).

For RC-COF-1, Urea-COF-1 was isolated by filtration, briefly washed with DMF and acetone. The powder was then transfer into a Pyrex tube which was charged with deionized water (1.0 mL). The mixture was sonicated for 2 min, and the tube was then sealed in the air and heated at 160 °C for 72 h. The resulted powder was isolated by filtration, briefly washed with DMF and acetone. To activate the samples, solvent exchange was performed with DMF (20 mL × 12), methanol (20 mL × 6), THF (20 mL × 6) and hexane (20 mL × 6), respectively. The material was then dried under vacuum at 60 °C for 12 h to give a dark red solid (24.9 mg, yield 91%).

Synthesis of (2,4,6-trioxocyclohexane-1,3,5-triylidene)tris(methanylylidene)tris(3-phenylurea)

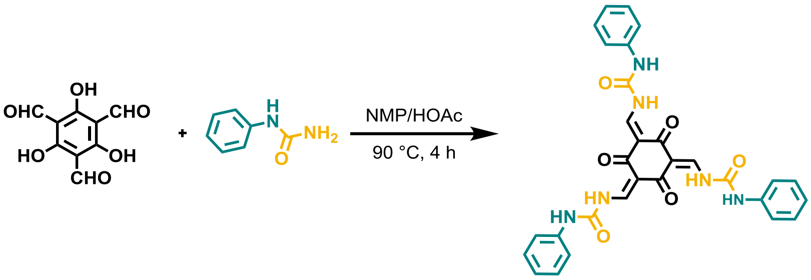

(2,4,6-trioxocyclohexane-1,3,5-triylidene)tris(methanylylidene)tris(3-phenylurea) was synthesized according to previously reported procedures with some modifications[1]. A 25 mL flask was charged with 1,3,5-triformylphloroglucinol (63.0 mg, 0.30 mmol), phenylurea (125.0 mg, 0.92 mmol), NMP (3 mL) and glacial acetic acid (0.2 mL). The reaction mixture was heated to 90 °C and stirred for 4 h before being cooled to room temperature. The mixture was then poured into water (100 mL) and the resulting precipitate was collected via filtration, washed with water (50 mL), methanol (50 mL) and dried under vacuum at room temperature to afford the urea-linked model compound as a yellow solid (102.0 mg, yield 60%). ^{1}H NMR (400 MHz, DMSO-d_6): δ 12.34 (d, J = 12.4 Hz, 3 H), 10.74 (s, 3 H), 8.77 (d, J = 12.4 Hz, 3 H), 7.56 (d, J = 7.8 Hz, 6 H), 7.36 (m, 6 H), 7.10 (m, 3 H); ^{13}C NMR (100 MHz, DMSO-d_6): δ 184.68, 149.78, 147.96, 138.07, 128.99, 123.53, 118.91, 108.68. *m/z* (ESI-HRMS) 563.1688 [M – H]$^-$ (calcd. 563.1679).

Synthesis of 2,4,6-tris((phenylamino)methylene) cyclohexane-1,3,5-trione

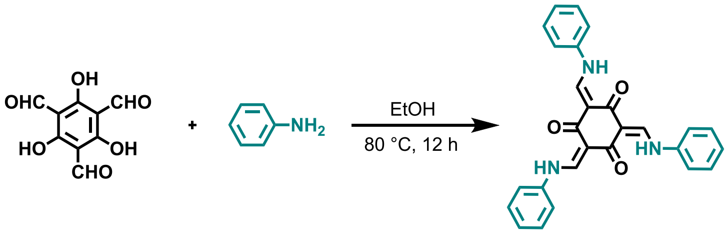

2,4,6-tris((phenylamino)methylene) cyclohexane-1,3,5-trione was synthesized according to previously reported procedures[2]. A 25 mL flask was charged with 1,3,5-triformylphloroglucinol (84.0 mg, 0.40 mmol), aniline (250.0 mg, 2.70 mmol), ethanol (40 mL). The reaction mixture was heated to 80 °C and stirred for 12 h before being cooled to room temperature. The resulting precipitate was collected via filtration, washed with ethanol (50 mL), and dried under vacuum at room temperature to afford the β-ketoenamine model compound as a yellow solid (126.1 mg, yield 72%). ^{1}H NMR (400 MHz, $CDCl_3$): δ 13.40 (d, J = 12.9 Hz, 3 H), 8.79 (d, J = 13.0 Hz, 3 H), 7.44-7.41 (m, 6 H), 7.32-7.30 (m, 6 H), 7.24-7.20 (m, 3 H); ^{13}C NMR (100 MHz, $CDCl_3$): δ 185.56, 149.35, 139.08, 129.94, 125.71, 117.71, 106.71. *m/z* (ESI-HRMS) 434.1502 [M – H]$^-$ (calcd. 434.1505).

Transformation of the model compound

To further understand this unexpected crystal-to-crystal transformation, we tested a water-insoluble urea model compound (Fig. 1b) under the same conditions as for the Urea-COF (H_2O, 160 °C). A *β*-ketoenamine product was obtained, but the isolated yield was much lower (~ 11%). By contrast, decomposition occurred when the model compound was dissolved in an NMP/H_2O mixture (9/1 *v*/*v*) at 160 °C, as determined by NMR (Supplementary Fig. 1, 2). These results suggest that the nanoconfinement effect and perhaps the porosity and 2D layered structure in the COFs are necessary for the reconstruction reaction, noting that the urea model compound is not porous.

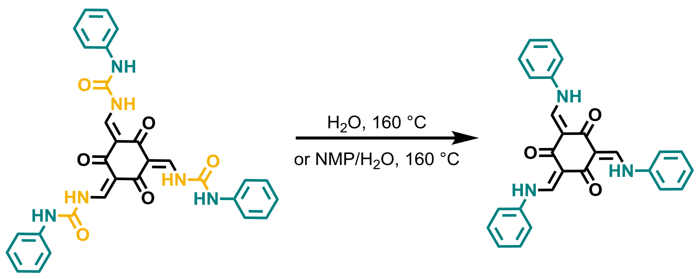

Transformation in solid state (H_2O): A Pyrex tube was charged with (2,4,6-trioxocyclohexane-1,3,5-triylidene)tris(methanylylidene)tris(3-phenylurea) (200.0 mg, 0.35 mmol) and water (1.0 mL). The mixture was sonicated for 2 min, and the tube was then flash-frozen at 77.3 K (liquid nitrogen bath) and degassed by three freeze-pump-thaw cycles before being evacuated to an internal pressure of 100 mtorr. The tube was sealed and heated at 160 °C for 72 h. The precipitate was isolated by filtration and washed with water (50 mL) to afford 2,4,6-tris((phenylamino)methylene) cyclohexane-1,3,5-trione (17.1 mg, yield 11%). ^{1}H NMR (400 MHz, $CDCl_3$): δ 13.41 (d, J = 13.5 Hz, 3 H), 8.79 (d, J = 13.1 Hz, 3 H), 7.43-7.41 (m, 6 H), 7.32-7.30 (m, 6 H), 7.24-7.20 (m, 3 H); ^{13}C NMR (100 MHz, $CDCl_3$): δ 185.56, 149.38, 139.08, 129.94, 125.71, 117.73, 106.71. *m/z* (ESI-HRMS) 434.1515 $[M – H]^-$ (calcd. 434.1505).

Transformation in solution state (NMP/H_2O): A Pyrex tube was charged with (2,4,6-trioxocyclohexane-1,3,5-triylidene)tris(methanylylidene)tris(3-phenylurea) (100.0 mg, 0.18 mmol), NMP (1.8 mL) and water (0.2 mL). The mixture was sonicated for 2 min, and the tube was then flash-frozen at 77.3 K (liquid nitrogen bath) and degassed by three freeze-pump-thaw cycles before being evacuated to an internal pressure of 100 mtorr. The tube was sealed and heated at 160 °C for 12 h or 72 h before being cooled to room temperature. The reaction mixture was mixed with water (30 mL). The precipitate was isolated by filtration and washed with water (50 mL). *m/z* (ESI-HRMS) not found $[M – H]^-$ (calcd. 434.1505); not found $[M + H]^+$ (calcd. 436.1651).

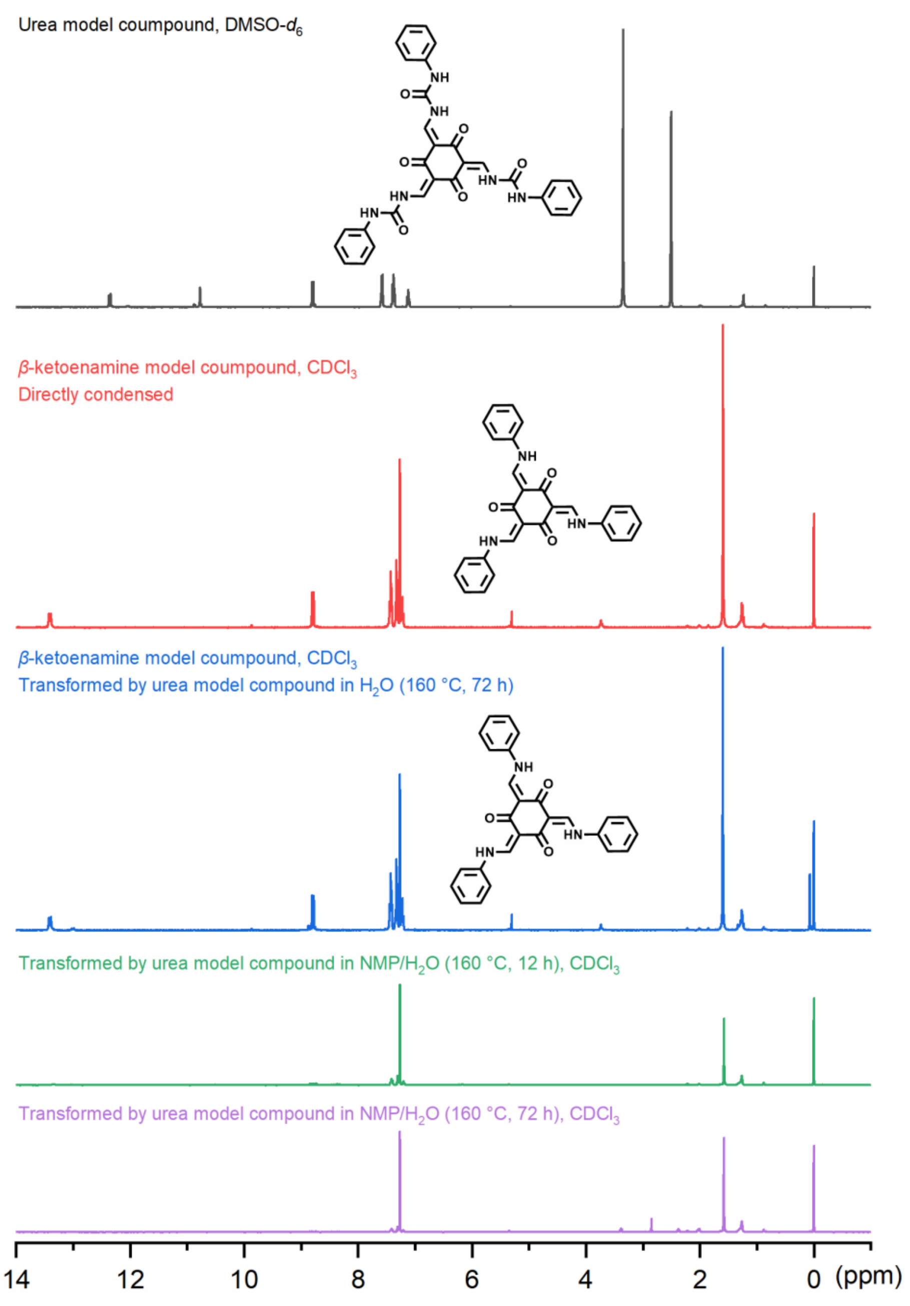

Supplementary Fig. 1 ^{1}H NMR spectrum for urea model compound (black) and *β*-ketoenamine model compound (red) synthesized by direct condensation, and *β*-ketoenamine model compound synthesized by transforming urea model compound in water (160 °C, 72 h, blue), and the product of transforming urea model compound in a mixture of NMP and water (NMP/H_2O = 9/1 *v*/*v*, 160 °C, 12 or 72 h, green or violet). No *β*-ketoenamine model product was detected in the last two cases by ^{1}H NMR.

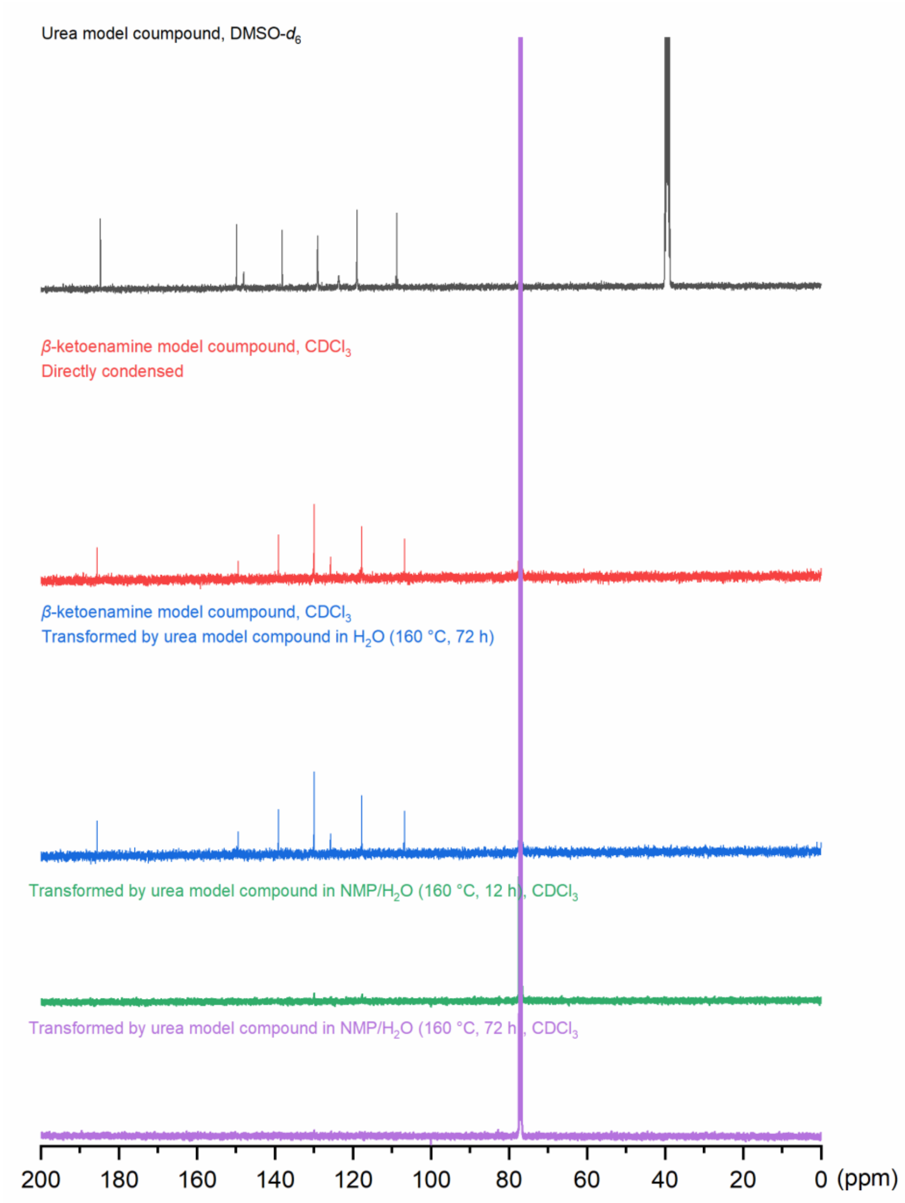

Supplementary Fig. 2 ^{13}C NMR spectrum for urea model compound (black) and *β*-ketoenamine model compound (red) synthesized by direct condensation, and *β*-ketoenamine model compound synthesized by transforming urea model compound in water (160 °C, 72 h, blue), and the product of transforming urea model compound in a mixture of NMP and water (NMP/H_2O = 9/1 *v*/*v*, 160 °C, 12 or 72 h, green or violet). Also, no *β*-ketoenamine model product was detected in the last two cases in the ^{13}C NMR spectrum.

Section 2 Evolution of PXRD patterns, FTIR spectra and chromatograms analysis

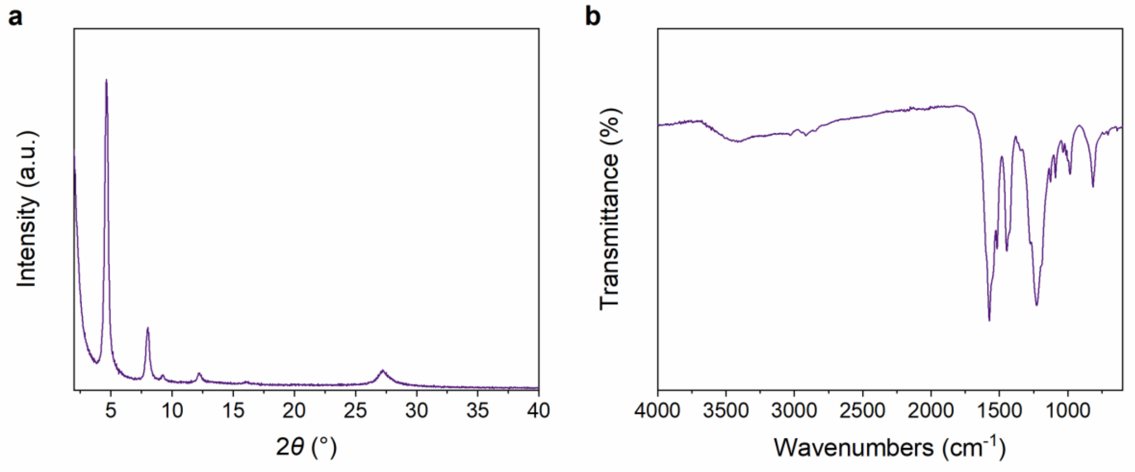

Supplementary Fig. 3 PXRD pattern (**a**) and FTIR spectrum (**b**) for as-synthesized Urea-COF-1 under an *in situ* elevated reaction temperature of 170 °C for a further 72 hours.

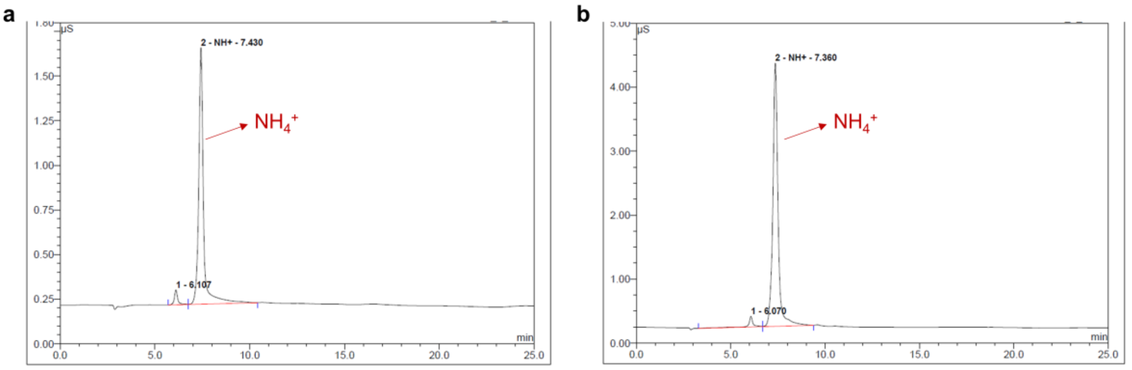

(a)

No.	Ret.Time min	Peak Name	Height µS	Area µS*min	Rel.Area %	Amount	Type
1	6.11	n.a.	0.084	0.018	3.98	n.a.	BM
2	7.43	NH+	1.438	0.424	96.02	1.013	MB
Total:			1.521	0.441	100.00	1.013	

(b)

No.	Ret.Time min	Peak Name	Height µS	Area µS*min	Rel.Area %	Amount	Type
1	6.07	n.a.	0.167	0.047	3.48	n.a.	BM
2	7.36	NH+	4.115	1.317	96.52	1573.840	MB
Total:			4.282	1.364	100.00	1573.840	

Supplementary Fig. 4 Ion chromatograms reports for standard ammonium ion solution (**a**) and aqueous solution that was filtered from reaction system (hydrothermal treatment of Urea-COF-1 at 160 °C for 72 hours, **b**). The experiments were conducted with ICS-3000 system (Thermo Fisher Scientific, USA) equipped with a conductivity detector. 30 mM methanesulfonic acid (MSA) was used as eluent and the column temperature was maintained at 40 °C with a run time of 20 min. The aqueous solution filtered from reaction system was diluted by 500 times. A high concentration (1.574 mg L^{-1}) of ammonium ion was detected, demonstrating the release of ammonia as a by-product during hydrothermal treatment.

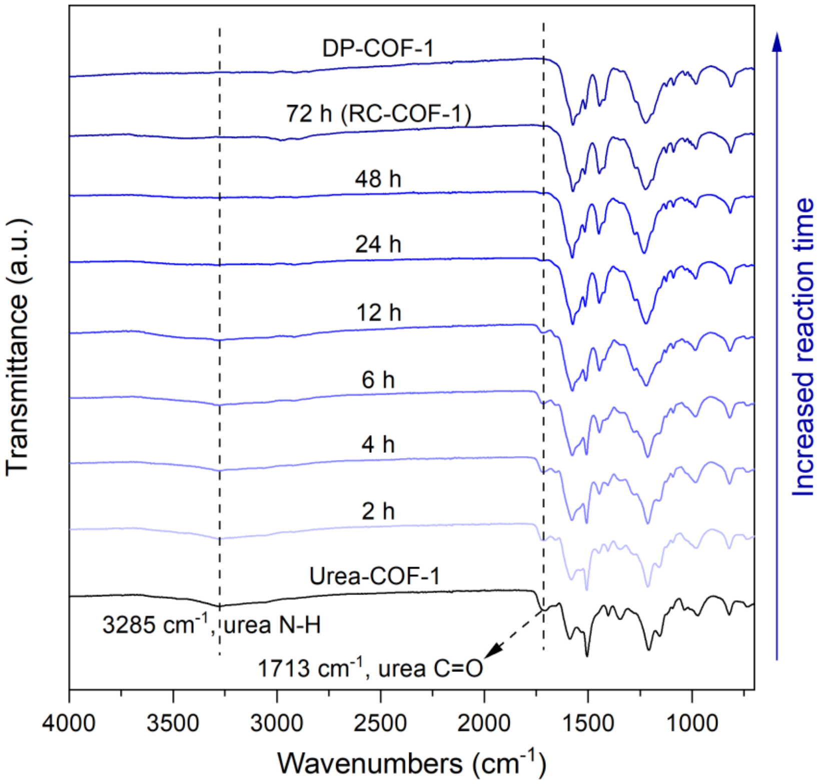

Supplementary Fig. 5 The evolution of the FTIR spectra with time for as-synthesized Urea-COF-1 by treatment with water at the elevated reaction temperatures of 160 °C (samples were measured after activation).

Section 3 X-ray photoelectron spectroscopy

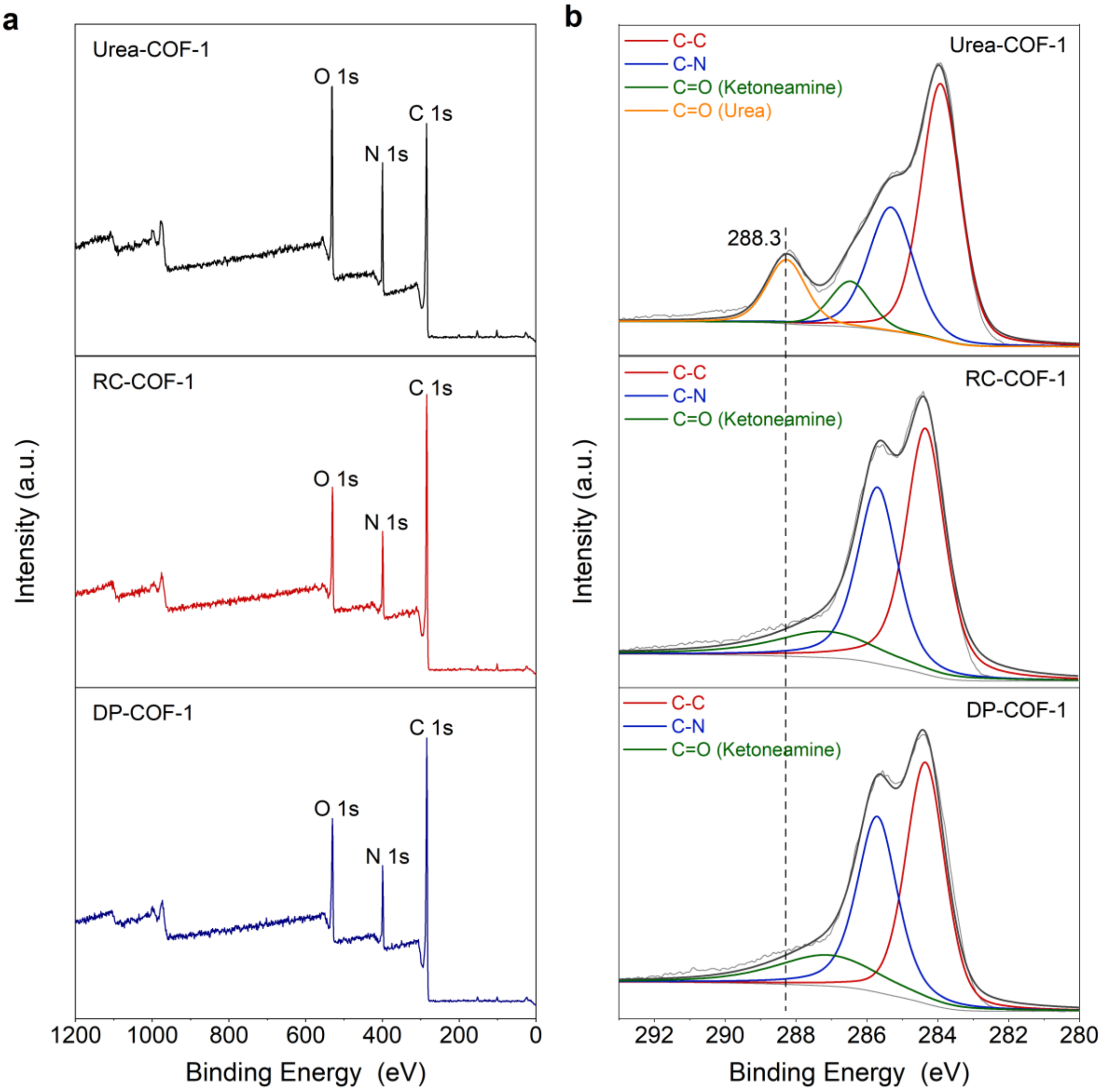

Supplementary Fig. 6 XPS survey spectra of Urea-COF-1, RC-COF-1 and DP-COF-1 (**a**). High-resolution XPS spectra of C 1s for Urea-COF-1, RC-COF-1 and DP-COF-1, showing the absence of urea C=O after chemical reconstruction (**b**). These data further support the solvothermal transformation of Urea-COF-1 to the *β*-ketoenamine COF, RC-COF-1, in the presence of water.

Section 4 Breadth of the synthetic strategy

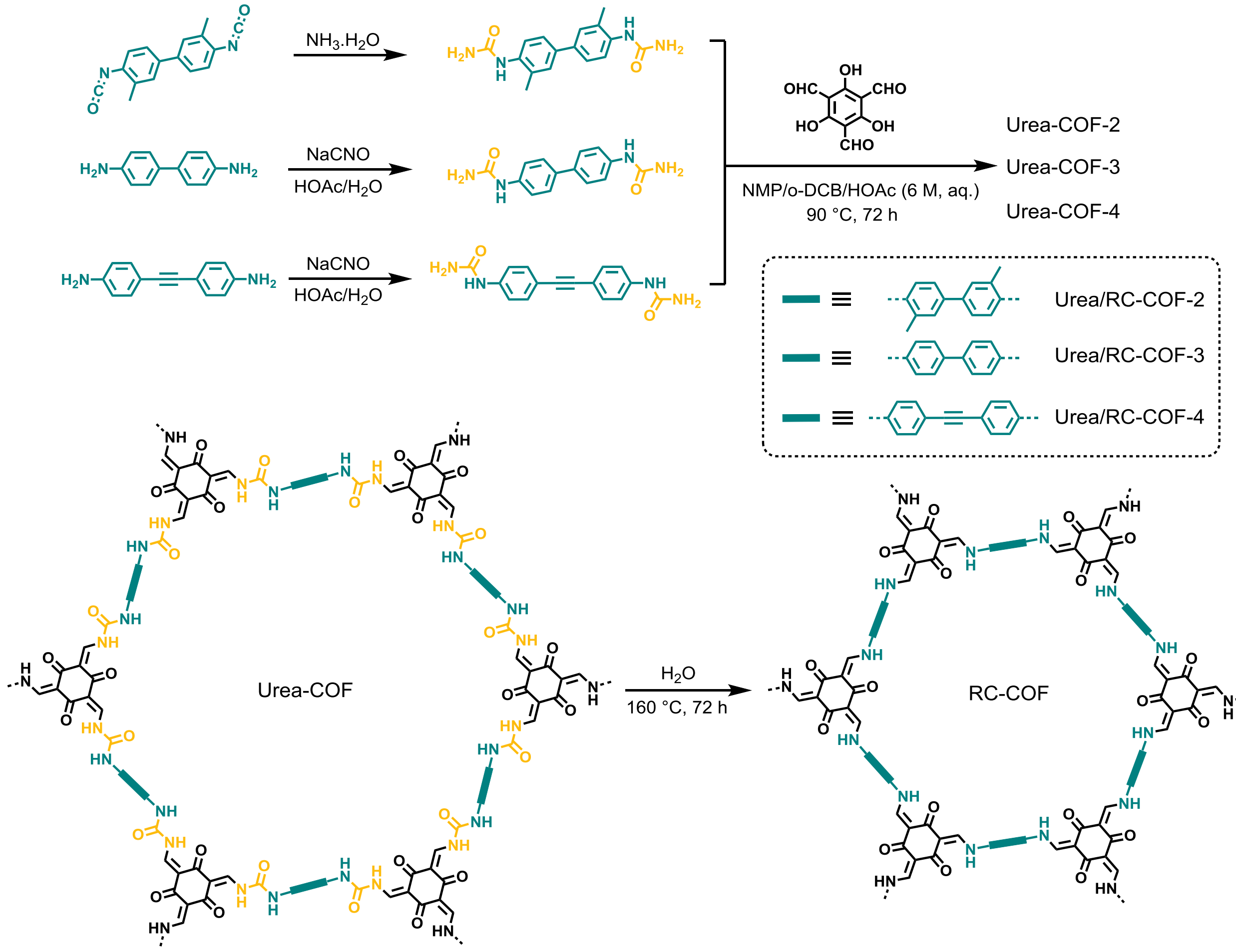

Supplementary Fig. 7 Synthetic schemes for RC-COF-2, RC-COF-3, RC-COF-4 reconstructed from the respective Urea-COFs**.** Besides isocyanates (Urea-COF-2), COFs can also be constructed from arylamine starting materials (Urea-COF-3 and Urea-COF-4), significantly expanding the potential synthetic scope. All urea-COFs can reconstruct into RC-COFs by treatment with water at 160 °C for 72 hours, which represents a generalizable synthetic route for preparing high-crystallinity imine COFs using readily accessible monomers.

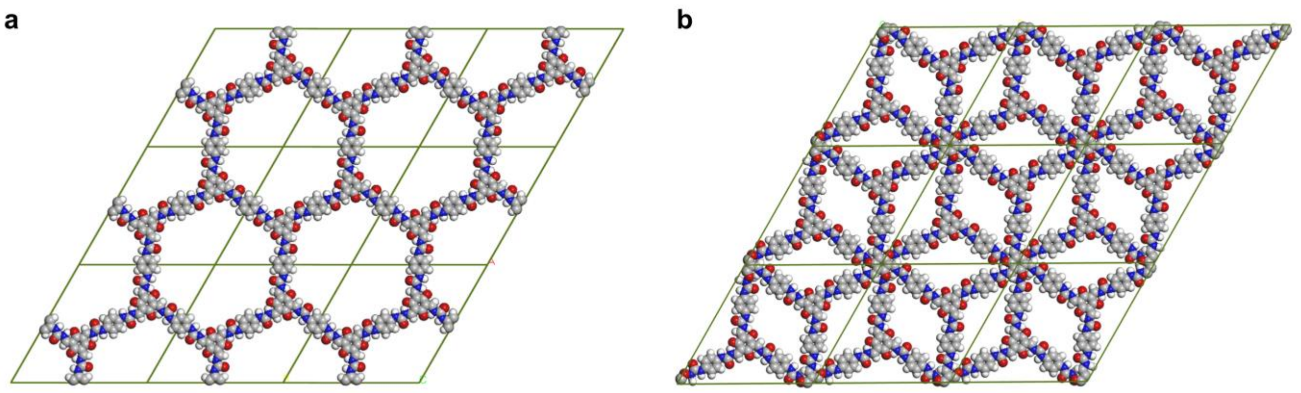

Supplementary Fig. 8 The top view of eclipsed AA-stacking (**a**) and staggered AB-stacking (**b**) models for Urea-COF-1.

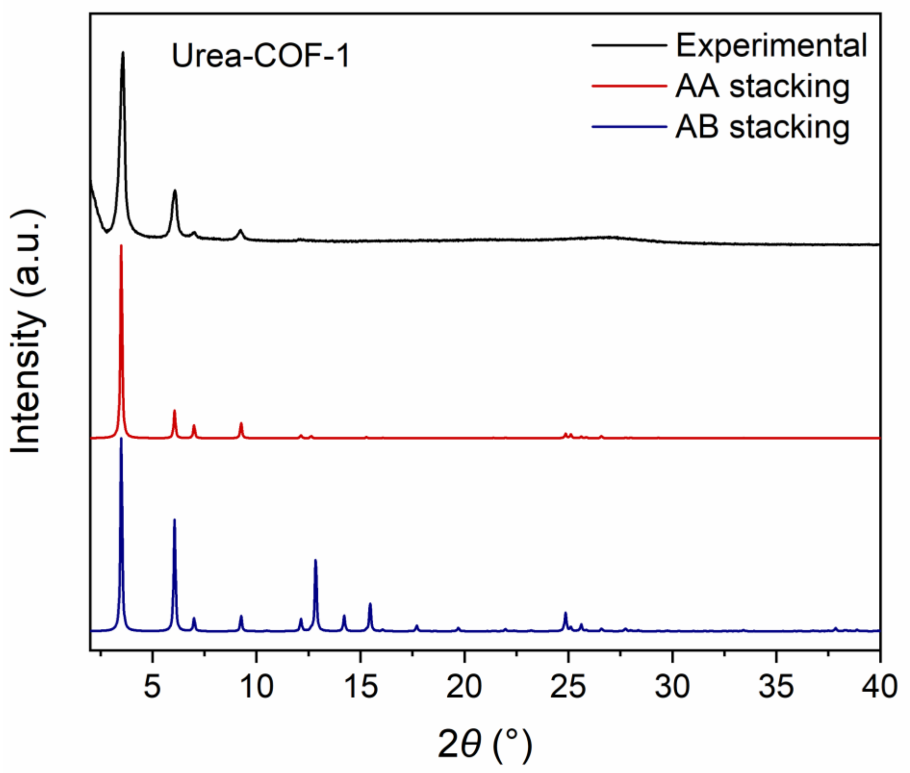

Supplementary Fig. 9 PXRD patterns for Urea-COF-1: experimental (dark), calculated with fully eclipsed AA-stacking (red) and staggered AB-stacking (blue) models. The eclipsed AA-stacking model yielded a PXRD pattern that was consistent with the experimental profile.

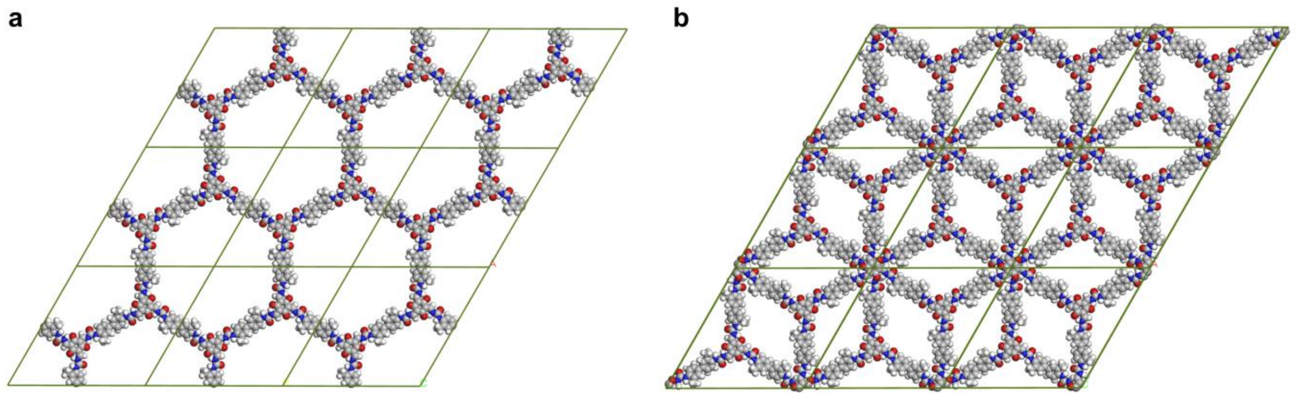

Supplementary Fig. 10 The top view of eclipsed AA-stacking (**a**) and staggered AB-stacking (**b**) models for Urea-COF-2.

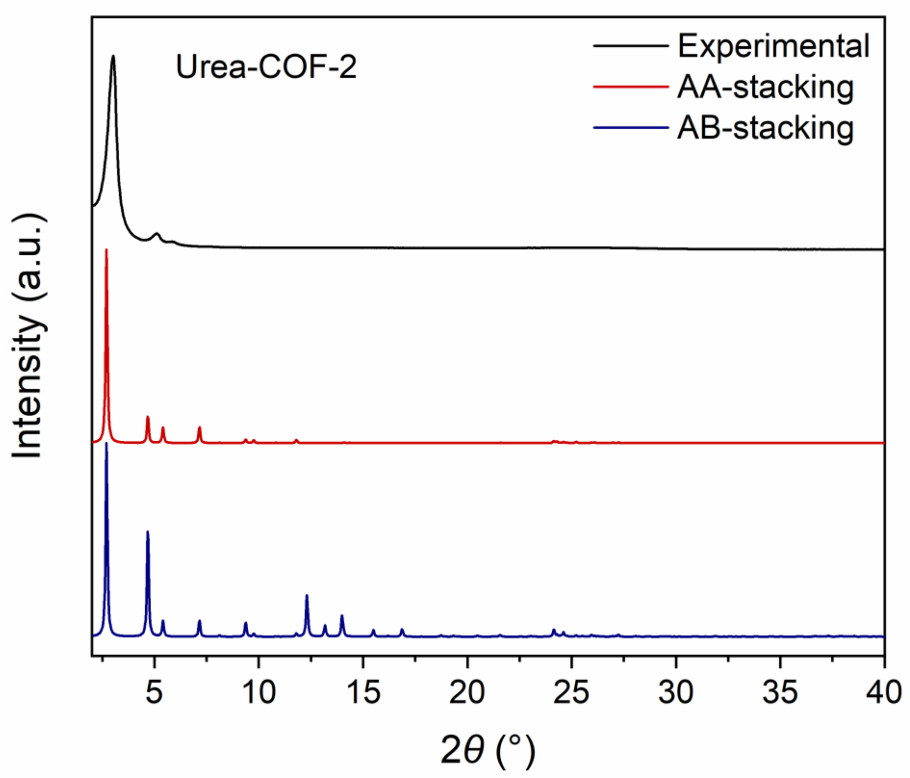

Supplementary Fig. 11 PXRD patterns for Urea-COF-2: experimental (dark), calculated with fully eclipsed AA-stacking (red) and staggered AB-stacking (blue) models. The eclipsed AA-stacking model yielded a PXRD pattern that was consistent with the experimental profile.

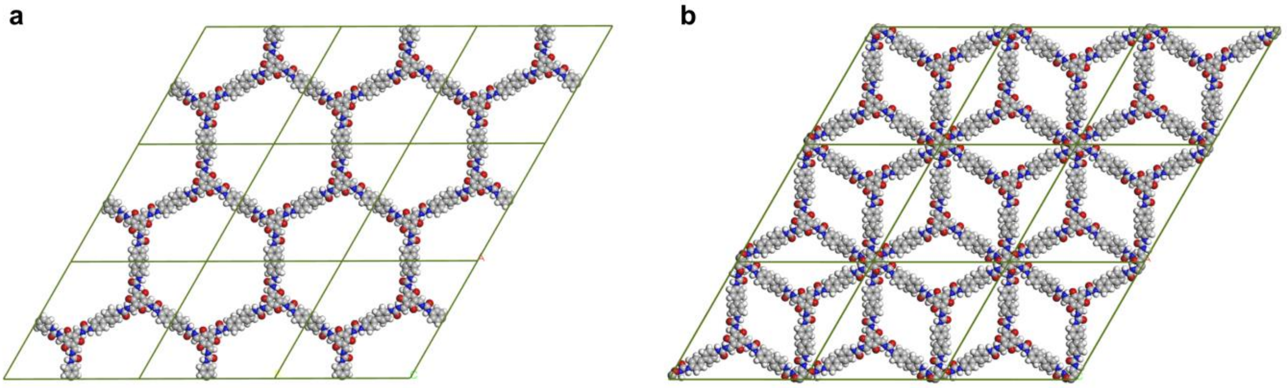

Supplementary Fig. 12 The top view of eclipsed AA-stacking (**a**) and staggered AB-stacking (**b**) models for Urea-COF-3.

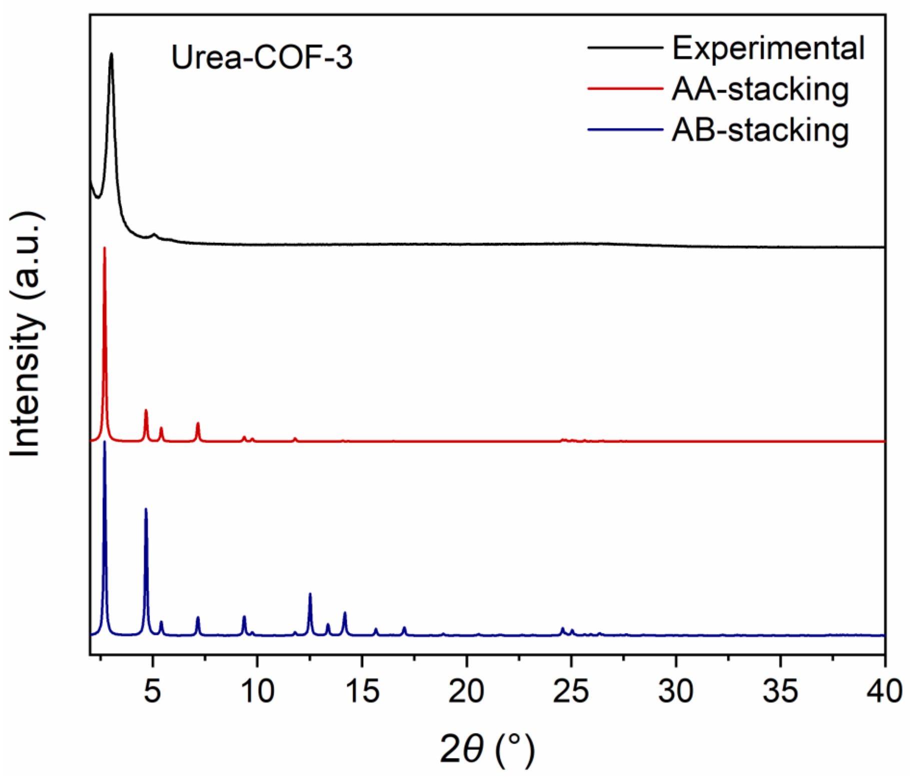

Supplementary Fig. 13 PXRD patterns for Urea-COF-3: experimental (dark), calculated with fully eclipsed AA-stacking (red) and staggered AB-stacking (blue) models. The eclipsed AA-stacking model yielded a PXRD pattern that was consistent with the experimental profile.

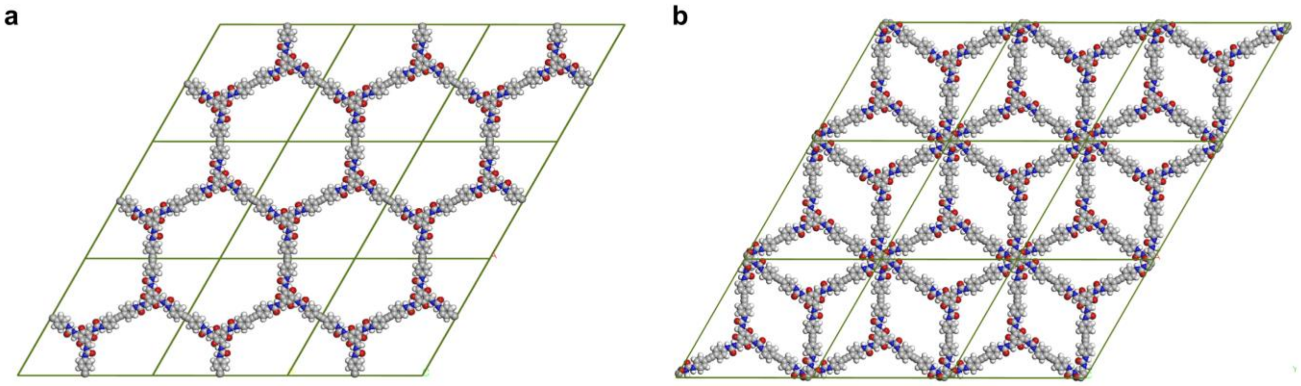

Supplementary Fig. 14 The top view of eclipsed AA-stacking (**a**) and staggered AB-stacking (**b**) models for Urea-COF-4.

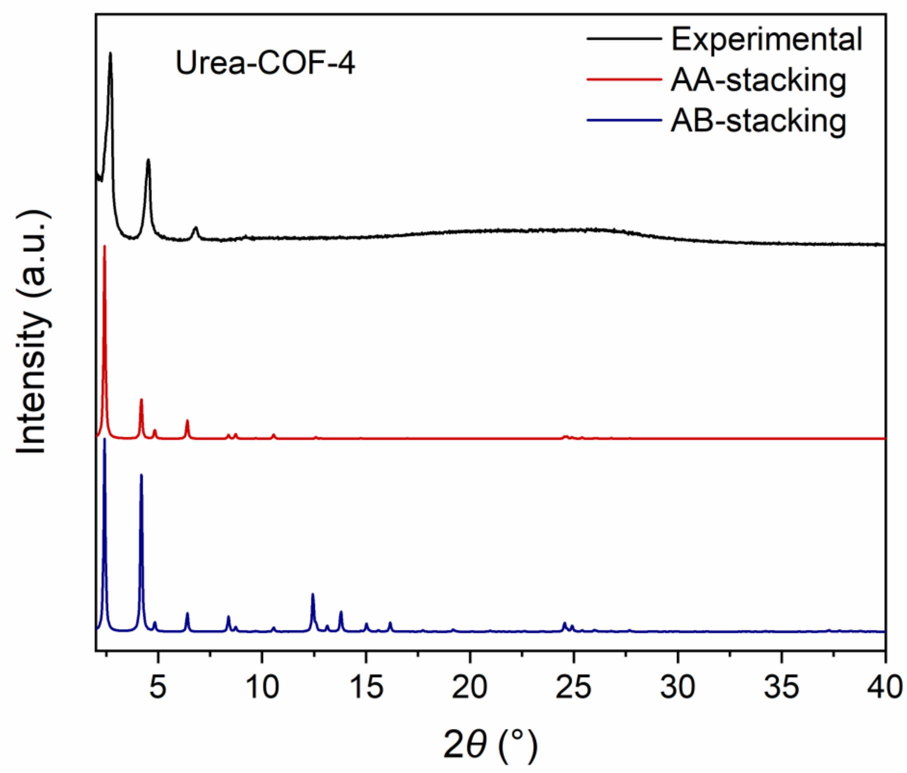

Supplementary Fig. 15 PXRD patterns for Urea-COF-4: experimental (dark), calculated with fully eclipsed AA-stacking (red) and staggered AB-stacking (blue) models. The eclipsed AA-stacking model yielded a PXRD pattern that was consistent with the experimental profile.

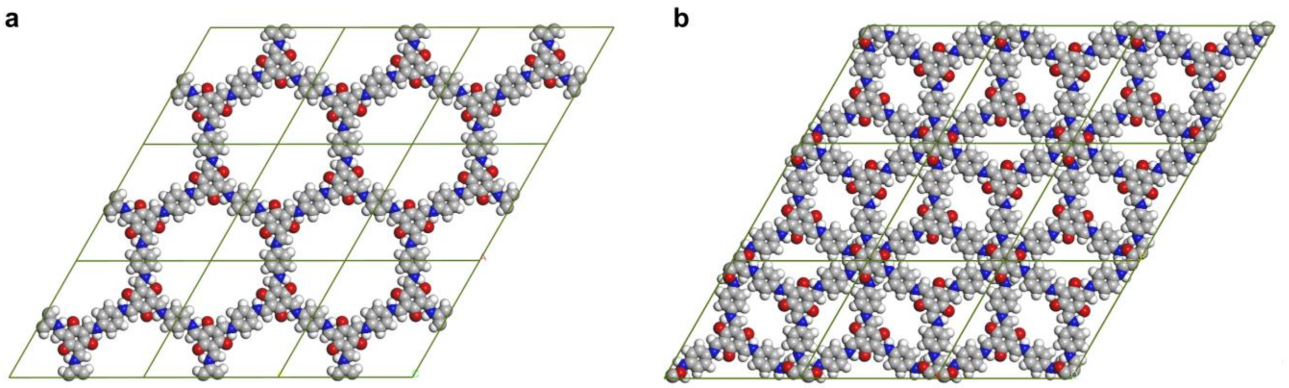

Supplementary Fig. 16 The top view of eclipsed AA-stacking (**a**) and staggered AB-stacking (**b**) models for RC-COF-1.

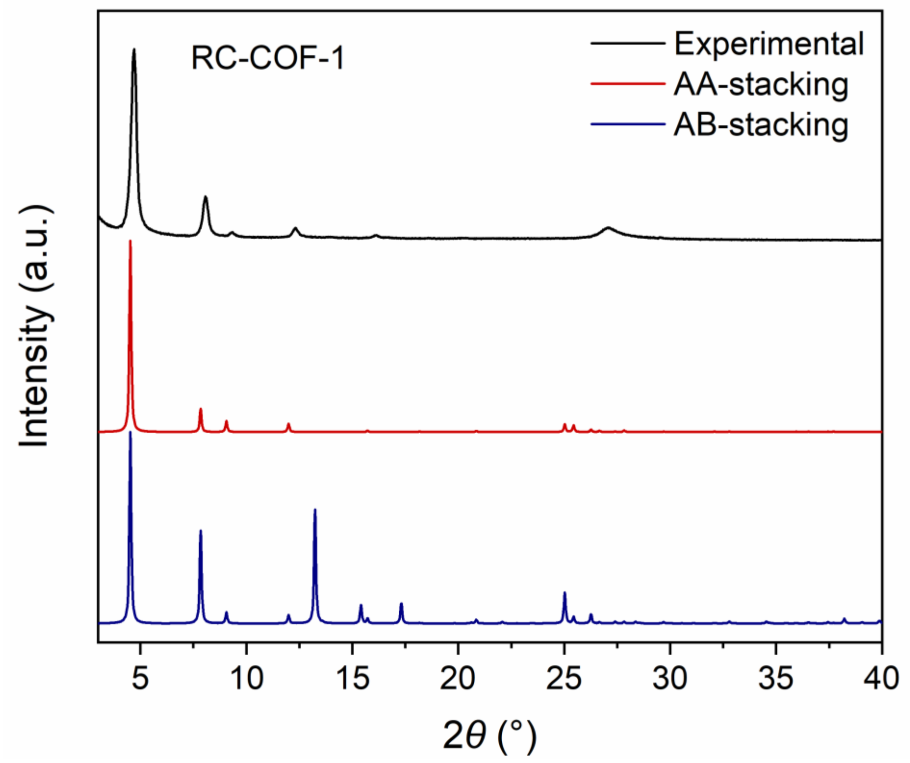

Supplementary Fig. 17 PXRD patterns for RC-COF-1: experimental (dark), calculated with fully eclipsed AA-stacking (red) and staggered AB-stacking (blue) models. The eclipsed AA-stacking model yielded a PXRD pattern that was consistent with the experimental profile.

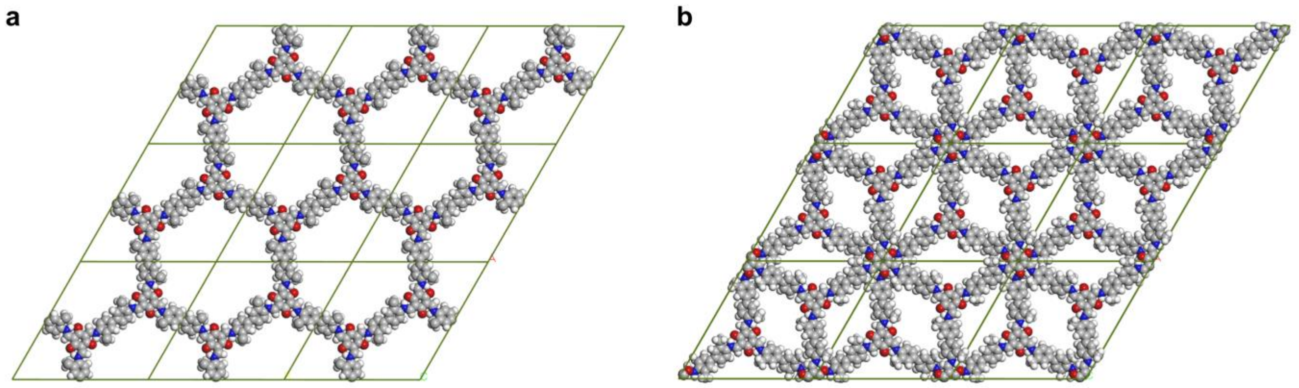

Supplementary Fig. 18 The top view of eclipsed AA-stacking (**a**) and staggered AB-stacking (**b**) models for RC-COF-2.

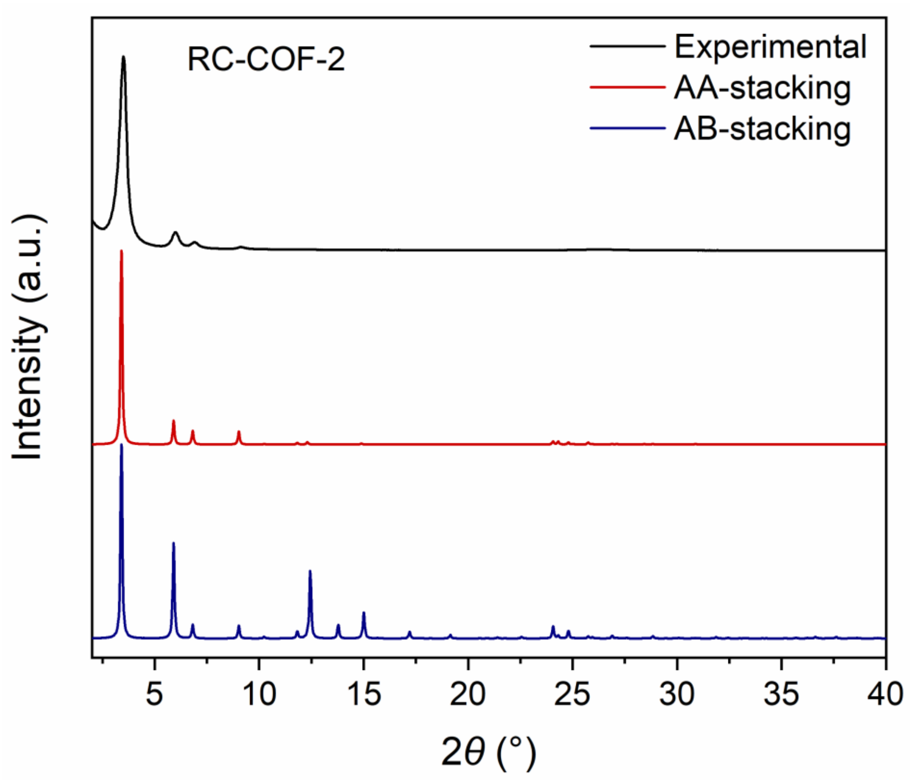

Supplementary Fig. 19 PXRD patterns for RC-COF-2: experimental (dark), calculated with fully eclipsed AA-stacking (red) and staggered AB-stacking (blue) models. The eclipsed AA-stacking model yielded a PXRD pattern that was consistent with the experimental profile.

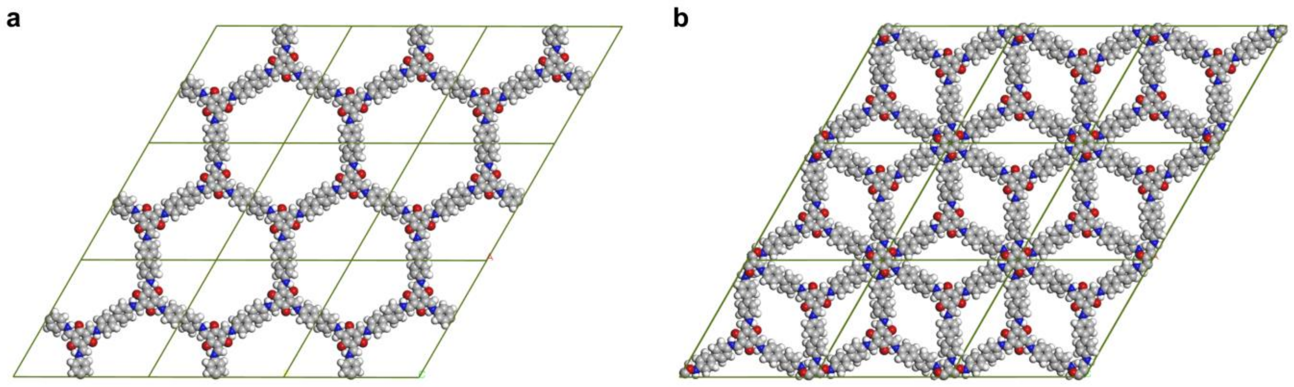

Supplementary Fig. 20 The top view of eclipsed AA-stacking (**a**) and staggered AB-stacking (**b**) models for RC-COF-3.

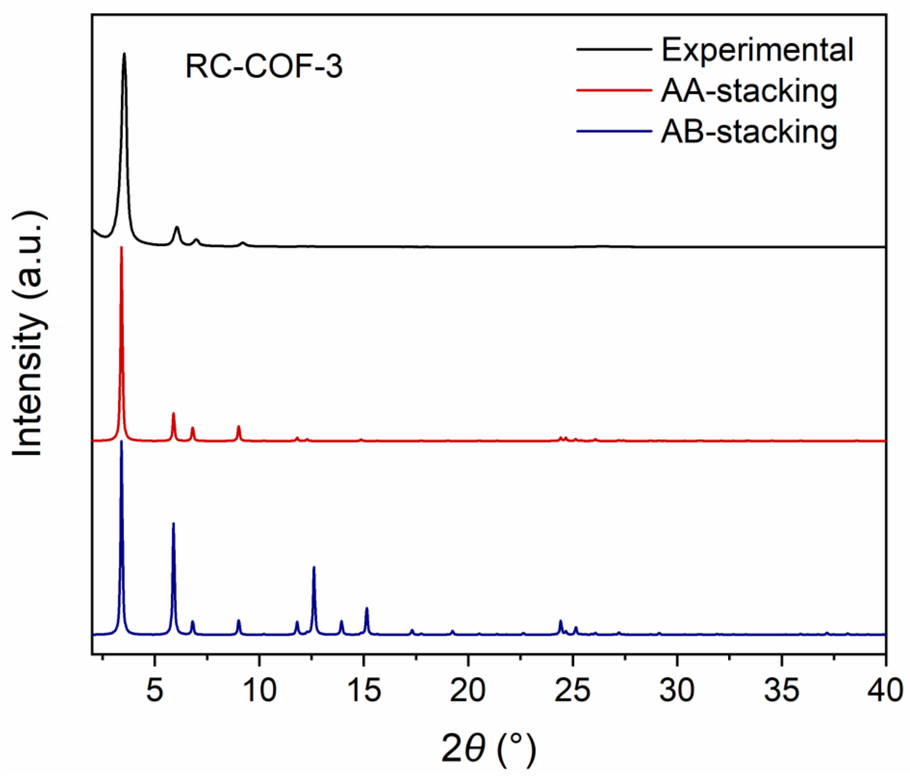

Supplementary Fig. 21 PXRD patterns for RC-COF-3: experimental (dark), calculated with fully eclipsed AA-stacking (red) and staggered AB-stacking (blue) models. The eclipsed AA-stacking model yielded a PXRD pattern that was consistent with the experimental profile.

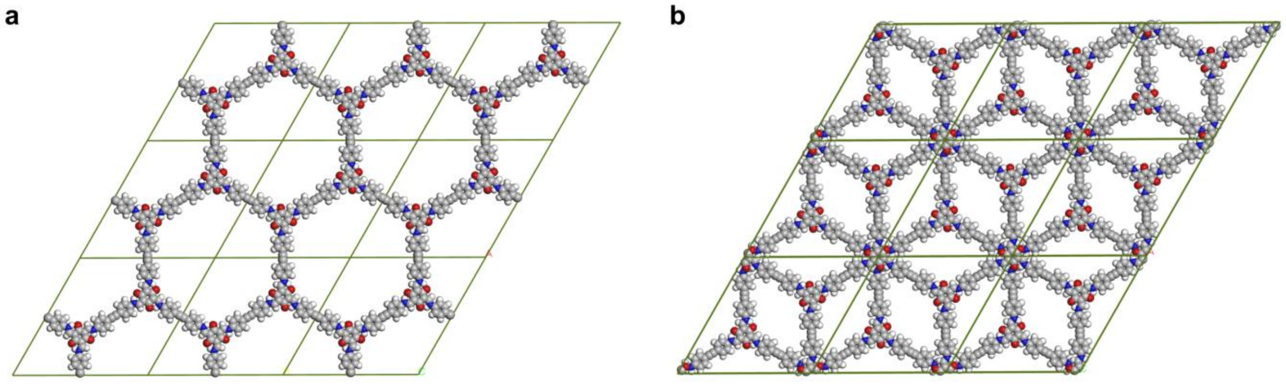

Supplementary Fig. 22 The top view of eclipsed AA-stacking (**a**) and staggered AB-stacking (**b**) models for RC-COF-4.

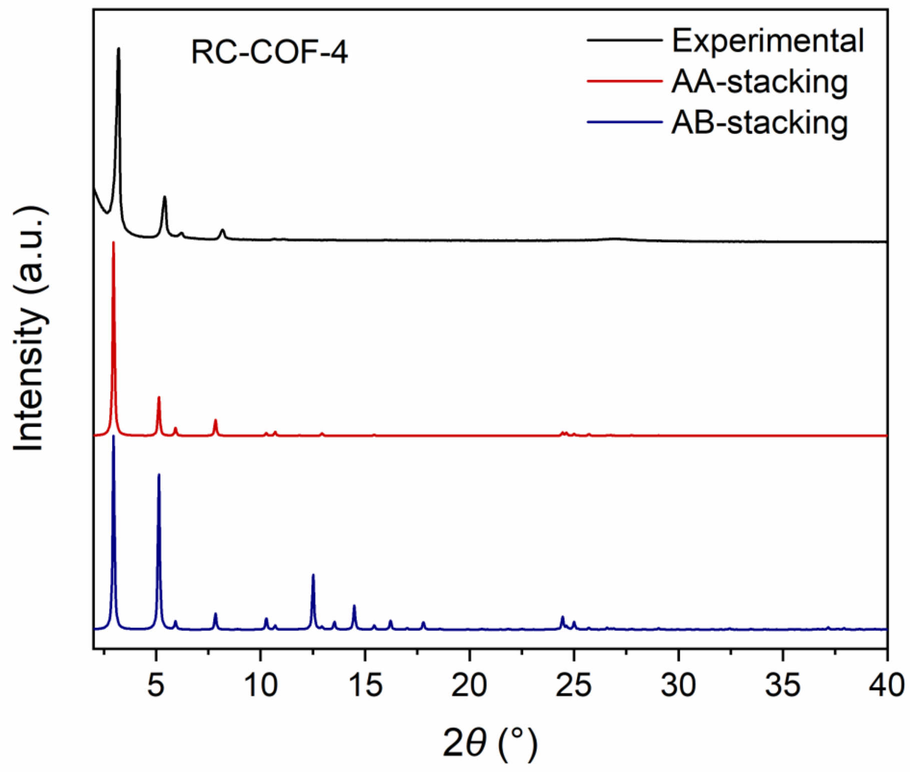

Supplementary Fig. 23 PXRD patterns for RC-COF-4: experimental (dark), calculated with fully eclipsed AA-stacking (red) and staggered AB-stacking (blue) models. The eclipsed AA-stacking model yielded a PXRD pattern that was consistent with the experimental profile.

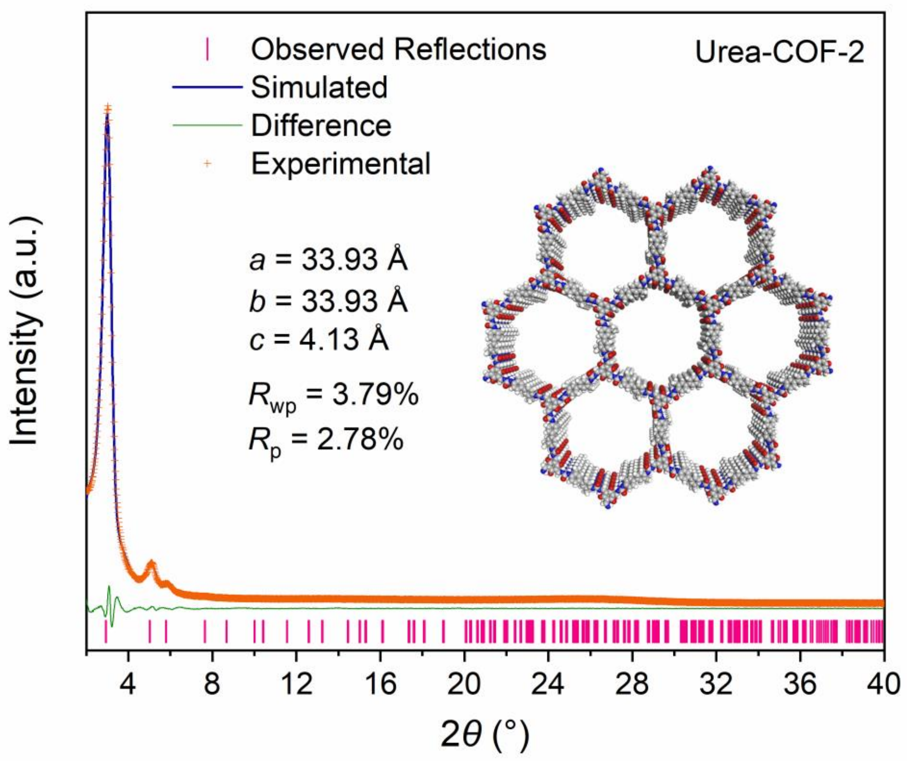

Supplementary Fig. 24 Simulated and experimental PXRD patterns for Urea-COF-2 (solvated). The structural model of Urea-COF-2 was built using Materials Studio and refined using experimental PXRD data.

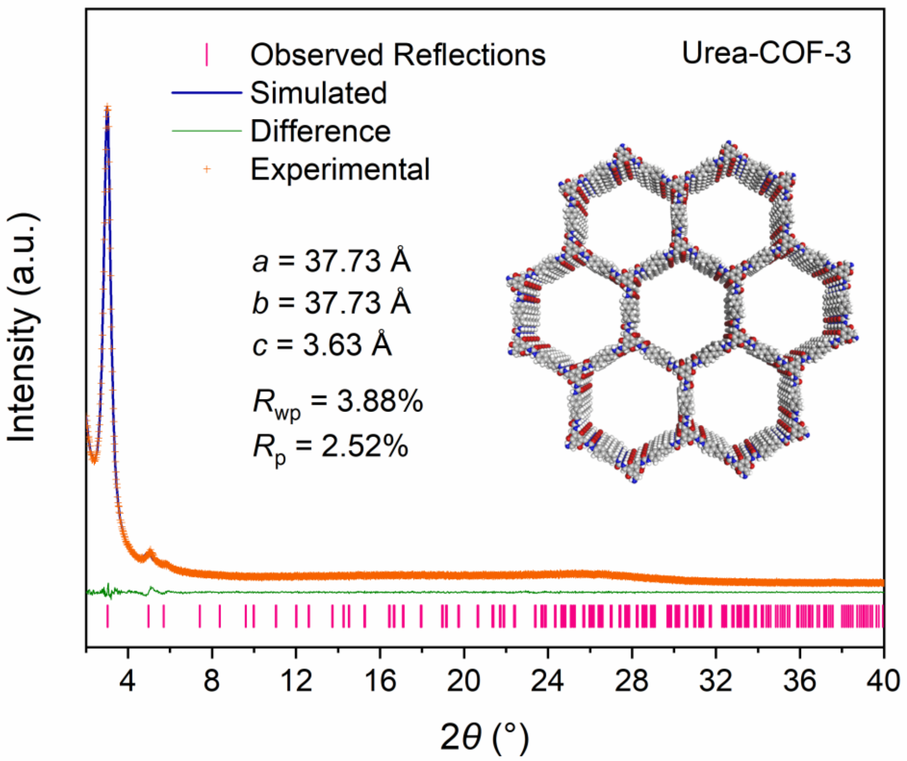

Supplementary Fig. 25 Simulated and experimental PXRD patterns for Urea-COF-3 (solvated).

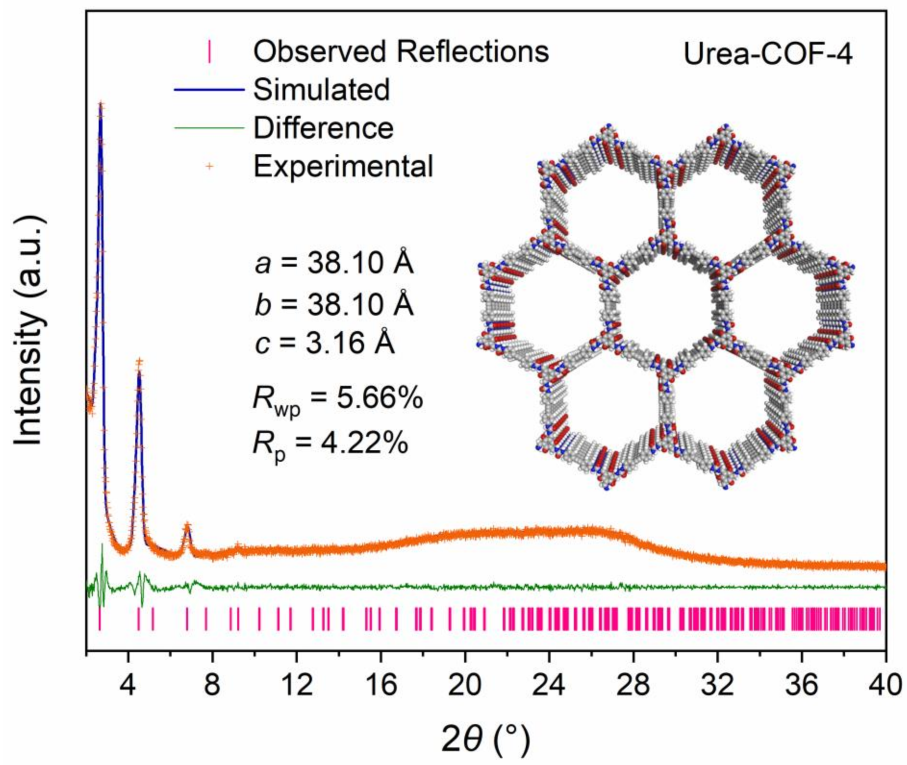

Supplementary Fig. 26 Simulated and experimental PXRD patterns for Urea-COF-4 (solvated).

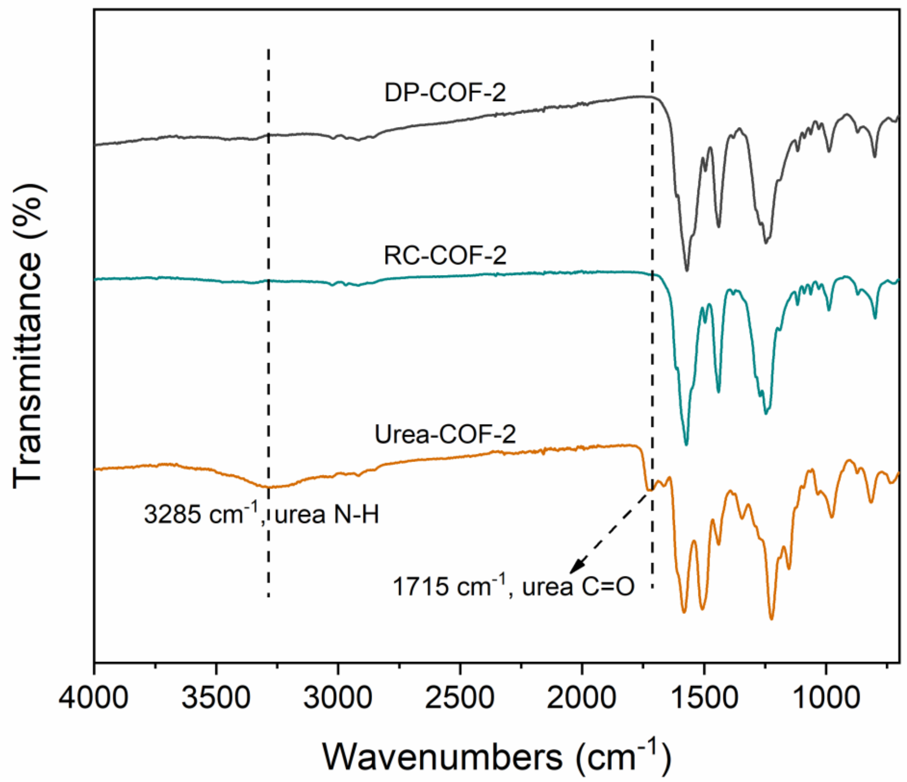

Supplementary Fig. 27 Comparison of FTIR spectra for DP-COF-2, RC-COF-2 and Urea-COF-2.

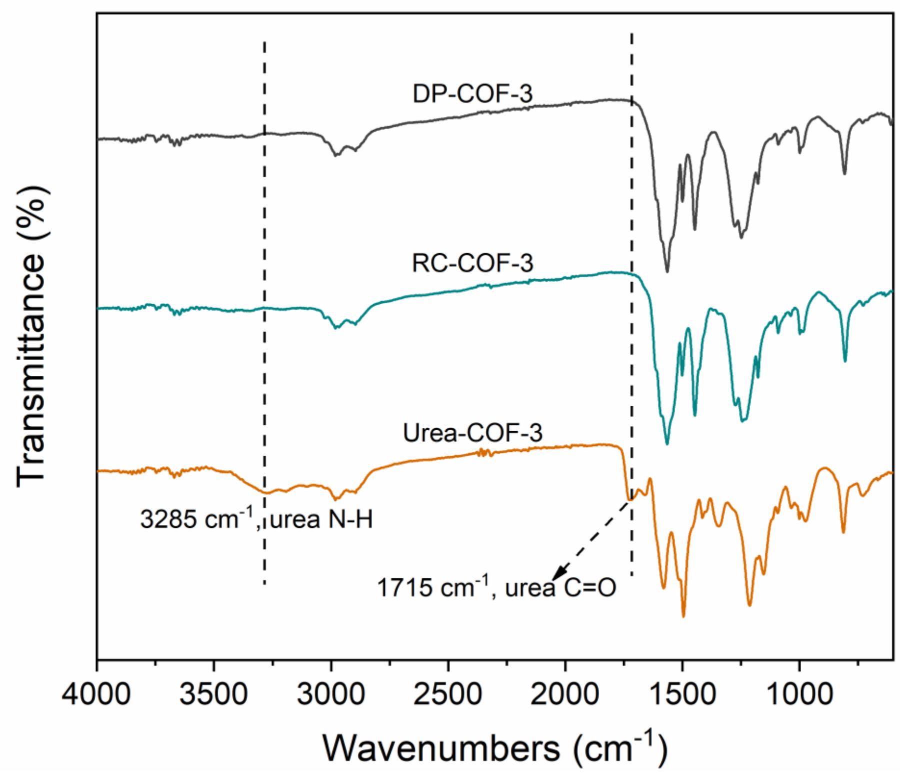

Supplementary Fig. 28 Comparison of FTIR spectra for DP-COF-3, RC-COF-3 and Urea-COF-3.

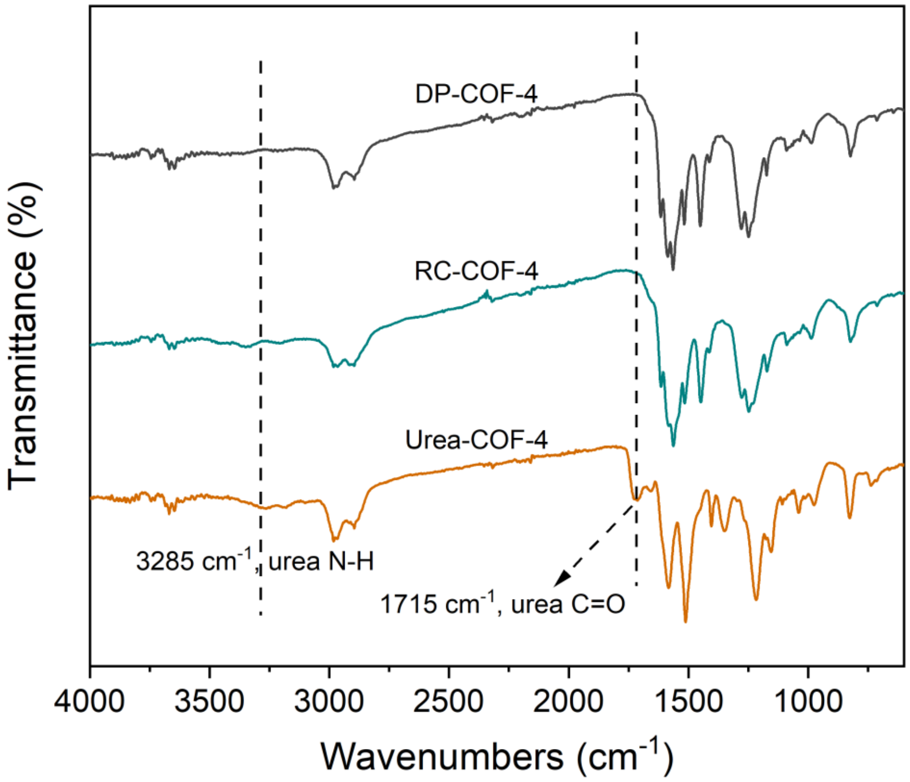

Supplementary Fig. 29 Comparison of FTIR spectra for DP-COF-4, RC-COF-4 and Urea-COF-4.

Section 5 Gas adsorption experiments

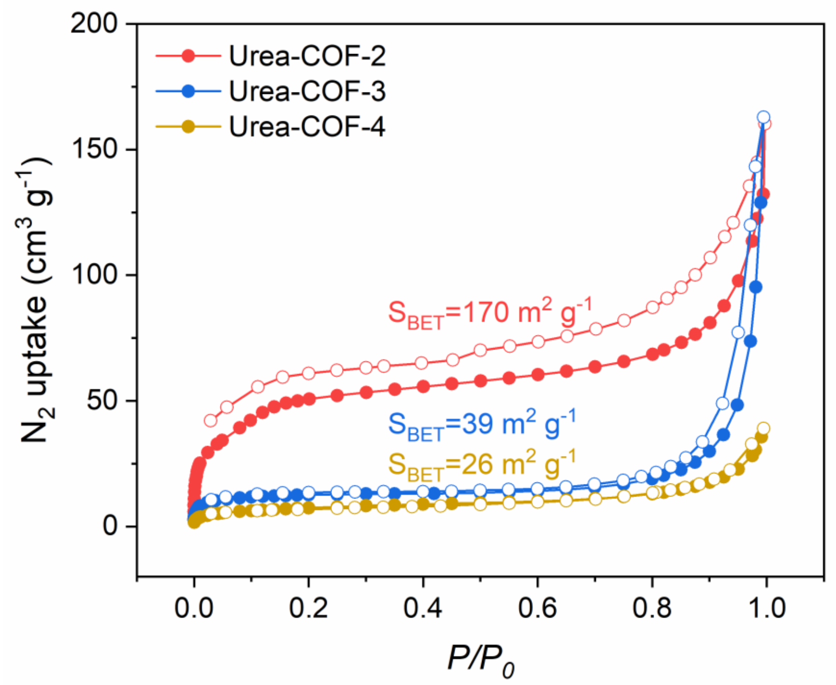

Supplementary Fig. 30 Nitrogen adsorption isotherm (filled symbols) and desorption isotherm (open symbols) for Urea-COF-2 (red), Urea-COF-3 (blue) and Urea-COF-4 (yellow) recorded at 77.3 K. Urea-COFs showed a low Brunauer-Emmett-Teller (BET) surface area because of pore deformation upon activation due to the flexible urea linkage.

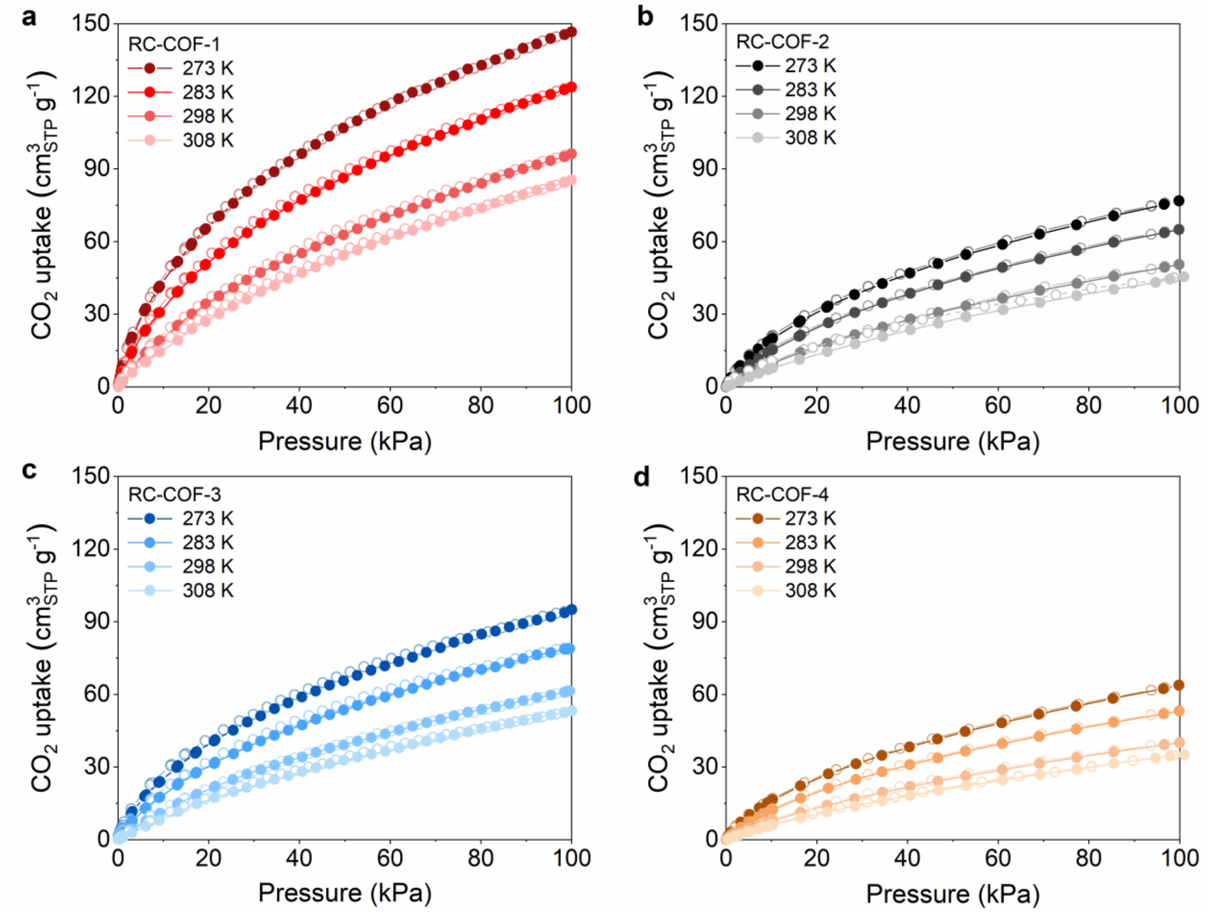

Supplementary Fig. 31 Adsorption (filled circles) and desorption (open circles) isotherms for CO_2 uptake in RC-COF-1 (**a**), RC-COF-2 (**b**), RC-COF-3 (**c**) and RC-COF-4 (**d**) recorded at 273, 283, 298 and 308 K. RC-COF-1 showed the highest CO_2 uptake in this series of materials.

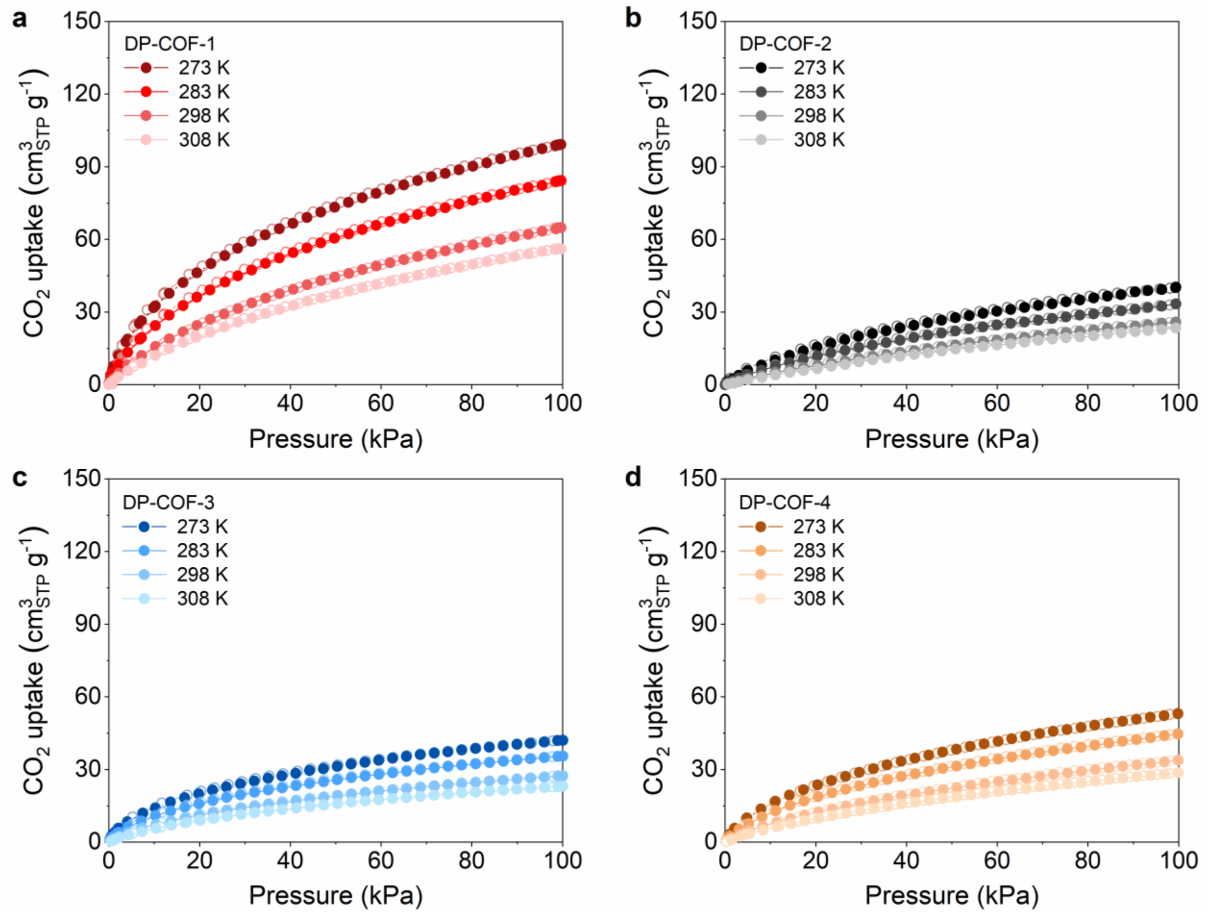

Supplementary Fig. 32 Adsorption (filled circles) and desorption (open circles) isotherms for CO_2 uptake in DP-COF-1 (**a**), DP-COF-2 (**b**), DP-COF-3 (**c**) and DP-COF-4 (**d**) recorded at 273, 283, 298 and 308 K. DP-COFs showed significantly lower CO_2 uptake compared the equivalent RC-COFs measured under the same conditions.

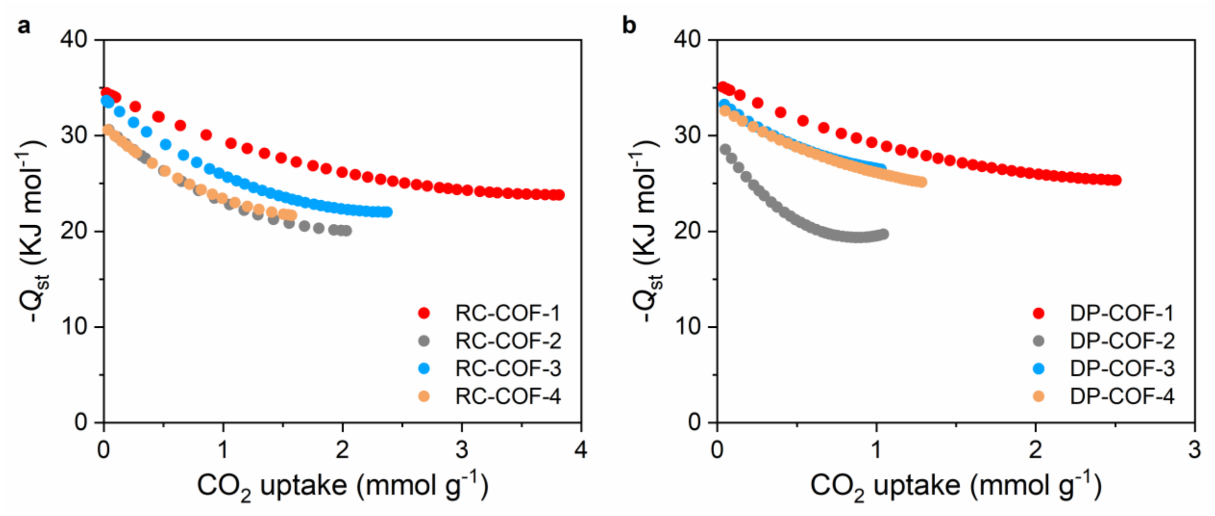

Supplementary Fig. 33 Isosteric heat of adsorption ($-\ Q_{st}$) as a function of the amount of CO_2 adsorbed for RC-COFs (**a**) and DP-COFs (**b**). The isosteric heat was calculated using CO_2 adsorption data recorded at 273, 283, 298 and 308 K. The calculated heat of adsorption is ~ 35 kJ mol^{-1} for RC-COF-1 at the onset of adsorption.

Section 6 Thermogravimetric analyses

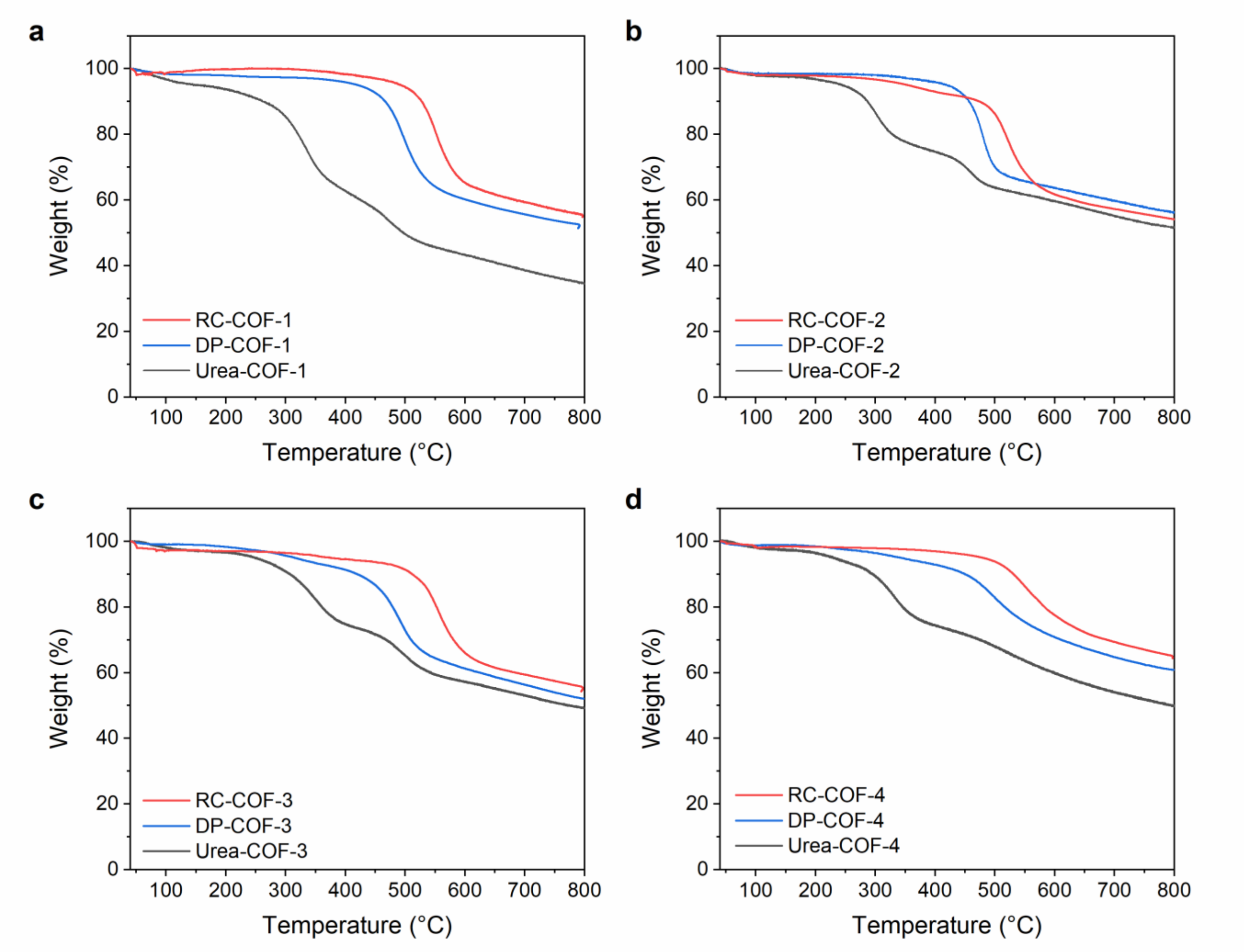

Supplementary Fig. 34 TGA traces for RC-COF-1, DP-COF-1 and Urea-COF-1 (**a**), RC-COF-2, DP-COF-2 and Urea-COF-2 (**b**), RC-COF-3, DP-COF-3 and Urea-COF-3 (**c**), and RC-COF-4, DP-COF-4 and Urea-COF-4 (**d**) under nitrogen atmosphere. The reconstructed COFs with higher crystallinity show better thermal stability than their semi-crystalline analogs synthesized by direct polymerization. Note that the small mass loss observed < 200 °C is ascribed to desorption of guests from the COF pores, mostly physiosorbed water vapour.

Section 7 Scanning electron microscopy and transmission electron microscopy images

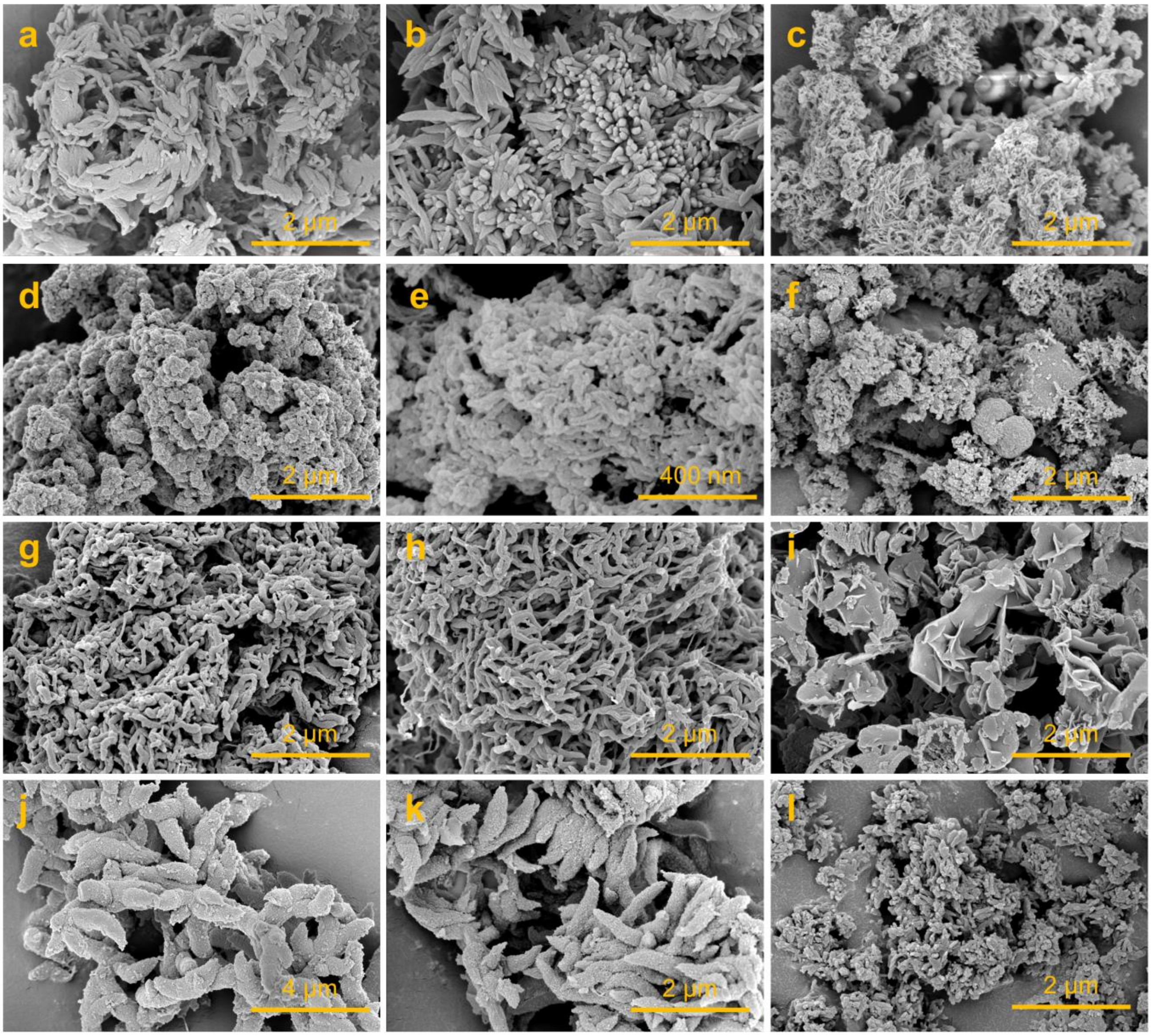

Supplementary Fig. 35 SEM images for Urea-COF-1 (**a**), RC-COF-1 (**b**), DP-COF-1 (**c**), Urea-COF-2 (**d**), RC-COF-2 (**e**), DP-COF-2 (**f**), Urea-COF-3 (**g**), RC-COF-3 (**h**), DP-COF-3 (**i**), Urea-COF-4 (**j**), RC-COF-4 (**k**) and DP-COF-4 (**l**). Note that these small crystallites have a significant surface-to-volume ratio, and surface end-groups might contribute to the non-ideal elemental analysis values that we observe (Supplementary Table 1).

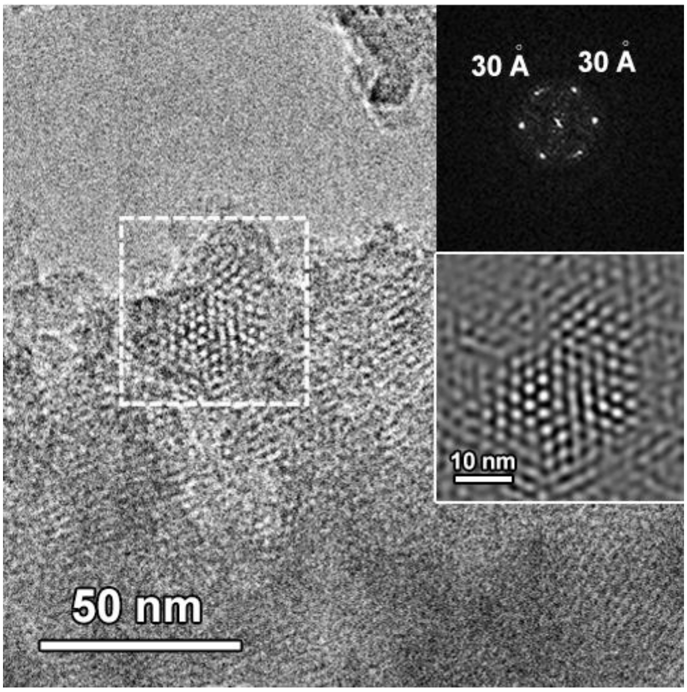

Supplementary Fig. 36 HRTEM images for RC-COF-4; insets show the fast Fourier transform (FFT) pattern taken from the regions highlighted by the dashed-line squares and the corresponding filtered inverse fast Fourier transform image.

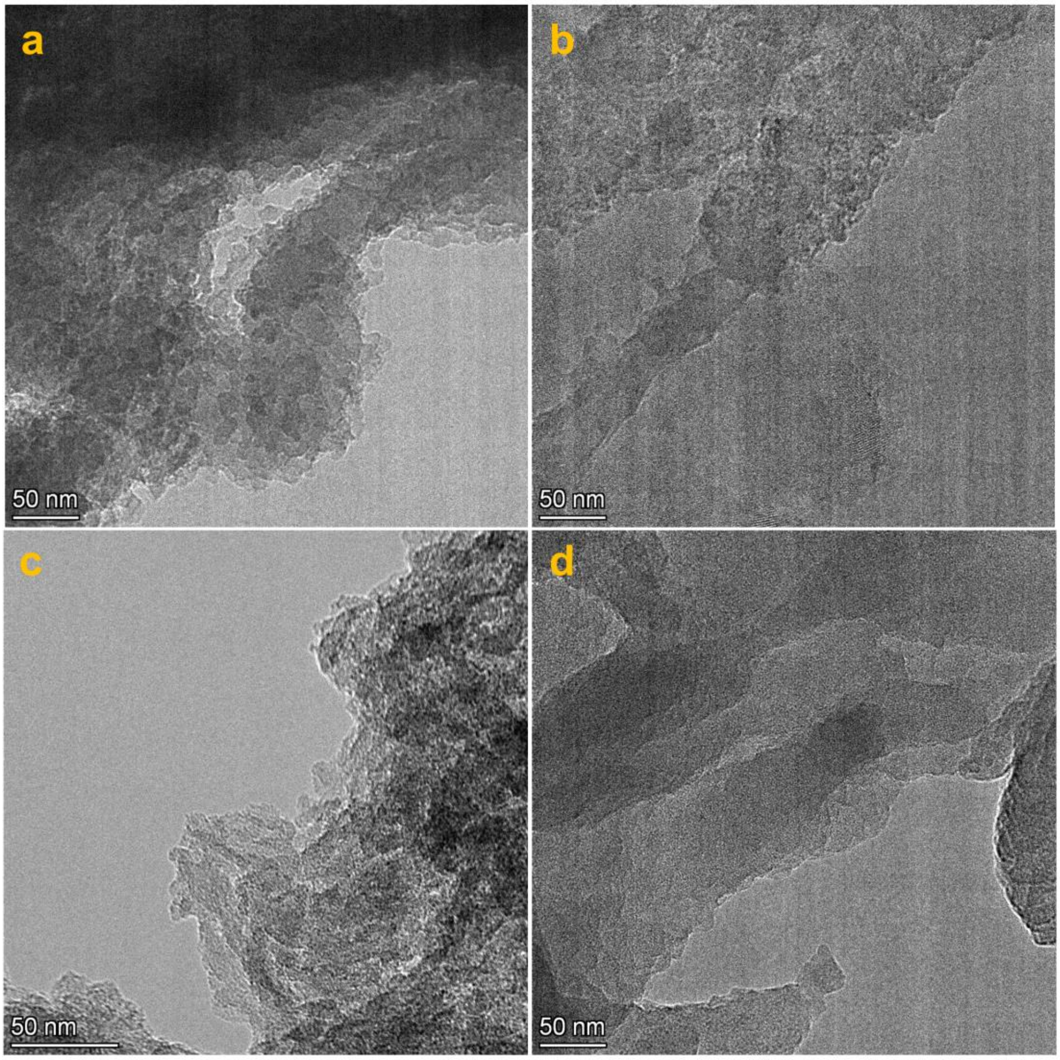

Supplementary Fig. 37 HRTEM images for DP-COF-1 (**a**), DP-COF-2 (**b**), DP-COF-3 (**c**) and DP-COF-4 (**d**). In contrast to the analogous RC-COFs, no clear lattice fringes could be discerned.

Section 8 Control experiments: hydrothermal treatment

To assess the possibility that enhanced crystallinity was the result of hydrothermal cycling of the imine COFs, rather than preorganization, we performed a series of control experiments involving post-synthetic hydrothermal treatment of the four DP-COFs under precisely the same conditions as used for reconstruction (H_2O, 160 °C, 72 hours). PXRD was used to assess the crystallinity of the frameworks before and after hydrothermal treatment. As shown in Supplementary Fig. 38, below, PXRD suggested no change in crystallinity for the DP-COFs, apart from DP-COF-4, which showed a notable *decrease* in crystallinity. Likewise, no improvement in surface area was observed after hydrothermal treatment for DP-COF-2, DP-COF-3, or DP-COF-4. The surface area for DP-COF-1 was somewhat improved by hydrothermal treatment, but it was still around half the value measured for RC-COF-1. These data show that the improvement in crystallinity in the RC-COFs is not a result of simple hydrothermal cycling or annealing of the resultant imine COFs. The greatly improved levels of crystallinity and porosity are only obtained via reconstruction of the highly crystalline urea COFs, and the associated preorganization and nanoconfined polymerization.

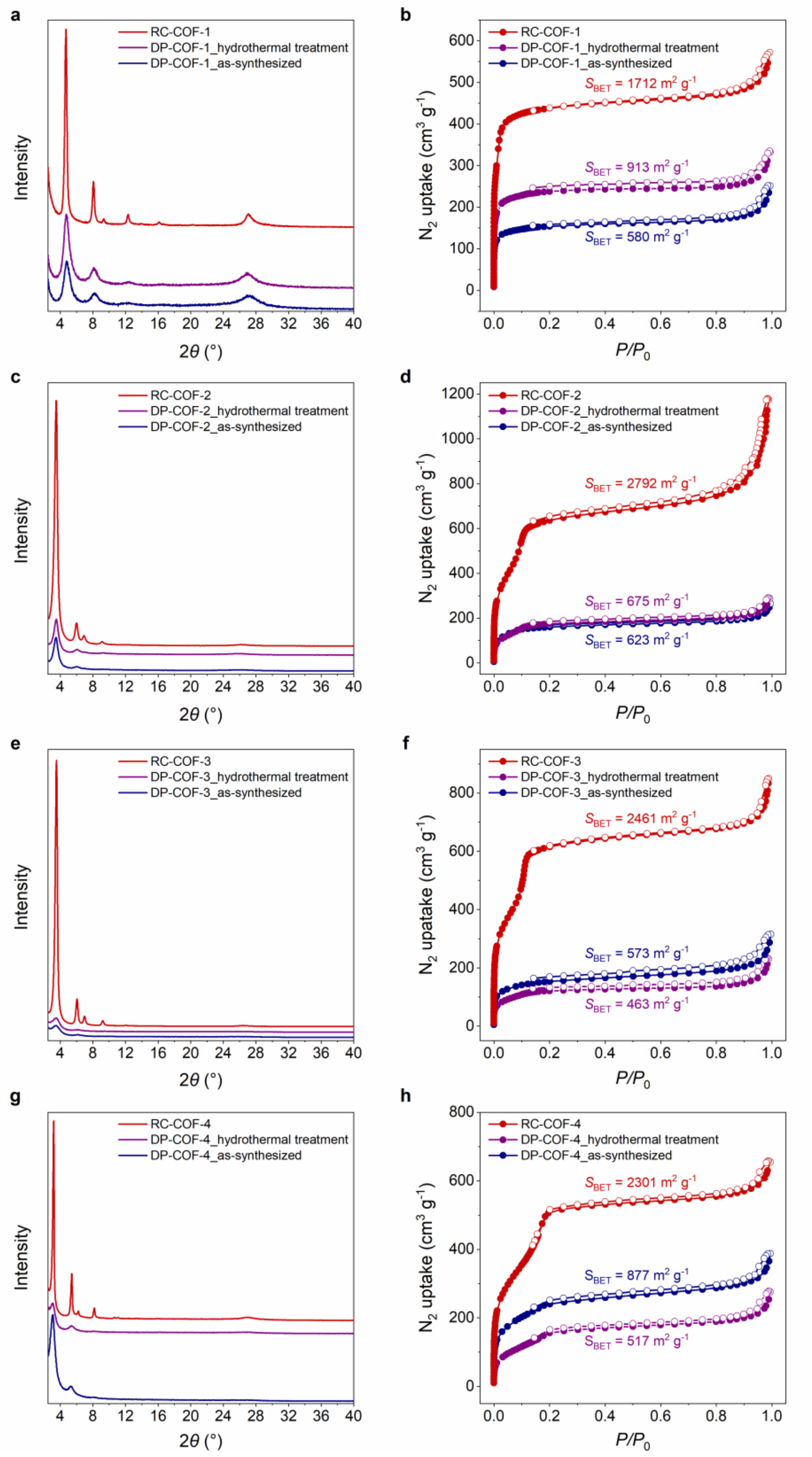

Supplementary Fig. 38 Comparison of PXRD patterns (**a**, **c**, **e**, **g**) and nitrogen adsorption isotherms / BET surface areas (**b**, **d**, **f**, **h**) for RC-COFs along with DP-COFs, both as-synthesized and after hydrothermal treatment (H_2O, 160 °C, 72 hours). The high levels of crystallinity and porosity observed in the RC-COFs are not attained by hydrothermal treatment of DP-COFs; rather, it is a result of preorganization in the urea COF precursors, coupled with nanoconfined reconstruction.

Section 9 Characterizations of RC-COF-1 synthesized without vacuum degassing step

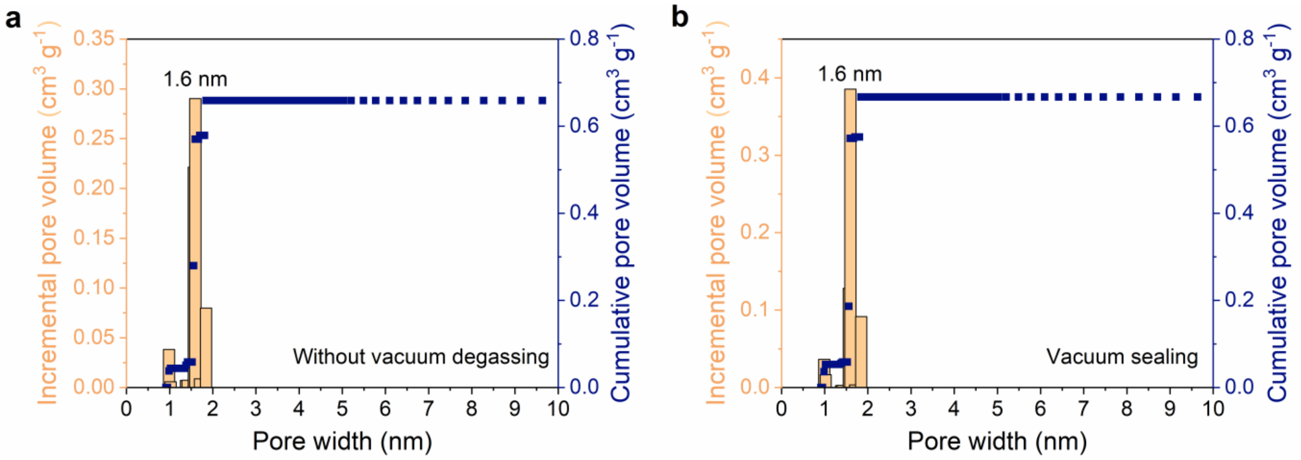

Supplementary Fig. 39 Pore size distribution profiles for RC-COF-1 synthesized without vacuum degassing step (**a**) and by vacuum sealing (**b**). The pore volumes were 0.83 and 0.81 cm^3 g^{-1}, respectively (that is, the same, within error).

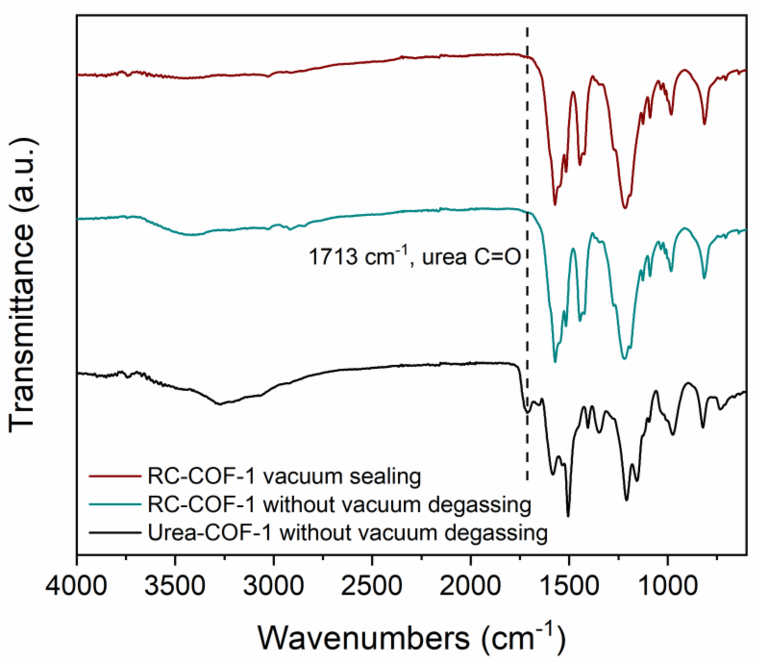

Supplementary Fig. 40 Comparison of FTIR spectra for Urea-COF-1 and RC-COF-1 synthesized without vacuum degassing step and with vacuum sealing.

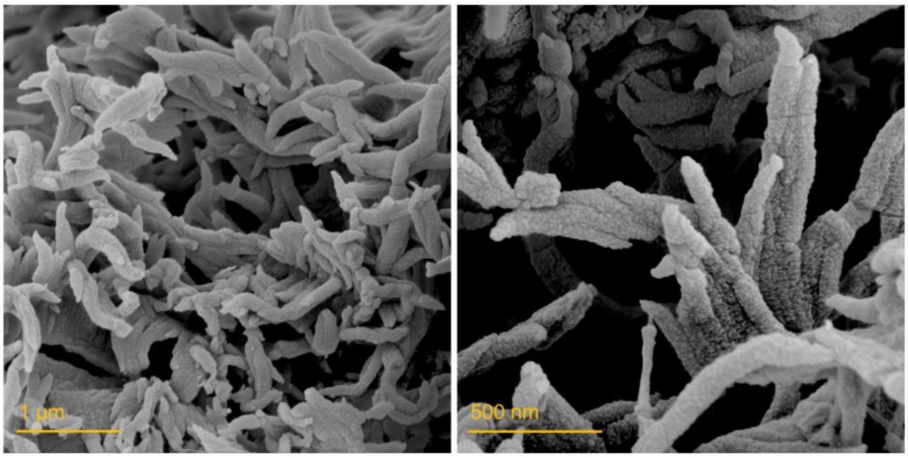

Supplementary Fig. 41 SEM images for RC-COF-1 synthesized without vacuum degassing step.

Section 10 Computational results

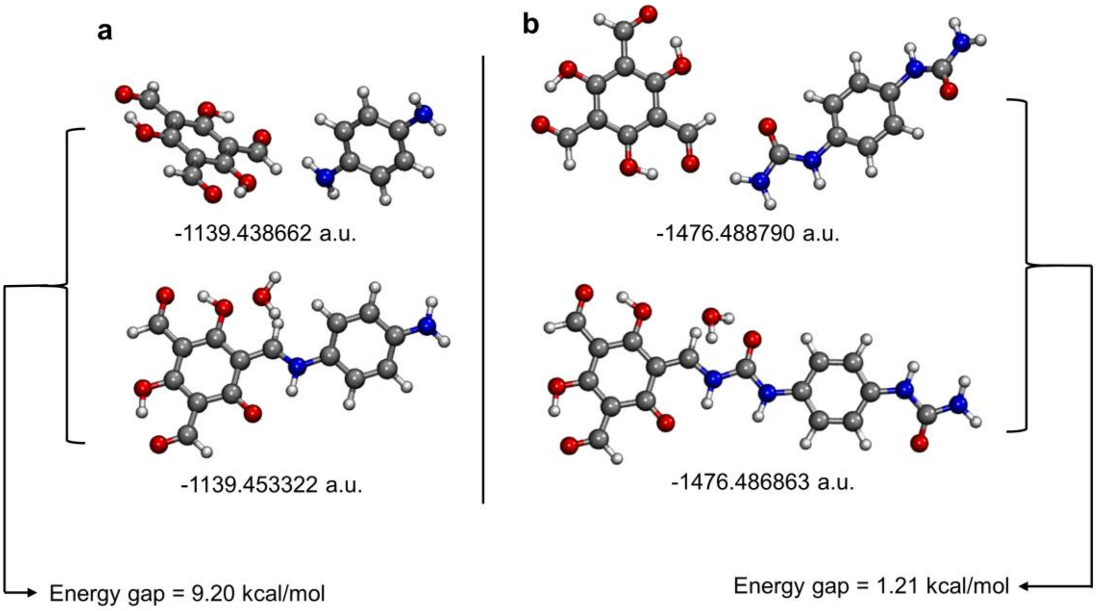

Supplementary Fig. 42 Gas phase conformers of reactants (**a**, top) and product (**a**, bottom) of *β*-ketoenamine model compound, and reactants (**b**, top) and product (**b**, bottom) of urea-linked model compound after DFT optimization. Energies below conformers are sum of electronic and thermal free energies. The corresponding energy gaps between reactants and products are shown in the figure.

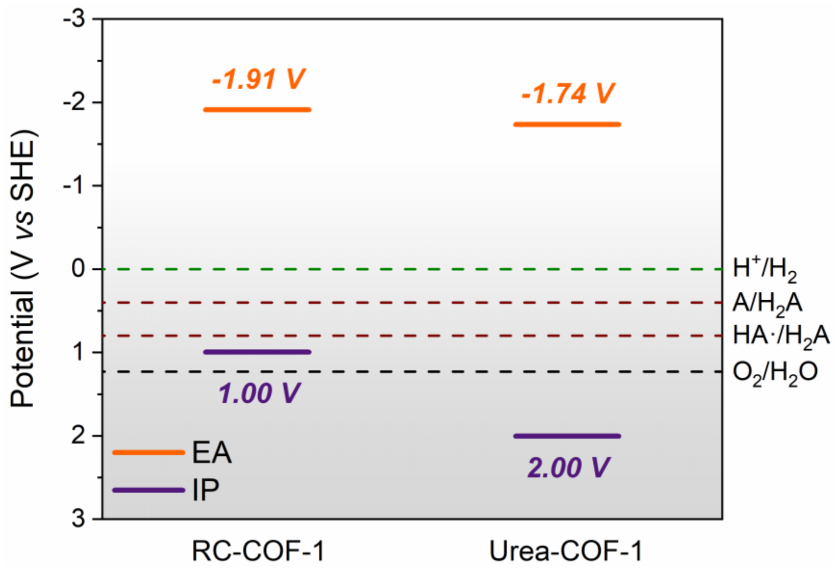

Supplementary Fig. 43 (TD-)B3LYP predicted ionization potential (IP) and electron affinity (EA) adiabatic potentials for RC-COF-1 and Urea-COF-1 in water.

Section 11 Optical and electronic properties

Highly ordered COF lattices should allow for efficient photo-generated charge migration with fewer trapping sites. This is desirable in optoelectronic applications and in photocatalysis, particularly when coupled with high physical surface areas. First, the UV-visible reflectance spectra of the COFs were measured in the solid state to determine their optical gaps (Extended Data Fig. 6e). RC-COF-1 and DP-COF-1 showed a broadened visible-light absorption response compared to Urea-COF-1. Kubelka-Munk analysis gave an optical band gap of ~ 2.12 eV for Urea-COF-1, and narrower optical gaps of ~ 1.87 eV and ~ 1.90 eV for RC-COF-1 and DP-COF-1, respectively (Supplementary Fig. 44). This is attributed to the enhanced conjugation backbone and intramolecular charge transfer from the phenylenediamine donor to the triketone acceptor. Room temperature steady-state photoluminescence (PL) spectroscopy was performed on these COFs with an excitation wavelength of 400 nm. It showed a decreased intensity at ~ 620 nm for RC-COF-1 compared to DP-COF-1 (Supplementary Fig. 45), suggesting a larger barrier for charge recombination in the highly crystalline reconstructed framework[6]. Time-correlated single-photon counting (TCSPC) was used to estimate the excited-state lifetimes (Supplementary Fig. 46, Table 2), and RC-COF-1 exhibited a longer average weighted lifetime (τ_{avg} = 3.93 ns) with respect to DP-COF-1 (τ_{avg} = 1.55 ns) in aqueous suspensions. Electron paramagnetic resonance (EPR) studies were also used to explore the electronic band structures (Extended Data Fig. 6a). A single Lorentzian line centered at a *g* value of 2.006 was observed for RC-COF-1 which intensified dramatically upon light excitation, suggesting an effective light-induced charge carrier generation[7], whereas DP-COF-1 displayed much lower intensity under same test conditions. The charge transfer in these COFs was also investigated by photo-electrochemistry. Electrochemical impedance spectroscopies (EIS) were measured with COF films under both dark and light conditions. The Nyquist plots for RC-COF-1 showed a semicircle with a smaller diameter than that was observed for DP-COF-1, suggesting an improved interfacial charge transport (Supplementary Fig. 47). Photocurrent measurements showed that RC-COF-1 produced a significantly enhanced photocurrent compared to its semi-crystalline counterpart, DP-COF-1, indicating more efficient separation of photogenerated charge carriers (Extended Data Fig. 6b). Hence, while RC-COF-1 is significantly more porous than DP-COF-1, and has a much lower bulk density, it nonetheless exhibits markedly better charge carrier transport. We attribute this to its greatly improved crystallinity.

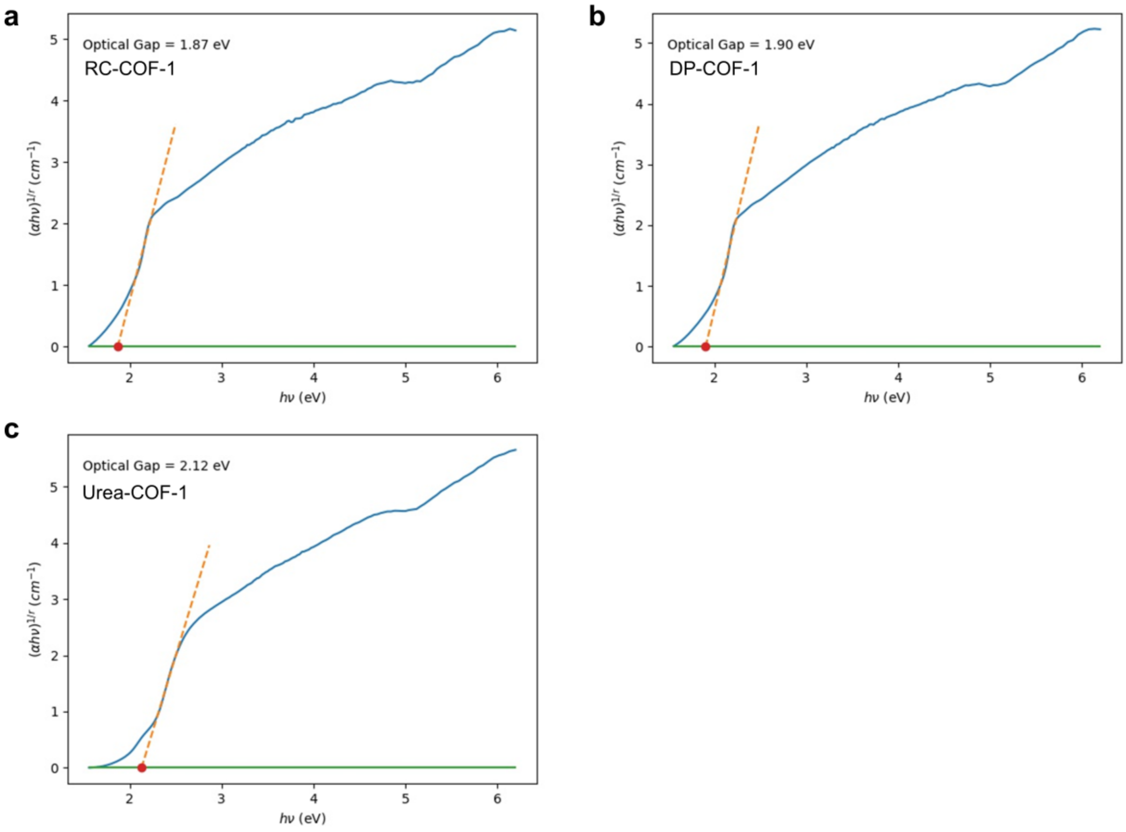

Supplementary Fig. 44 Plots of the Kubelka-Munk function to extract the direct optical band gap of RC-COF-1 (**a**), DP-COF-1 (**b**) and Urea-COF-1 (**c**).

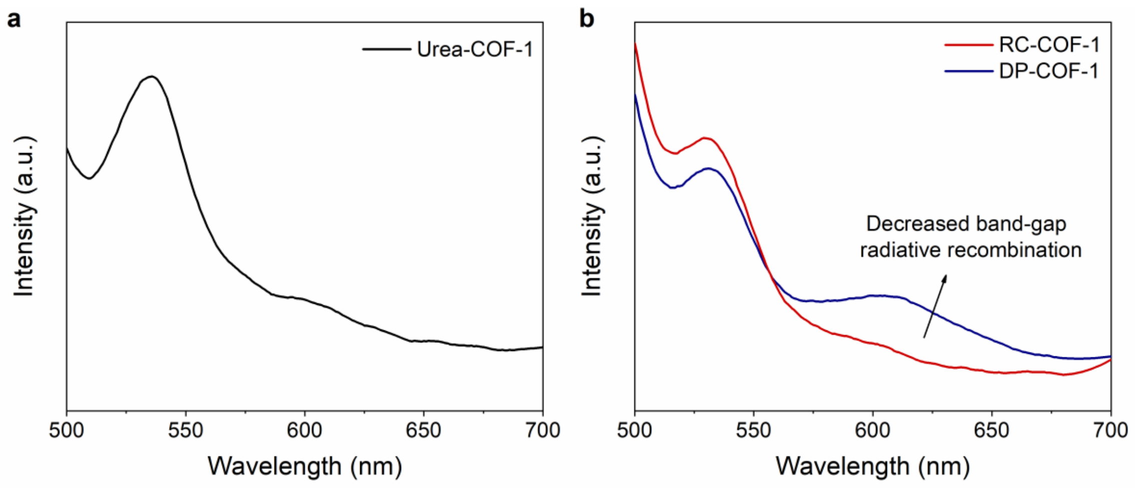

Supplementary Fig. 45 Room temperature (298 K) steady-state photoluminescence (PL) spectrum for Urea-COF-1 (**a**), and RC-COF-1 and DP-COF-1 (**b**) under 400 nm excitation. **b** indicates a decreased band-gap radiative recombination in the highly crystalline RC-COF-1.

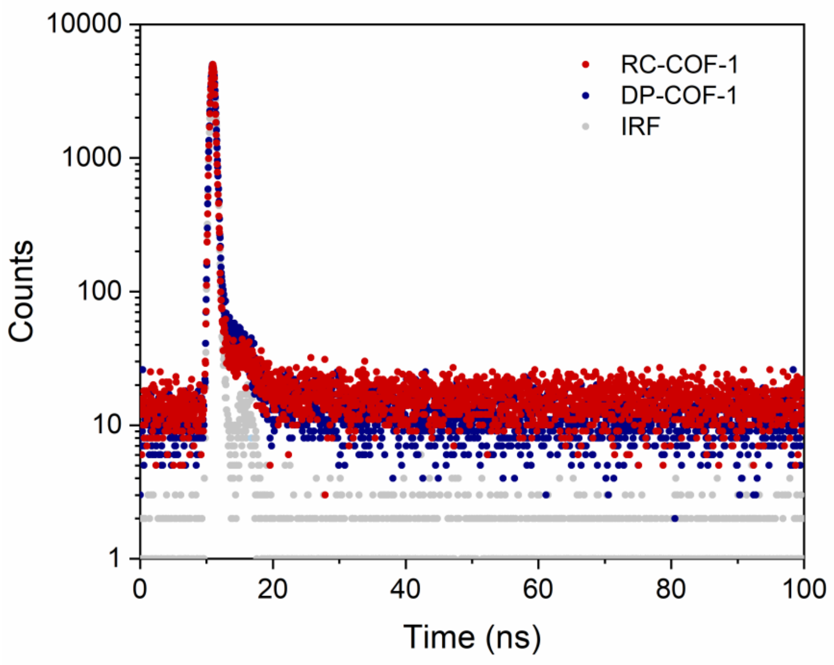

Supplementary Fig. 46 Time-correlated single photon counting (TCSPC) experiments for RC-COF-1 and DP-COF-1 in water. Samples were excited with a λ_{exc} = 370.5 nm laser and emission was measured at λ_{em} = 650 nm.

Supplementary Table 2 Fitting parameters for time correlated fluorescence lifetime experiments for RC-COF-1 and DP-COF-1 (λ_{exc} = 370.5 nm and λ_{em} = 650 nm).

**Sample**	**τ_1 (ns)**	**τ_2 (ns)**	**τ_3 (ns)**	**A_1**	**A_2**	**A_3**	**τ (ns)**
RC-COF-1	0.56	0.49	80.55	5.44	4.72	10.62	3.93
DP-COF-1	0.56	1.62	38.06	4.43	9.99	11.50	1.55

τ is the average lifetime calculated from the following equation:

$$\tau = \sum_{i=1}^{3}(A_i\tau_i^2) \Big/ \sum_{i=1}^{3}(A_i\tau_i)$$

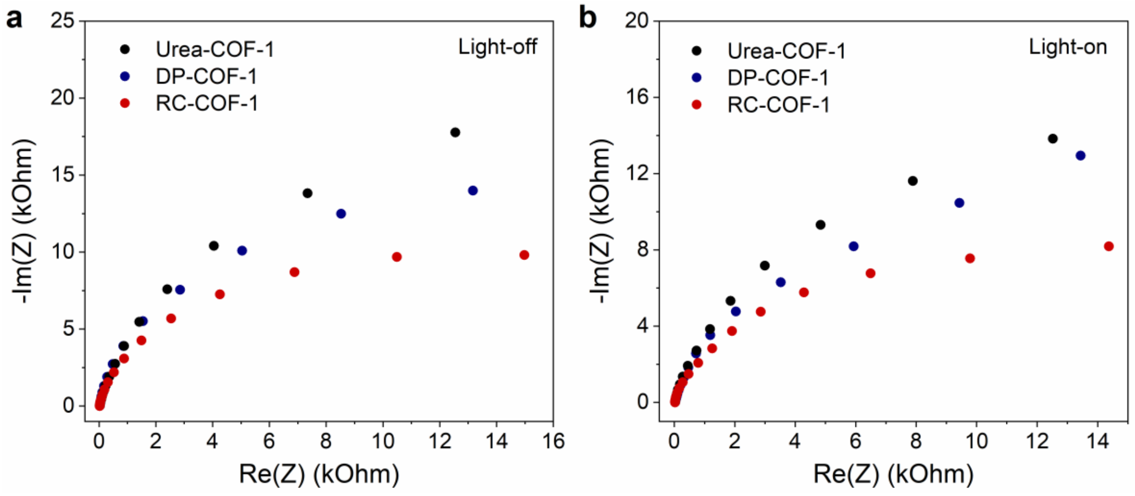

Supplementary Fig. 47 Nyquist plots from electrochemical impedance spectroscopy for RC-COF-1, DP-COF-1 and Urea-COF-1 in the dark (**a**) and under 300 W Xe lamp irradiation (*λ* > 420 nm) (**b**) with a bias potential of – 0.35 V *vs* Ag/AgCl reference electrode and Pt as a counter electrode.

Section 12 Photocatalytic hydrogen evolution experiments

We tested both DP-COF-1 and RC-COF-1 for sacrificial photocatalytic hydrogen evolution using platinum (Pt) as the co-catalyst. The optimized catalytic condition was 3 wt.% Pt loading with ascorbic acid as the sacrificial agent (Supplementary Fig. 48, 49). Urea-COF-1 showed an average hydrogen evolution rate (HER) of 2.08 mmol h^{-1} g^{-1} during 5 h visible-light photolysis (λ > 420 nm), and DP-COF-1 showed an average HER of 7.04 mmol h^{-1} g^{-1} (Extended Data Fig. 6c). RC-COF-1 exhibited an average HER of 27.98 mmol h^{-1} g^{-1}; that is, four times higher than DP-COF-1, despite having the same chemical formula. This activity was also twice as high as a state-of-art COF for this reaction, FS-COF[8], as measured under identical conditions (12.38 mmol h^{-1} g^{-1}). The mass-normalized HER for RC-COF-1 is among the highest reported for a COF photocatalyst (Supplementary Table 4). Control experiments were performed by removing either the light irradiation, the COF, or the sacrificial agent, and no hydrogen evolution was observed, supporting a sacrificial photocatalytic process mediated by the COF (Supplementary Fig. 50). The RC-COF-1 material also showed good reproducibility across different synthetic batches (Extended Data Fig. 6d), reinforcing the reliability of this reconstruction synthesis route.

The external quantum efficiencies (EQEs) of RC-COF-1 were estimated to be 6.39% at 420 nm, 5.92% at 490 nm, 5.20% at 515 nm, and 1.62% at 595 nm, respectively (Extended Data Fig. 6e). By comparison, DP-COF-1 exhibited a much-lower EQEs of 1.97%, 1.61%, 1.37%, and 0.54% at the same wavelengths. The quantum efficiencies for the reconstructed COF were three to four times higher than its directly polymerized analog, despite having the same nominal chemical composition. Again, we attribute this to the greatly enhanced crystallinity in RC-COF-1. The highly ordered donor and acceptor molecular columns could enable independent pathways for exciton migration and electron/hole transport, resulting in long-lived charge-separation states[9]. The long-term stability of RC-COF-1 under photocatalytic conditions was monitored for 60 hours, and no obvious decrease in activity was observed during this period (Extended Data Fig. 6f).

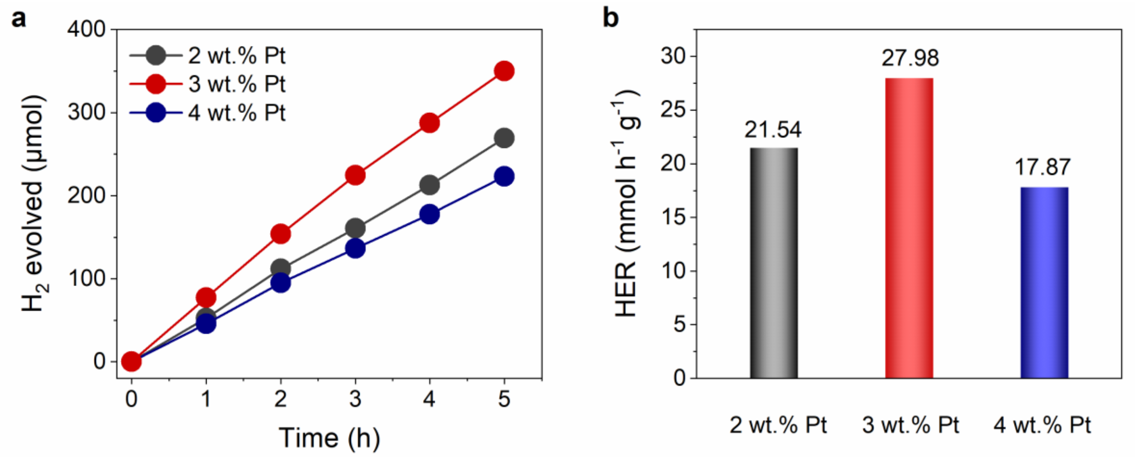

Supplementary Fig. 48 Time course of photocatalytic hydrogen evolution for RC-COF-1 under visible light irradiation (λ > 420 nm) with different Pt content as co-catalyst (2.5 mg COF and 0.1 M ascorbic acid as sacrificial agent) (**a**). Corresponding hydrogen evolution rates (**b**).

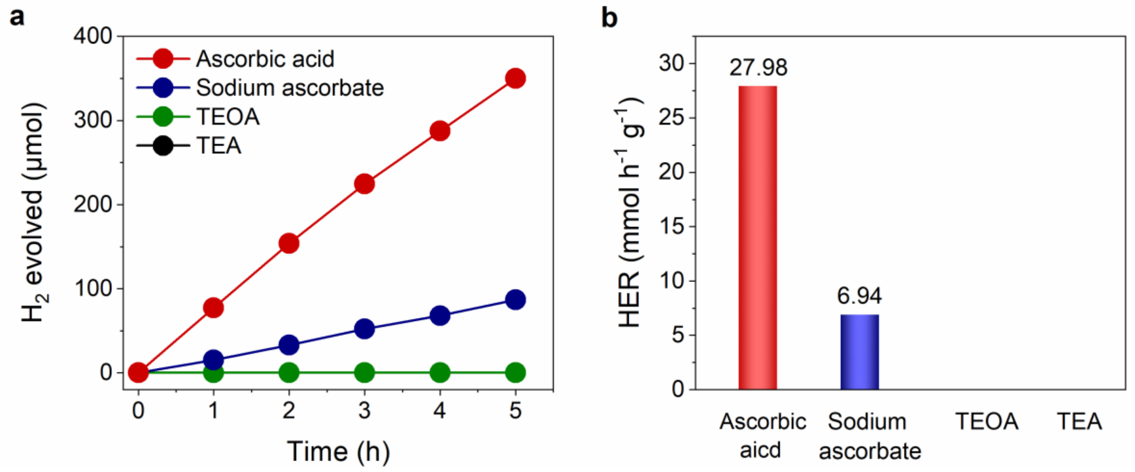

Supplementary Fig. 49 Time course of photocatalytic hydrogen evolution for RC-COF-1 under visible light irradiation (λ > 420 nm) with different sacrificial agents (2.5 mg COF and 3 wt.% Pt as co-catalyst) (**a**). Corresponding hydrogen evolution rates (**b**).

Supplementary Table 3 Photophysical properties and photocatalytic hydrogen evolution rates (HERs).

Photocatalyst	Irradiance	Band gap (eV)	HER (μmol h^{-1} g^{-1})	Degree of crystallinity
RC-COF-1	> 420 nm	1.87	27,980	High
DP-COF-1	> 420 nm	1.90	7,040	Moderate
Urea-COF-1	> 420 nm	2.12	2,080	High
FS-COF	> 420 nm	1.85	12,380	High
g-C_3N_4	> 295 nm	2.70	410	Moderate

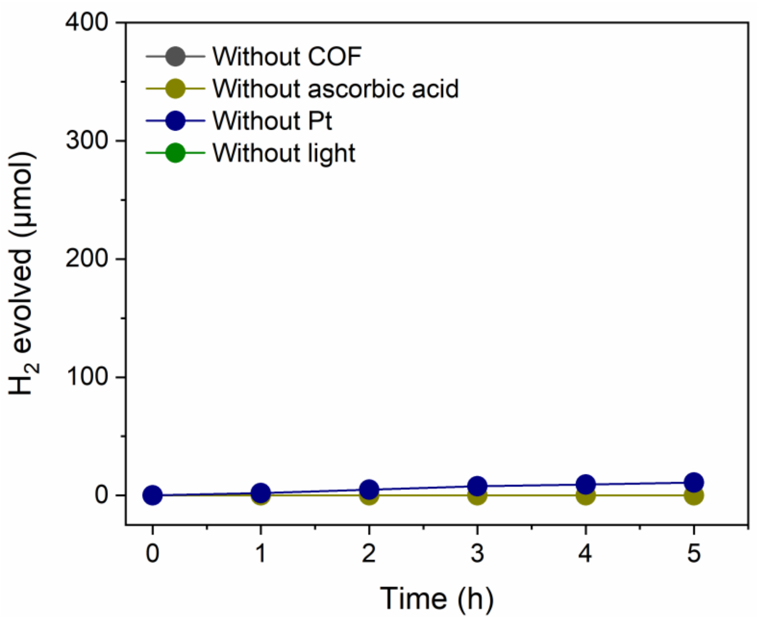

Supplementary Fig. 50 Control experiments of photocatalytic hydrogen evolution from water by sequentially removing one of the components (RC-COF-1, ascorbic acid, platinum, and light irradiation) from photocatalytic system, and no hydrogen evolution was observed, supporting a sacrificial photocatalytic process mediated by the COF.

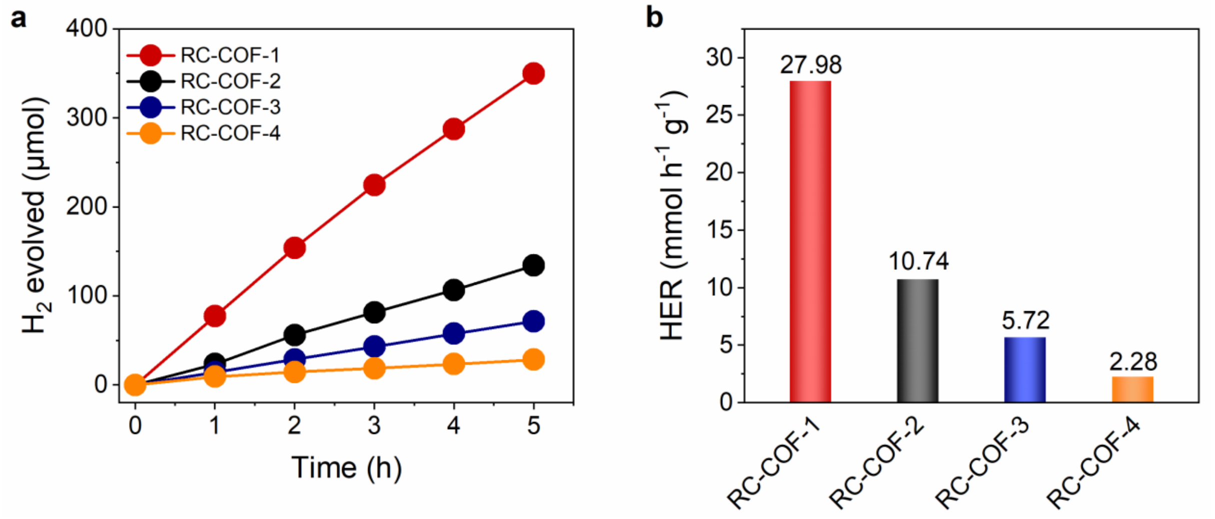

Supplementary Fig. 51 Time course of photocatalytic hydrogen evolution for RC-COFs under visible light irradiation (λ > 420 nm, 2.5 mg COF, 3 wt.% Pt as co-catalyst, and 0.1 M ascorbic acid as sacrificial agent) (**a**). Corresponding hydrogen evolution rates (**b**). RC-COF-1 exhibited the highest photocatalytic activity in this series of COFs.

Section 13 Post-photocatalysis characterization

The isolated material after photocatalytic experiments was characterized by PXRD, FTIR spectrum, UV-vis reflectance spectrum, and SEM (Supplementary Fig. 52–55, Extended Data Fig. 7), which showed retention of crystalline structure, demonstrating the high stability of RC-COF-1 as a photocatalyst. Notably, TEM images also showed RC-COF-1 crystallites that were decorated with photo-deposited Pt co-catalyst nanoparticles (Extended Data Fig. 7). The Pt nanoparticles had an average size of 2.5 nm and were uniformly dispersed on the rod-like crystallite of RC-COF-1. The uniform morphology of the reconstructed COF and the good Pt cocatalyst dispersion might also contribute to its enhanced activity.

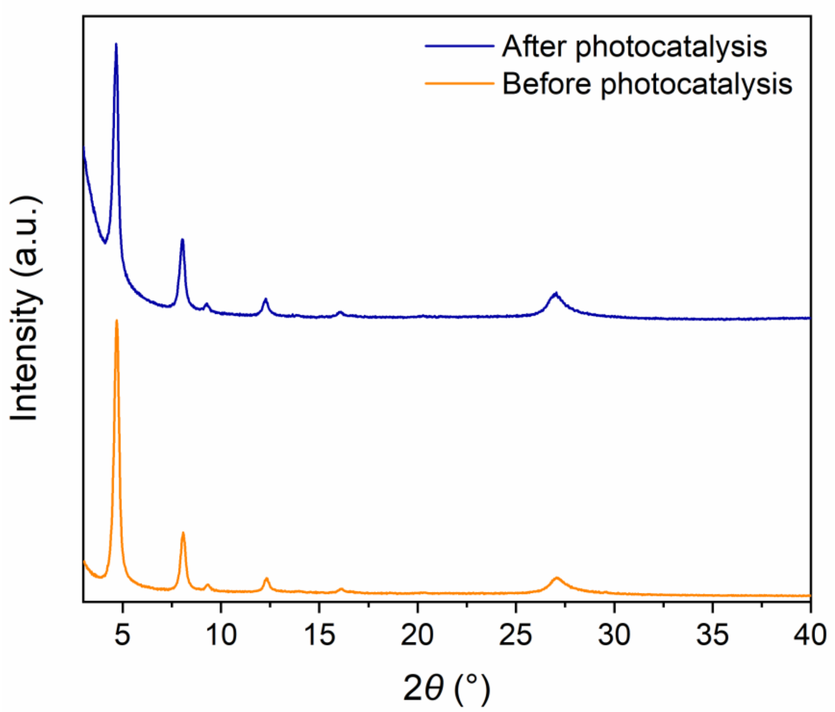

Supplementary Fig. 52 Experimental PXRD patterns for RC-COF-1 before (orange) and after 60 h photocatalysis (blue).

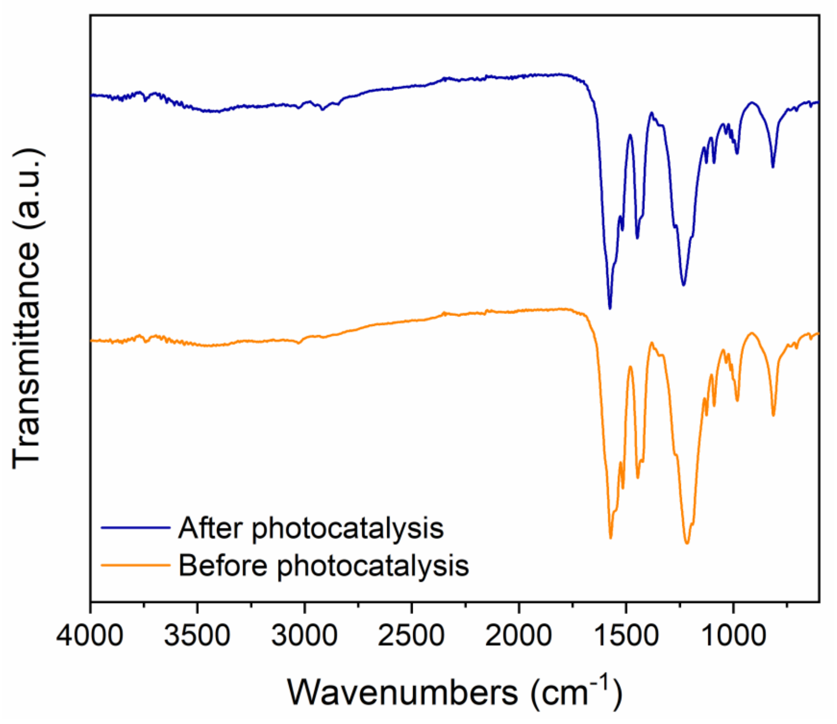

Supplementary Fig. 53 FTIR spectra for RC-COF-1 before (orange) and after 60 h photocatalysis (blue).

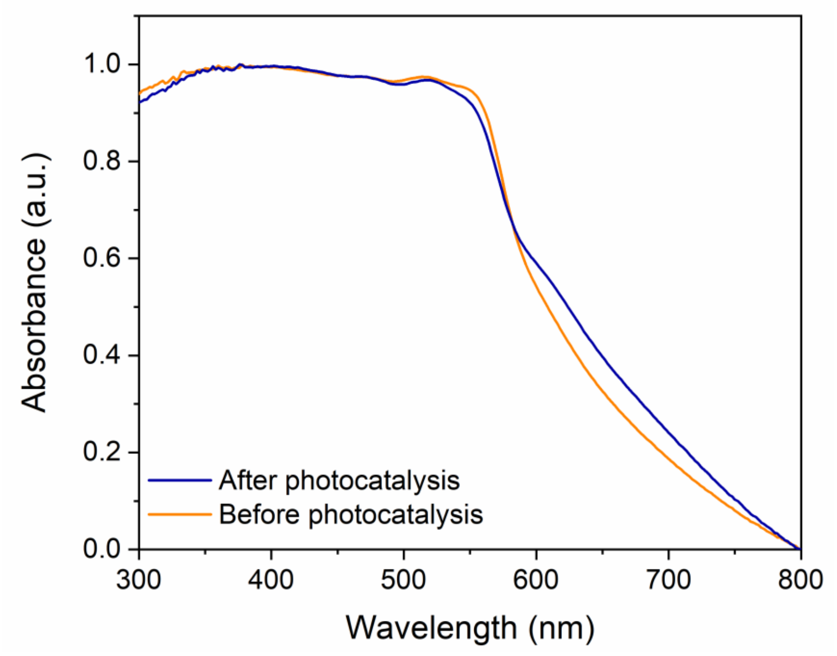

Supplementary Fig. 54 Solid-state UV-vis spectra for RC-COF-1 before (orange) and after 60 h photocatalysis (blue).

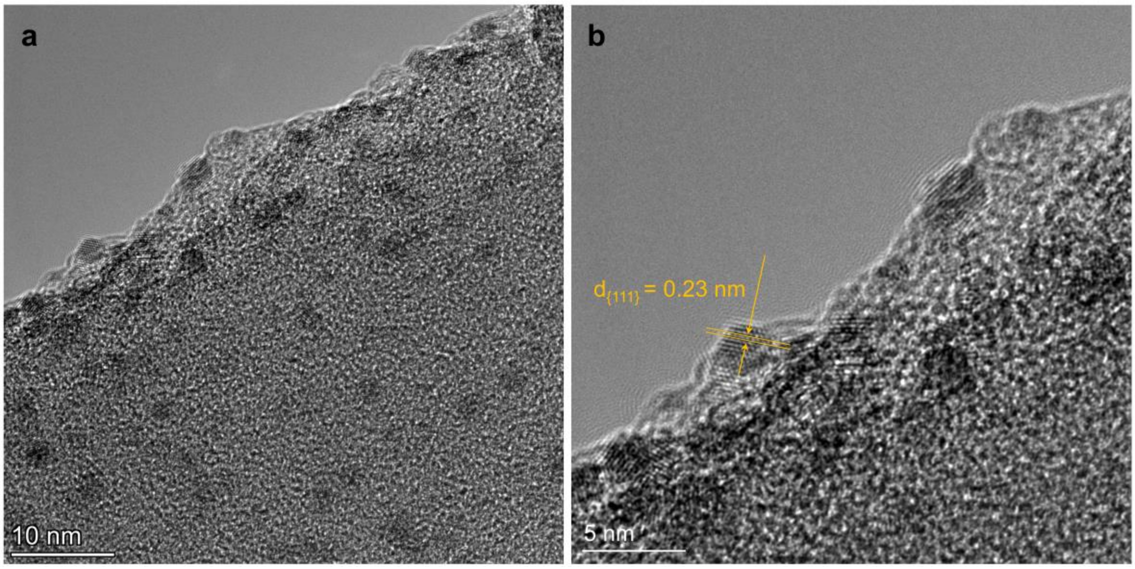

Supplementary Fig. 55 HRTEM image for Pt nanoparticles on the surface of RC-COF-1 (**a**). Enlarged HRTEM image shows {111} planes of Pt with *d*-spacing of 0.23 nm (**b**).

Section 14 Comparison of hydrogen evolution rates (HERs) for different COFs

Supplementary Table 4 A comparison of HERs for different COF photocatalysts reported in the literatures (hybrid and multi-component materials are not included here). We note here that HER and EQE can depend strongly on the precise experimental set-up (photoreactor geometry, scale, mixing, light intensity); as such, comparisons between data obtained in different laboratories, as here, should be used as a guide only. The FS-COF data, however, were collected in our laboratories on the same equipment and are therefore directly comparable. RC-COF-1 is much more active than the molecularly engineered FS-COF (Ref. 8), suggesting that the level of crystallinity in COFs might be at least as important for photochemical activity as their chemical composition.

COF	Co-catalyst	Sacrificial donor	Illumination	HER (μmol h^{-1} g^{-1})	EQE (%)	Ref.
TFPT-COF	Pt	TEOA	> 420 nm	1,970	2.2 (400 nm)	10
N_3-COF	Pt	TEOA	> 420 nm	1,703	0.44 (450 nm)	11
N_2-COF	chloro(pyridine) cobaloxime	TEOA	AM 1.5G	782	0.16 (400 nm)	12
A-TEBPY-COF	Pt	TEOA	AM 1.5G	98	n/d*	13
TP-BDDA	Pt	TEOA	> 395 nm	324	1.8 (520 nm)	5
FS-COF	Pt	Ascorbic acid	> 420 nm	10,100	3.2 (420 nm)	8
TpDTz	nickelthiolate hexameric	TEOA	AM 1.5G	941	0.2 (400 nm)	14
sp^2c-COF_{ERDN}	Pt	TEOA	> 420 nm	2,120	0.46 (420)<br>0.2 (578 nm)	15
g-$C_{40}N_3$-COF	Pt	TEOA	> 420 nm	4,120	4.84 (420 nm)<br>0.29 (578 nm)	16
g-$C_{18}N_3$-COF	Pt	Ascorbic acid	> 420 nm	292	1.06 (420 nm)	17
Pt-PVP-TPCOF	Pt	Ascorbic acid	> 420 nm	8,420	0.4 (475 nm)	18
Py-ClTP-BT-COF	Pt	Ascorbic acid	> 420 nm	8,875	8.45 (420 nm)	19
COF-alkene	Pt	TEOA	> 420 nm	2,330	6.7 (420 nm)	20
BtCOF150	Pt	TEOA	> 400 nm	750	0.2 (420 nm)	21
I-TST	Pt	TEOA	> 420 nm	125	n/d*	22
g-$C_{54}N_6$-COF	Pt	TEOA	> 420 nm	2,519	n/d*	23
NKCOF-108	Pt	Ascorbic acid	> 420 nm	8,000	2.96 (520 nm)	24
PyTz-COF	Pt	Ascorbic acid	AM 1.5G	2,072	n/d*	25
ZnPor-DETH-COF	Pt	TEOA	> 400 nm	413	0.063 (450 nm)	26
TtaTfa	Pt	Ascorbic acid	> 420 nm	20,700	1.43 (450 nm)	27
RC-COF-1	Pt	Ascorbic acid	> 420 nm	27,980	6.39 (420 nm)<br>1.62 (595 nm)	This work

*Note: n/d = not described

Section 15 Comparison of CO_2 uptake for different COFs

Supplementary Table 5 A comparison of CO_2 uptake capacities for different COFs reported in the literature.

COF	Measurement condition	BET surface area (m^2 g^{-1})	CO_2 uptake (cm^3 g^{-1})	Ref.
3D-CageCOF-1	273 K, 1 bar	1,040	103	28
3D-TPB-COF-H	273 K, 1 bar	1,050	90	29
3D-TPB-COF-Me	273 K, 1 bar	950	90	29
3D-TPB-COF-F	273 K, 1 bar	850	90	29
3D-ionic-COF-1	273 K, 1 bar	966	82	30
3D-ionic-COF-2	273 K, 1 bar	880	68	30
3D-TPE-COF	273 K, 1 bar	1084	72	31
CD-COF-PPZ	273 K, 1 bar	494	62	32
LZU-301	273 K, 1 bar	848	59	33
3D-Py-COF	273 K, 1 bar	1,290	78	34
DL-COF-1	273 K, 1 bar	2,259	136	35
DL-COF-2	273 K, 1 bar	2,071	111	35
Cage-COF-1	273 K, 1 bar	1,237	44	36
Cage-COF-2	273 K, 1 bar	667	37	36
TpPa-COF (MW)	273 K, 1 bar	725	111	37
TPE-COF-II	273 K, 1 bar	2,168	119	38
3D-ceq-COF	273 K, 1 bar	1,149	91	39
JUC-568	273 K, 1 bar	1,254	98	40
RC-COF-1	273 K, 1 bar	1,712	147	This work

Section 16 Liquid NMR spectra

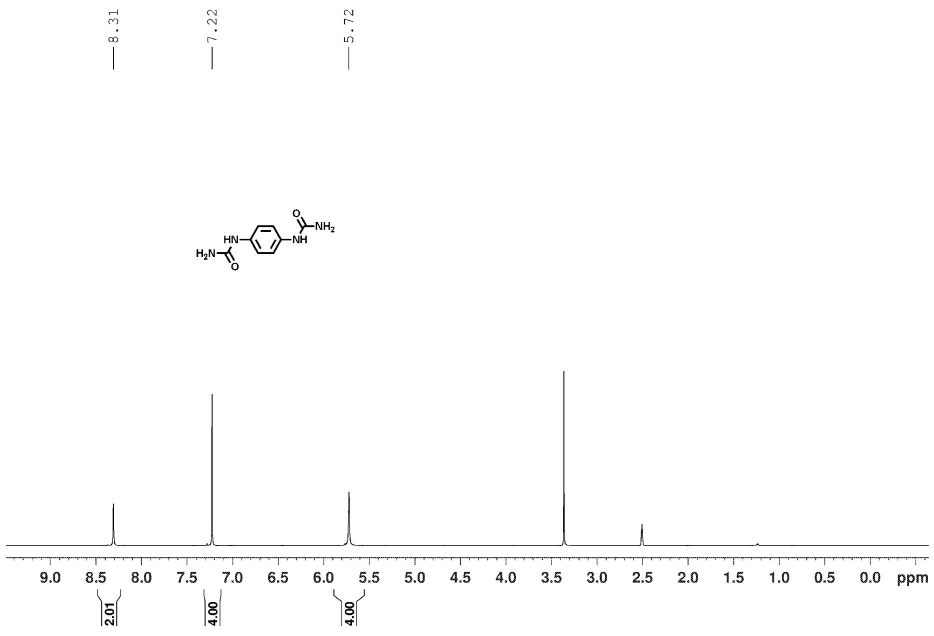

Supplementary Fig. 56 Liquid ^{1}H NMR spectrum of 1,1'-(1,4-phenylene)diurea in DMSO-d_6.

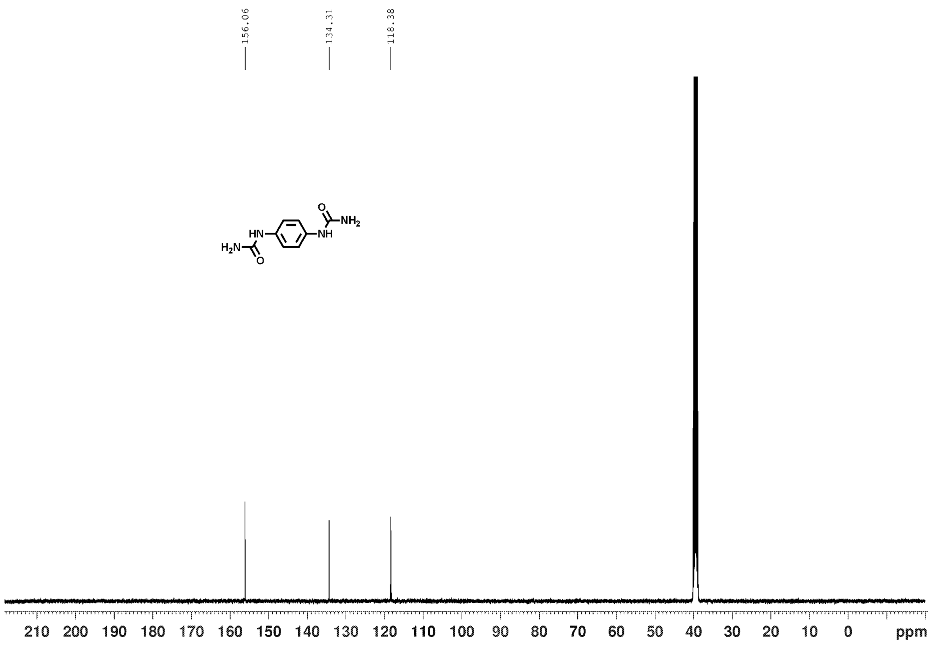

Supplementary Fig. 57 Liquid ^{13}C NMR spectrum of 1,1'-(1,4-phenylene)diurea in DMSO-d_6.

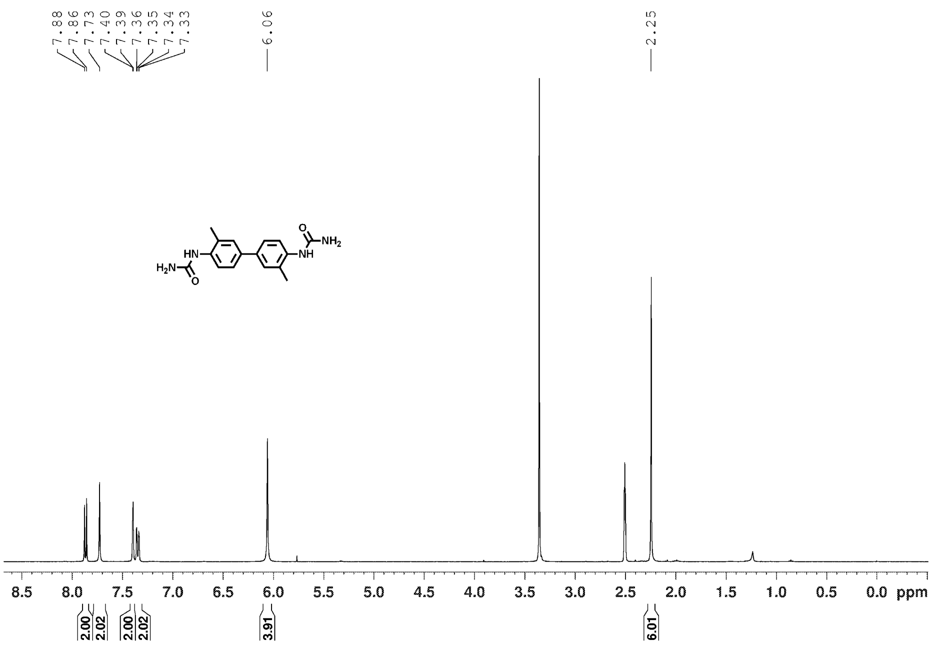

Supplementary Fig. 58 Liquid ^{1}H NMR spectrum of 1,1'-(3,3'-Dimethyl-[1,1'-biphenyl]-4,4'-diyl)diurea in DMSO-d_6.

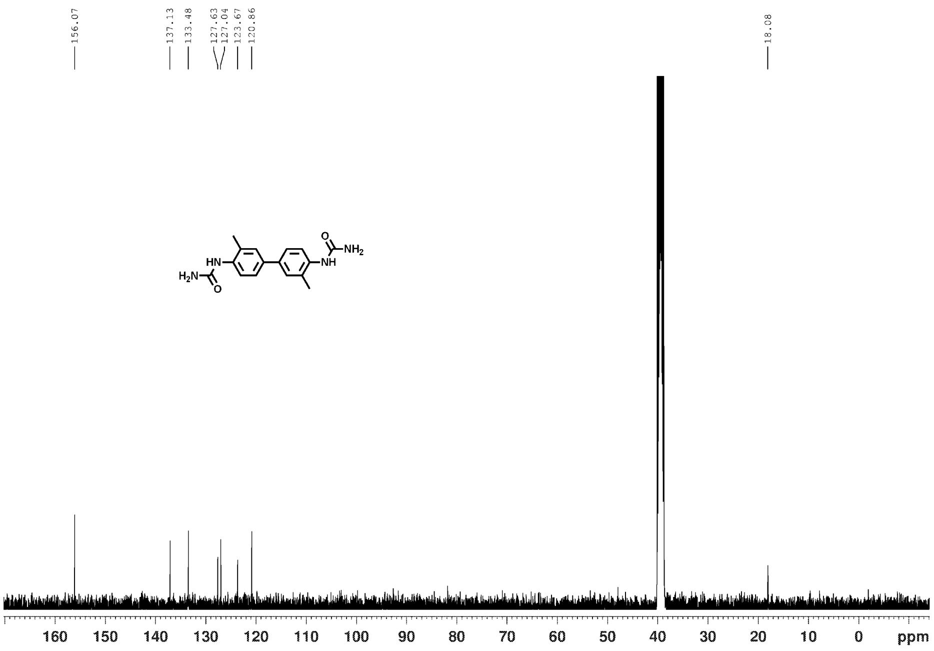

Supplementary Fig. 59 Liquid ^{13}C NMR spectrum of 1,1'-(3,3'-Dimethyl-[1,1'-biphenyl]-4,4'-diyl)diurea in DMSO-d_6.

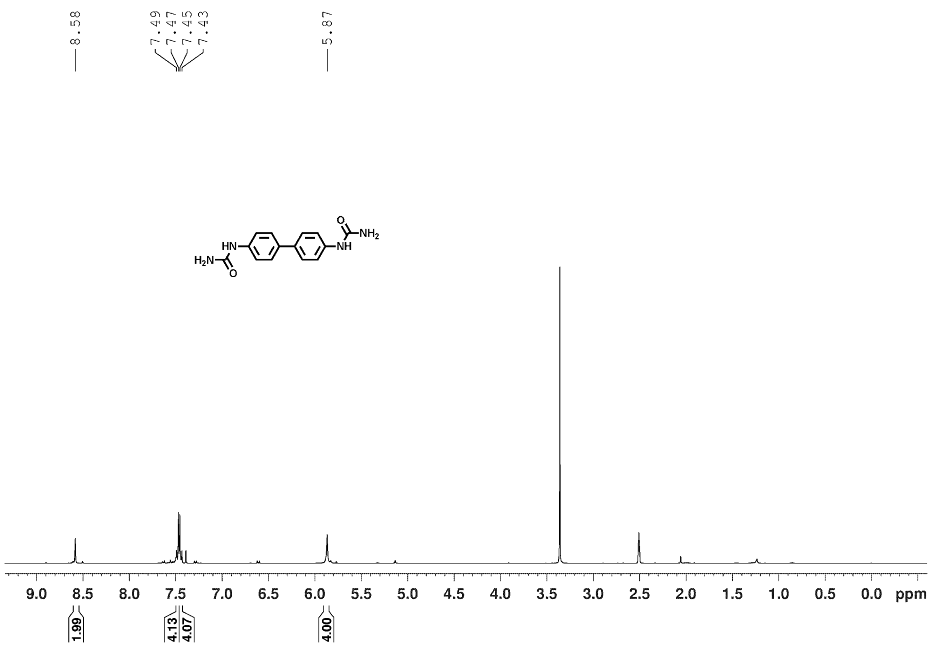

Supplementary Fig. 60 Liquid ^{1}H NMR spectrum of 1,1'-([1,1'-biphenyl]-4,4'-diyl)diurea in DMSO-d_6.

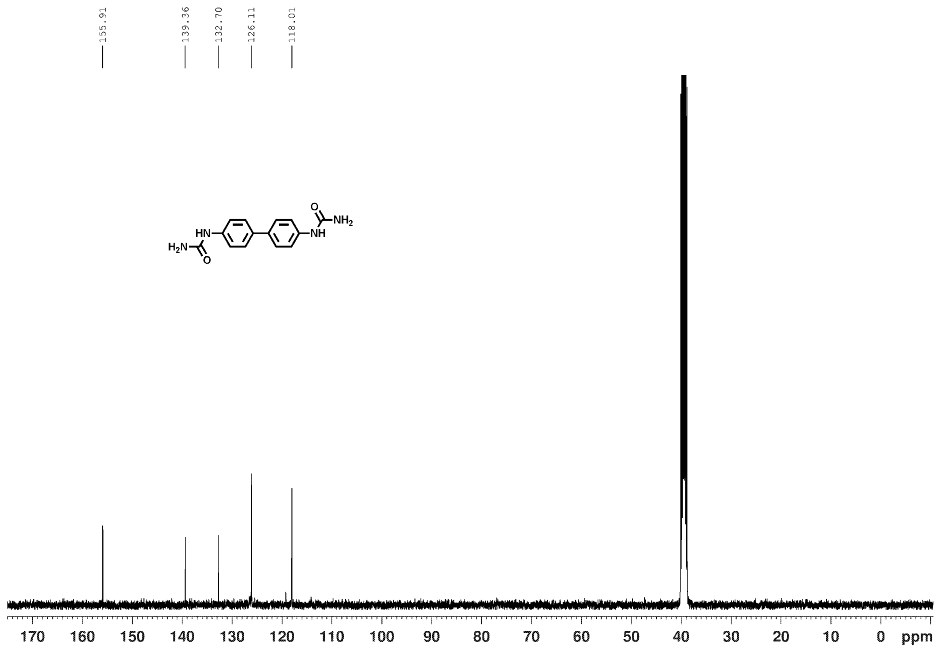

Supplementary Fig. 61 Liquid ^{13}C NMR spectrum of 1,1'-([1,1'-biphenyl]-4,4'-diyl)diurea in DMSO-d_6.

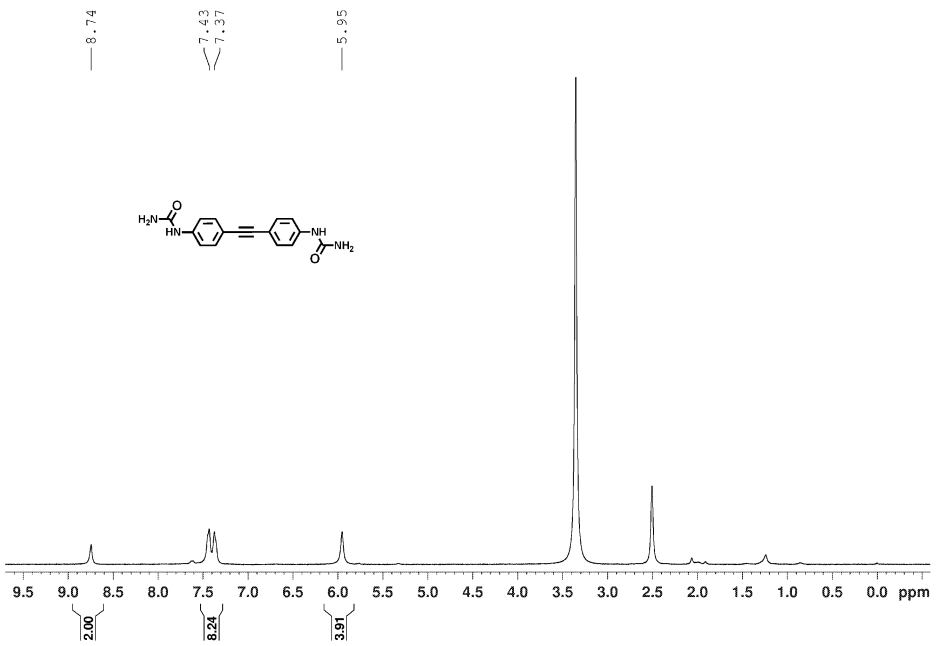

Supplementary Fig. 62 Liquid ^{1}H NMR spectrum of 1,1'-(ethyne-1,2-diylbis(4,1-phenylene))diurea in DMSO-d_6.

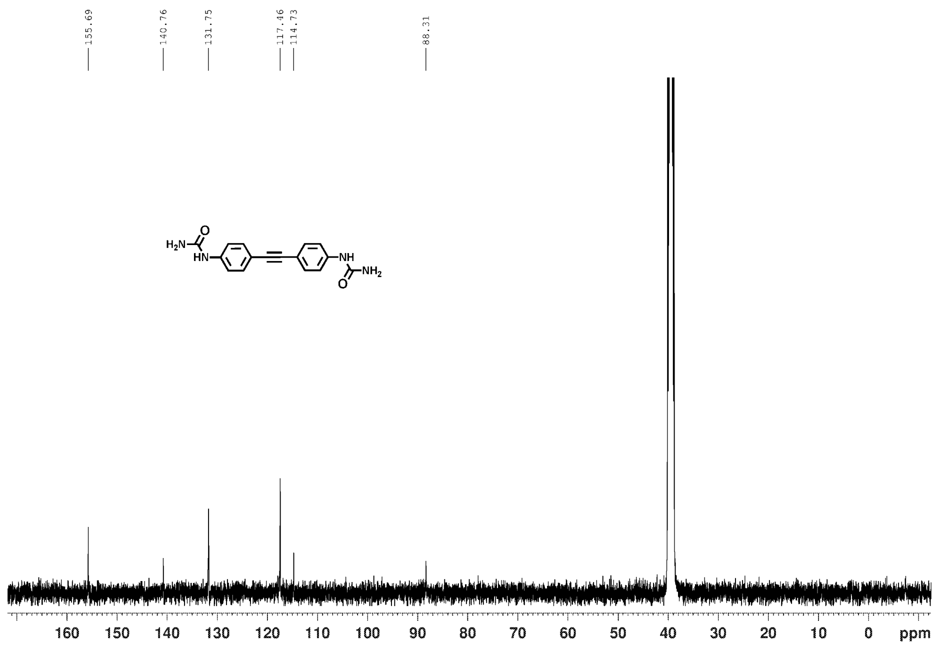

Supplementary Fig. 63 Liquid ^{13}C NMR spectrum of 1,1'-(ethyne-1,2-diylbis(4,1-phenylene))diurea in DMSO-d_6.

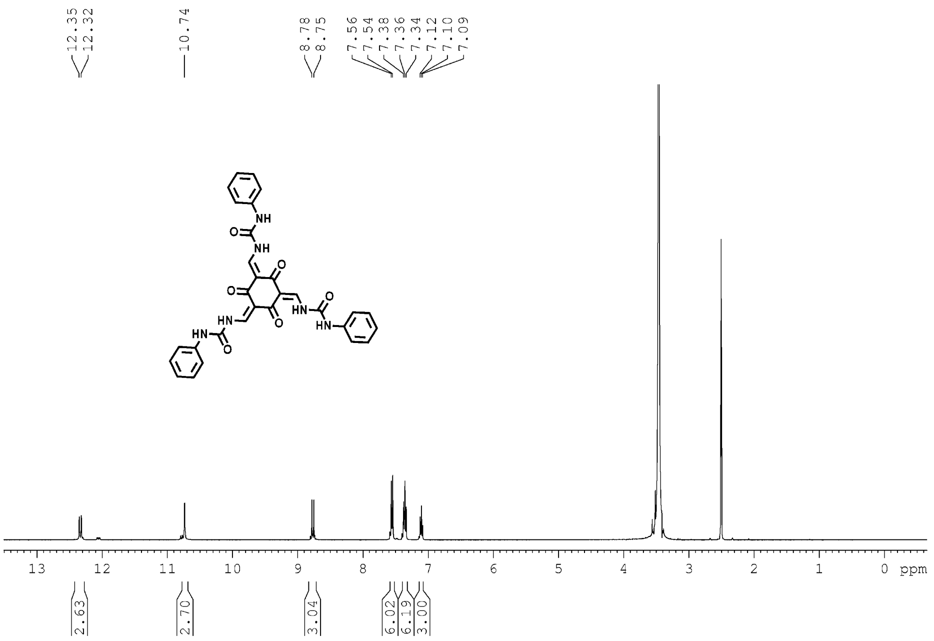

Supplementary Fig. 64 Liquid ^{1}H NMR spectrum of the urea-linked model compound in DMSO-d_6.

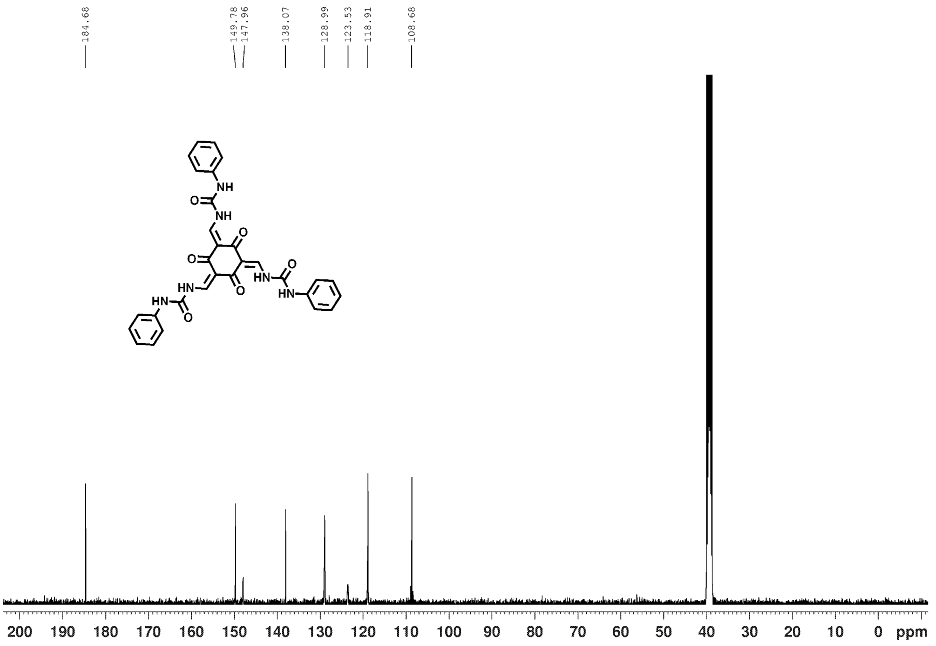

Supplementary Fig. 65 Liquid ^{13}C NMR spectrum of the urea-linked model compound in DMSO-d_6.

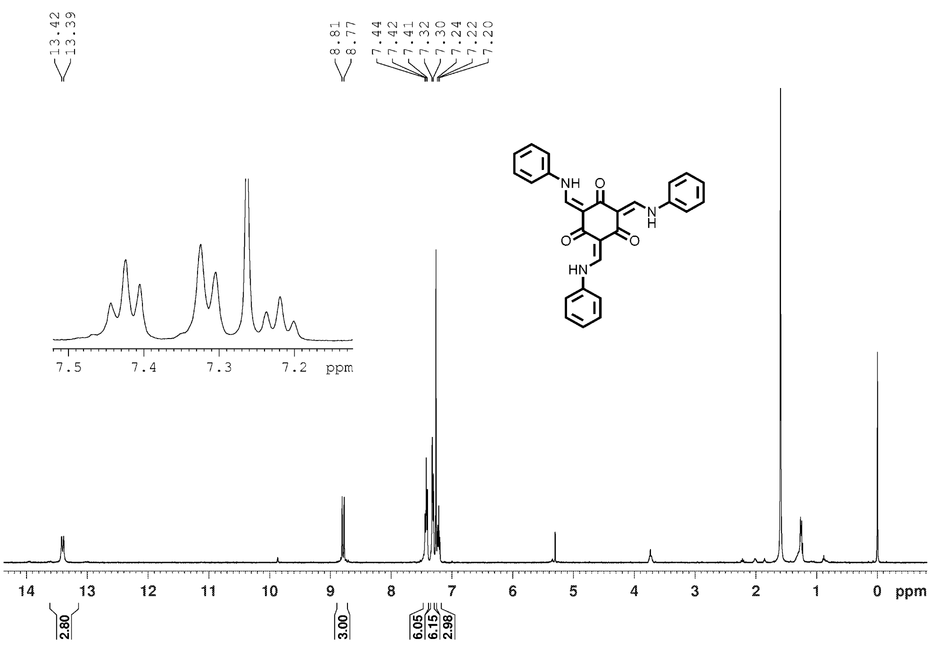

Supplementary Fig. 66 Liquid ^{1}H NMR spectrum of the β-ketoenamine model compound in $CDCl_3$ which was synthesized by direct condensation.

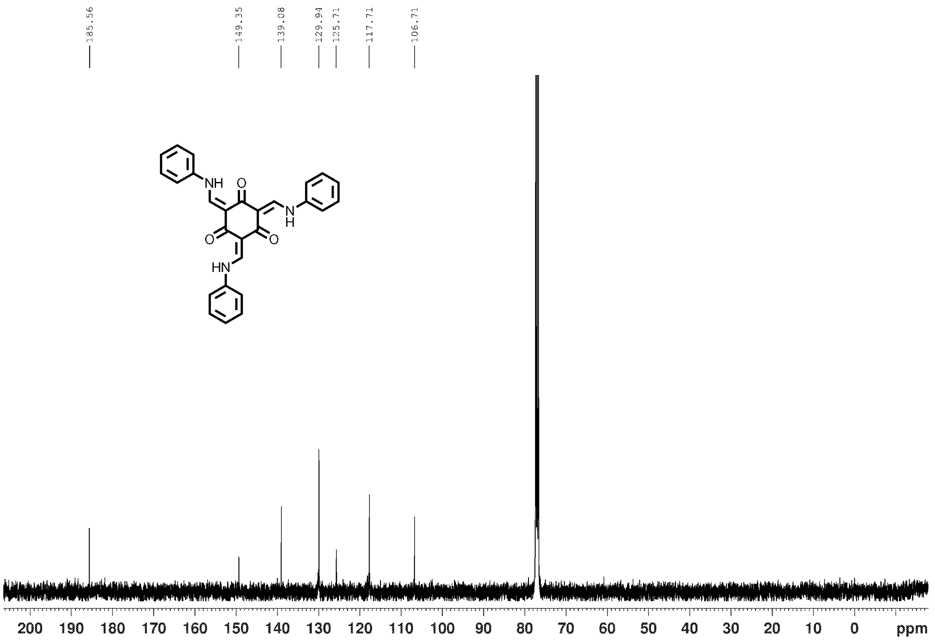

Supplementary Fig. 67 Liquid ^{13}C NMR spectrum of the β-ketoenamine model compound in $CDCl_3$ which was synthesized by direct condensation.

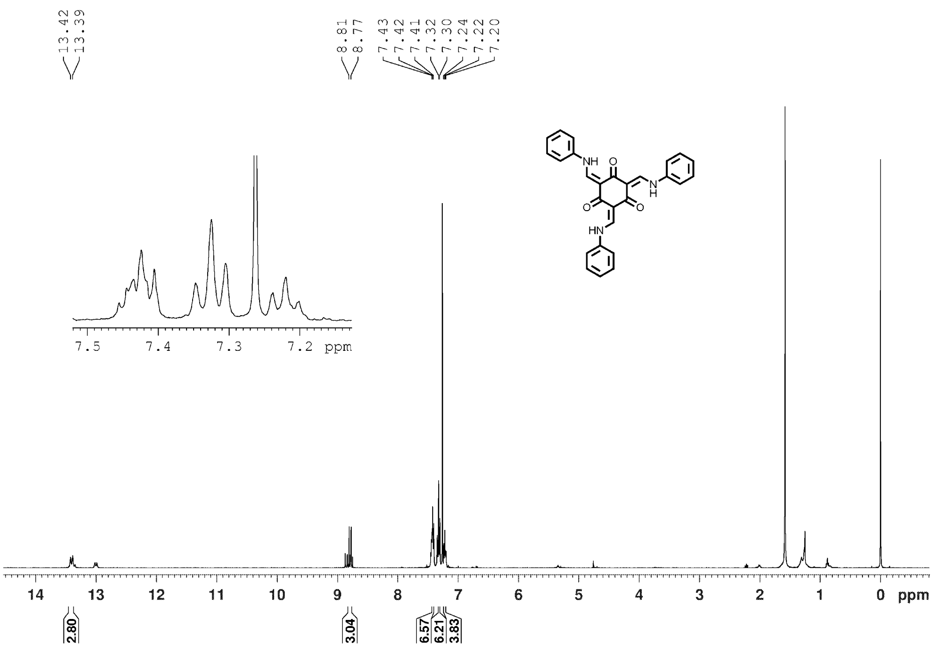

Supplementary Fig. 68 Liquid ^{1}H NMR spectrum of the *β*-ketoenamine model compound in $CDCl_3$ which was synthesized by hydrothermal treatment of urea model compound.

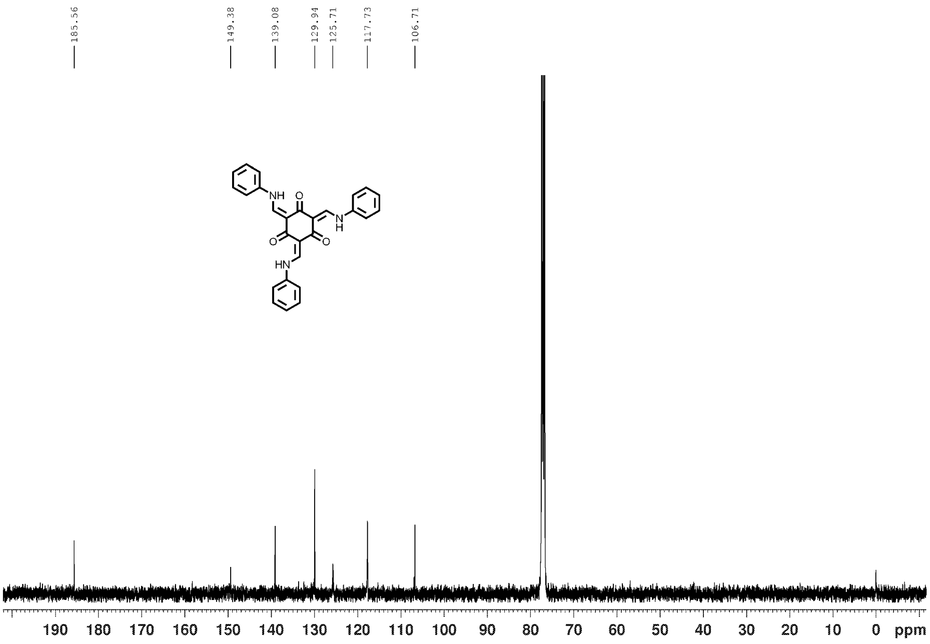

Supplementary Fig. 69 Liquid ^{13}C NMR spectrum of the *β*-ketoenamine model compound in $CDCl_3$ which was synthesized by hydrothermal treatment of urea model compound.

Section 17 HRMS spectra

Elemental Composition Report **Page 1**

Single Mass Analysis
Tolerance = 5.0 mDa / DBE: min = -1.5, max = 50.0
Element prediction: Off
Number of isotope peaks used for i-FIT = 3

Monoisotopic Mass, Even Electron Ions
31 formula(e) evaluated with 1 results within limits (up to 50 best isotopic matches for each mass)
Elements Used:
C: 0-8 H: 0-99 N: 0-4 O: 0-2 Na: 0-1
WH-ZHU
ZW-ZWW-1 76 (1.641) Cm (70:76) 1: TOF MS ES+ 6.17e+003

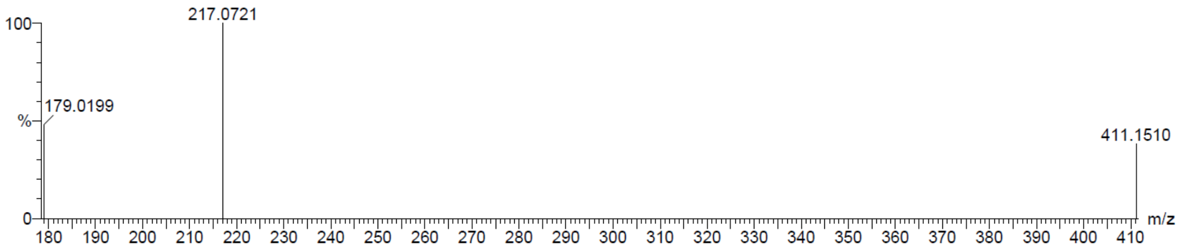

Minimum:				-1.5			
Maximum:		5.0	10.0	50.0			
Mass	Calc. Mass	mDa	PPM	DBE	i-FIT	i-FIT (Norm)	Formula
217.0721	217.0701	2.0	9.2	5.5	27.7	0.0	C8 H10 N4 O2 Na

Supplementary Fig. 70 HRMS spectrum of 1,1'-(1,4-phenylene)diurea.

Elemental Composition Report **Page 1**

Single Mass Analysis
Tolerance = 5.0 mDa / DBE: min = -1.5, max = 50.0
Element prediction: Off
Number of isotope peaks used for i-FIT = 3

Monoisotopic Mass, Even Electron Ions
19 formula(e) evaluated with 1 results within limits (up to 50 best isotopic matches for each mass)
Elements Used:
C: 0-16 H: 0-99 N: 0-4 O: 0-2
WH-ZHU
ZW-ZWW-2 140 (3.051) Cm (140:141) 1: TOF MS ES- 4.76e+003

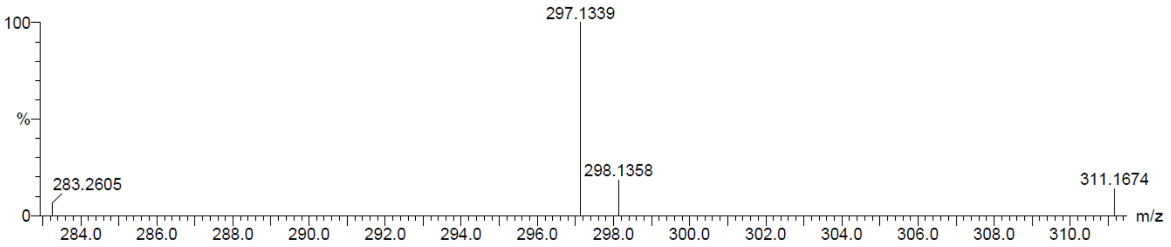

Minimum:				-1.5			
Maximum:		5.0	10.0	50.0			
Mass	Calc. Mass	mDa	PPM	DBE	i-FIT	i-FIT (Norm)	Formula
297.1339	297.1352	-1.3	-4.4	10.5	23.3	0.0	C16 H17 N4 O2

Supplementary Fig. 71 HRMS spectrum of 1,1'-(3,3'-Dimethyl-[1,1'-biphenyl]-4,4'-diyl)diurea.

Elemental Composition Report

Page 1

Single Mass Analysis

Tolerance = 5.0 mDa / DBE: min = -1.5, max = 50.0
Element prediction: Off
Number of isotope peaks used for i-FIT = 3

Monoisotopic Mass, Even Electron Ions
19 formula(e) evaluated with 1 results within limits (up to 50 best isotopic matches for each mass)
Elements Used:
C: 0-14 H: 0-99 N: 0-4 O: 0-2
WH-ZHU
ZW-ZWW-3 123 (2.682) Cm (123:125)

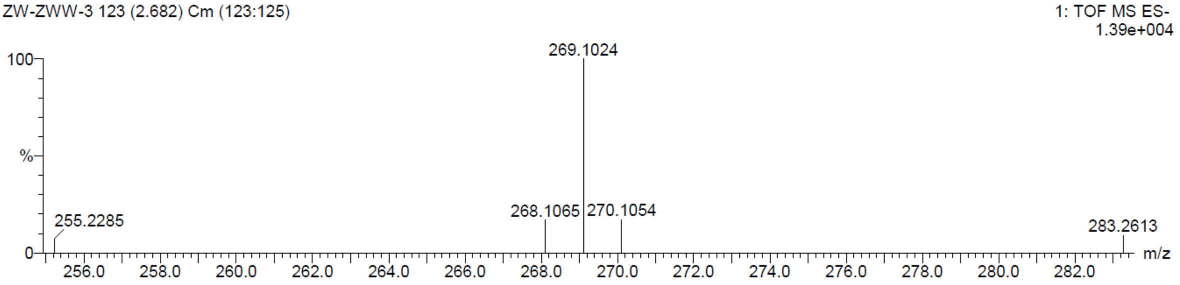

Minimum:				-1.5			
Maximum:		5.0	10.0	50.0			
Mass	Calc. Mass	mDa	PPM	DBE	i-FIT	i-FIT (Norm)	Formula
269.1024	269.1039	-1.5	-5.6	10.5	25.2	0.0	C14 H13 N4 O2

Supplementary Fig. 72 HRMS spectrum of 1,1'-([1,1'-biphenyl]-4,4'-diyl)diurea.

Elemental Composition Report

Page 1

Single Mass Analysis

Tolerance = 5.0 mDa / DBE: min = -1.5, max = 50.0
Element prediction: Off
Number of isotope peaks used for i-FIT = 3

Monoisotopic Mass, Even Electron Ions
19 formula(e) evaluated with 1 results within limits (up to 50 best isotopic matches for each mass)
Elements Used:
C: 0-16 H: 0-99 N: 0-4 O: 0-2
WH-ZHU
ZW-ZWW-4 18 (0.372) Cm (18)

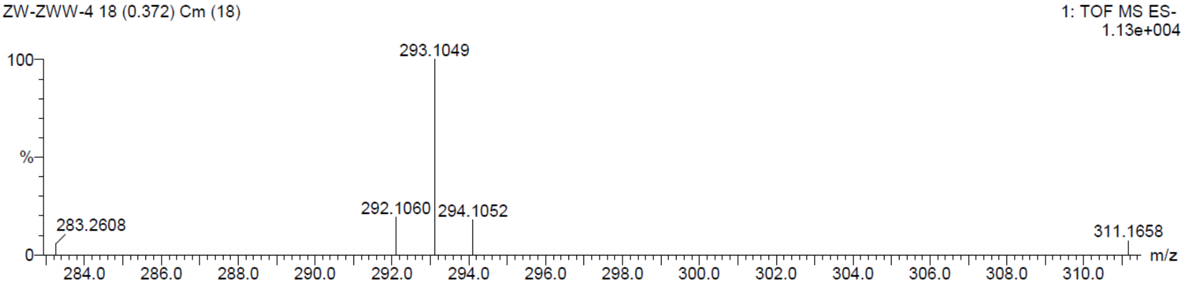

Minimum:				-1.5			
Maximum:		5.0	10.0	50.0			
Mass	Calc. Mass	mDa	PPM	DBE	i-FIT	i-FIT (Norm)	Formula
293.1049	293.1039	1.0	3.4	12.5	28.9	0.0	C16 H13 N4 O2

Supplementary Fig. 73 HRMS spectrum of 1,1'-(ethyne-1,2-diylbis(4,1-phenylene))diurea.

Elemental Composition Report **Page 1**

Single Mass Analysis
Tolerance = 5.0 mDa / DBE: min = -1.5, max = 50.0
Element prediction: Off
Number of isotope peaks used for i-FIT = 3

Monoisotopic Mass, Even Electron Ions
59 formula(e) evaluated with 1 results within limits (up to 50 best isotopic matches for each mass)
Elements Used:
C: 0-30 H: 0-99 N: 0-6 O: 0-6
WH-ZHU
ZW-ZWW-5 22 (0.460) Cm (22)

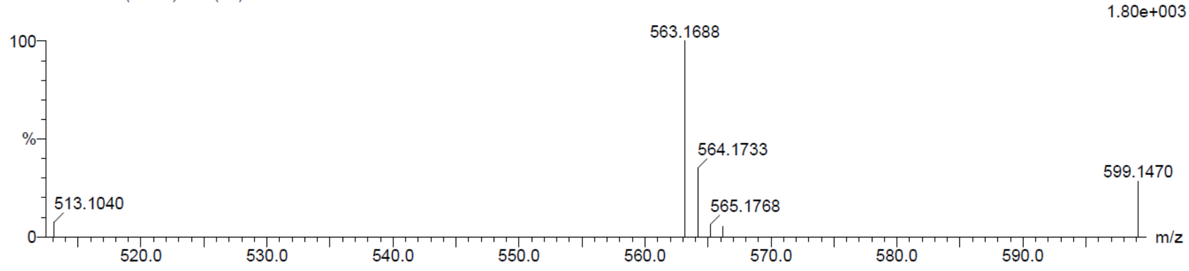

Minimum:				-1.5			
Maximum:		5.0	10.0	50.0			
Mass	Calc. Mass	mDa	PPM	DBE	i-FIT	i-FIT (Norm)	Formula
563.1688	563.1679	0.9	1.6	22.5	8.6	0.0	C30 H23 N6 O6

Supplementary Fig. 74 HRMS spectrum of urea-linked model compound ((2,4,6-trioxocyclohexane-1,3,5-triylidene)tris(methanylylidene)tris(3-phenylurea)).

Elemental Composition Report **Page 1**

Single Mass Analysis
Tolerance = 5.0 mDa / DBE: min = -1.5, max = 50.0
Element prediction: Off
Number of isotope peaks used for i-FIT = 3

Monoisotopic Mass, Even Electron Ions
26 formula(e) evaluated with 1 results within limits (up to 50 best isotopic matches for each mass)
Elements Used:
C: 0-27 H: 0-99 N: 0-3 O: 0-3
WH-ZHU
ZW-ZWW-6 14 (0.317) Cm (14)

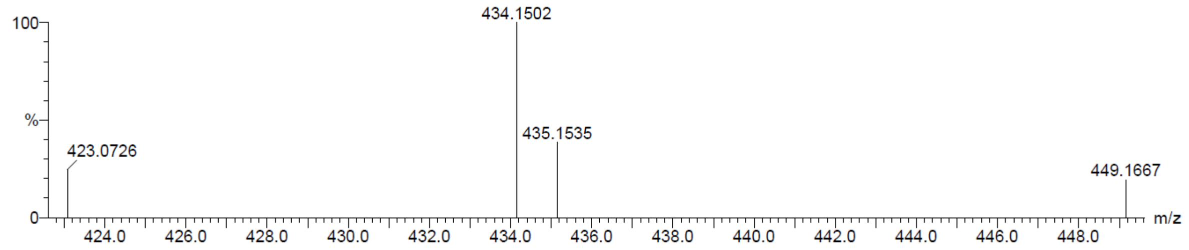

Minimum:				-1.5			
Maximum:		5.0	10.0	50.0			
Mass	Calc. Mass	mDa	PPM	DBE	i-FIT	i-FIT (Norm)	Formula
434.1502	434.1505	-0.3	-0.7	19.5	17.1	0.0	C27 H20 N3 O3

Supplementary Fig. 75 HRMS spectrum of *β*-ketoenamine model compound (2,4,6-tris((phenylamino)methylene) cyclohexane-1,3,5-trione) which was synthesized by direct condensation.

Elemental Composition Report **Page 1**

Single Mass Analysis
Tolerance = 5.0 mDa / DBE: min = -1.5, max = 50.0
Element prediction: Off
Number of isotope peaks used for i-FIT = 3

Monoisotopic Mass, Even Electron Ions
26 formula(e) evaluated with 1 results within limits (up to 50 best isotopic matches for each mass)
Elements Used:
C: 0-27 H: 0-99 N: 0-3 O: 0-3
WH-ZHU
ZW-ZWW-7 26 (0.549) Cm (25:26) 1: TOF MS ES- 2.10e+002

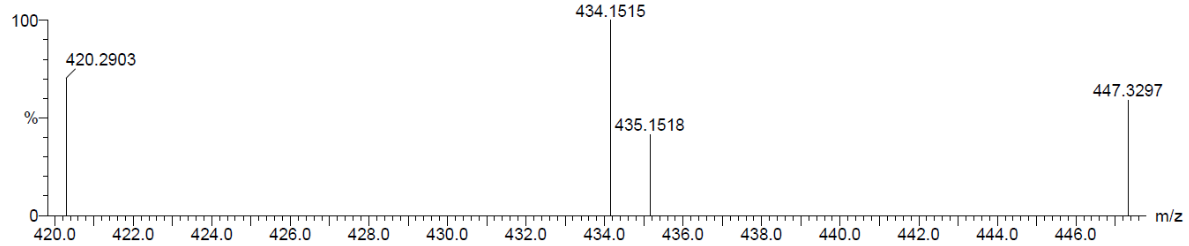

Minimum:				-1.5			
Maximum:		5.0	10.0	50.0			
Mass	Calc. Mass	mDa	PPM	DBE	i-FIT	i-FIT (Norm)	Formula
434.1515	434.1505	1.0	2.3	19.5	15.5	0.0	C27 H20 N3 O3

Supplementary Fig. 76 HRMS spectrum of *β*-ketoenamine model compound (2,4,6-tris((phenylamino)methylene) cyclohexane-1,3,5-trione) which was synthesized by hydrothermal treatment of urea model compound.

References

1. Zhao, C. et al. Urea-linked covalent organic frameworks. *J. Am. Chem. Soc.* **140**, 16438-16441 (2018).
2. Kandambeth, S. et al. Construction of crystalline 2D covalent organic frameworks with remarkable chemical (acid/base) stability via a combined reversible and irreversible route. *J. Am. Chem. Soc.* **134**, 19524-19527 (2012).
3. Chandra, S. et al. Chemically stable multilayered covalent organic nanosheets from covalent organic frameworks via mechanical delamination. *J. Am. Chem. Soc.* **135**, 17853-17861 (2013).
4. Biswal, B. P. et al. Mechanochemical synthesis of chemically stable isoreticular covalent organic frameworks. *J. Am. Chem. Soc.* **135**, 5328-5331 (2013).
5. Pachfule, P. et al. Diacetylene functionalized covalent organic framework (COF) for photocatalytic hydrogen generation. *J. Am. Chem. Soc.* **140**, 1423-1427 (2018).
6. Ming, J. et al. Hot π-electron tunneling of metal–insulator–COF nanostructures for efficient hydrogen production. *Angew. Chem. Int. Ed.* **58**, 18290-18294 (2019).
7. Ou, H., Chen, X., Lin, L., Fang, Y. & Wang, X. Biomimetic donor–acceptor motifs in conjugated polymers for promoting exciton splitting and charge separation. *Angew. Chem. Int. Ed.* **57**, 8729-8733 (2018).
8. Wang, X. et al. Sulfone-containing covalent organic frameworks for photocatalytic hydrogen evolution from water. *Nat. Chem.* **10**, 1180-1189 (2018).
9. Jin, S. et al. Creation of superheterojunction polymers via direct polycondensation: segregated and bicontinuous donor-acceptor π-columnar arrays in covalent organic frameworks for long-lived charge separation. *J. Am. Chem. Soc.* **137**, 7817-7827 (2015).
10. Stegbauer, L., Schwinghammer, K. & Lotsch, B. V. A hydrazone-based covalent organic framework for photocatalytic hydrogen production. *Chem. Sci.* **5**, 2789-2793 (2014).
11. Vyas, V. S. et al. A tunable azine covalent organic framework platform for visible light-induced hydrogen generation. *Nat. Commun.* **6**, 8508 (2015).
12. Banerjee, T. et al. Single-site photocatalytic H_2 evolution from covalent organic frameworks with molecular cobaloxime co-catalysts. *J. Am. Chem. Soc.* **139**, 16228-16234 (2017).
13. Stegbauer, L. et al. Photocatalysis: tailor-made photoconductive pyrene-based covalent organic frameworks for visible-light driven hydrogen generation. *Adv. Energy Mater.* **8**, 1870107 (2018).
14. Biswal, B. P. et al. Sustained solar H_2 evolution from a thiazolo[5,4-d]thiazole-bridged covalent organic framework and nickel-thiolate cluster in water. *J. Am. Chem. Soc.* **141**, 11082-11092 (2019).
15. Jin, E. et al. 2D sp^2 carbon-conjugated covalent organic frameworks for photocatalytic hydrogen production from water. *Chem* **5**, 1632-1647 (2019).
16. Bi, S. et al. Two-dimensional semiconducting covalent organic frameworks via condensation at arylmethyl carbon atoms. *Nat. Commun.* **10**, 2467 (2019).
17. Wei, S. et al. Semiconducting 2D triazine-cored covalent organic frameworks with unsubstituted olefin linkages. *J. Am. Chem. Soc.* **141**, 14272-14279 (2019). doi:10.1021/jacs.9b06219
18. Ming, J. et al. Hot π-electron tunneling of metal-insulator-COF nanostructures for efficient hydrogen production. *Angew. Chem. Int. Ed.* **58**, 18290-18294 (2019).
19. Chen, W. et al. Modulating benzothiadiazole-based covalent organic frameworks via halogenation for enhanced photocatalytic water splitting. *Angew. Chem. Int. Ed.* **59**, 16902-16909 (2020).
20. Mo, C. et al. Alkene-linked covalent organic frameworks boosting photocatalytic hydrogen evolution by efficient charge separation and transfer in the presence of sacrificial electron donors. *Adv. Sci.* **7**, 1902988 (2020).

21. Ghosh, S. et al. Identification of prime factors to maximize the photocatalytic hydrogen evolution of covalent organic frameworks. *J. Am. Chem. Soc.* **142**, 9752-9762 (2020).
22. Wan, Y., Wang, L., Xu, H., Wu, X. & Yang, J. A simple molecular design strategy for two-dimensional covalent organic framework capable of visible-light-driven water splitting. *J. Am. Chem. Soc.* **142**, 4508-4516 (2020).
23. Xu, J. et al. Vinylene-linked covalent organic frameworks (COFs) with symmetry-tuned polarity and photocatalytic activity. *Angew. Chem. Int. Ed.* **59**, 23845-23853 (2020).
24. Zhao, Z. et al. Fabrication of robust covalent organic frameworks for enhanced visible-light-driven H_2 evolution. *ACS Catal.* **11**, 2098-2107 (2021).
25. Li, W. et al. Thiazolo[5,4-d]thiazole-based donor-acceptor covalent organic framework for sunlight-driven hydrogen evolution. *Angew. Chem. Int. Ed.* **60**, 1869-1874 (2021).
26. Chen, R. et al. Rational design of isostructural 2D porphyrin-based covalent organic frameworks for tunable photocatalytic hydrogen evolution. *Nat. Commun.* **12**, 1354 (2021).
27. Yang, J. et al. Protonated imine-linked covalent organic frameworks for photocatalytic hydrogen evolution. *Angew. Chem. Int. Ed.* **60**, 19797-19803 (2021).
28. Zhu, Q. et al. 3D cage COFs: A dynamic three-dimensional covalent organic framework with high-connectivity organic cage nodes. *J. Am. Chem. Soc.* **142**, 16842-16848 (2020).
29. Gao, C. et al. Isostructural three- dimensional covalent organic frameworks. *Angew. Chem. Int. Ed.* **58**, 9770-9775 (2019).
30. Li, Z. et al. Three-dimensional ionic covalent organic frameworks for rapid, reversible, and selective ion exchange. *J. Am. Chem. Soc.* **139**, 17771-17774 (2017).
31. Ding, H. et al. An AIEgen-based 3D covalent organic framework for white light-emitting diodes. *Nat. Commun.* **9**, 5234 (2018).
32. Zhang, Y. et al. Three- dimensional anionic cyclodextrin- based covalent organic frameworks. *Angew. Chem. Int. Ed.* **56**, 16313-16317 (2017).
33. Ma, Y. et al. A dynamic three-dimensional covalent organic framework. *J. Am. Chem. Soc.* **139**, 4995-4998 (2017).
34. Lin, G., Ding, H., Yuan, D., Wang, B. & Wang, C. A pyrene-based, fluorescent three-dimensional covalent organic framework. *J. Am. Chem. Soc.* **138**, 3302-3305 (2016).
35. Li, H. et al. Three-dimensional covalent organic frameworks with dual linkages for bifunctional cascade catalysis. *J. Am. Chem. Soc.* **138**, 14783-14788 (2016).
36. Ma, J.-X. et al. Cage based crystalline covalent organic frameworks. *J. Am. Chem. Soc.* **141**, 3843-3848 (2019).
37. Wei, H. et al. The microwave-assisted solvothermal synthesis of a crystalline two-dimensional covalent organic framework with high CO_2 capacity. *Chem. Commun.* **51**, 12178-12181 (2015).
38. Gao, Q. et al. Covalent organic framework with frustrated bonding network for enhanced carbon dioxide storage. *Chem. Mater.* **30**, 1762-1768 (2018).
39. Li, Z. et al. Three-dimensional covalent organic framework with ceq topology. *J. Am. Chem. Soc.* **143**, 92-96 (2020).
40. Li, H. et al. Three-dimensional triptycene-based covalent organic frameworks with ceq or acs Topology. *J. Am. Chem. Soc.* **143**, 2654-2659 (2021).
